# Chemical Information Processing by a Responsive Chemical System

**DOI:** 10.1021/jacs.3c11414

**Published:** 2024-01-12

**Authors:** Luca Gabrielli, Lorenzo Goldin, Sushmitha Chandrabhas, Andrea Dalla Valle, Leonard J. Prins

**Affiliations:** Department of Chemical Sciences, University of Padova, via F. Marzolo 1, Padova 35131, Italy

## Abstract

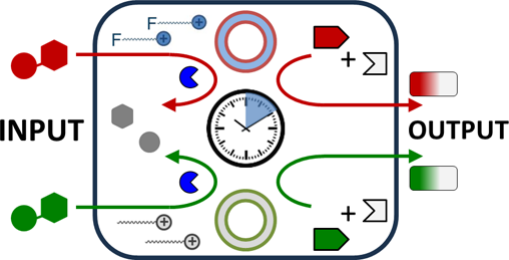

Nature has an extraordinary capacity to precisely regulate the chemical reactivity in a highly complex mixture of molecules that is present in the cell. External stimuli lead to transient up- and downregulation of chemical reactions and provide a means for a cell to process information arriving from the environment. The development of synthetic chemical systems with life-like properties requires strategies that allow likewise control over chemical reactivity in a complex environment. Here, we show a synthetic system that mimics the initial steps that take place when a natural signal transduction pathway is activated. Monophosphate nucleosides act as chemical triggers for the self-assembly of nanoreactors that upregulate chemical reactions between reagents present at low micromolar concentrations. Different nucleotides template different assemblies and hence activate different pathways, thus establishing a distinct connection between input and output molecules. Trigger-induced upregulation of chemical reactivity occurs for only a limited amount of time because the chemical triggers are gradually removed from the system by enzymes. It is shown that the same system transiently produces different output molecules depending on the chemical input that is provided.

## Introduction

**Figure 1:**
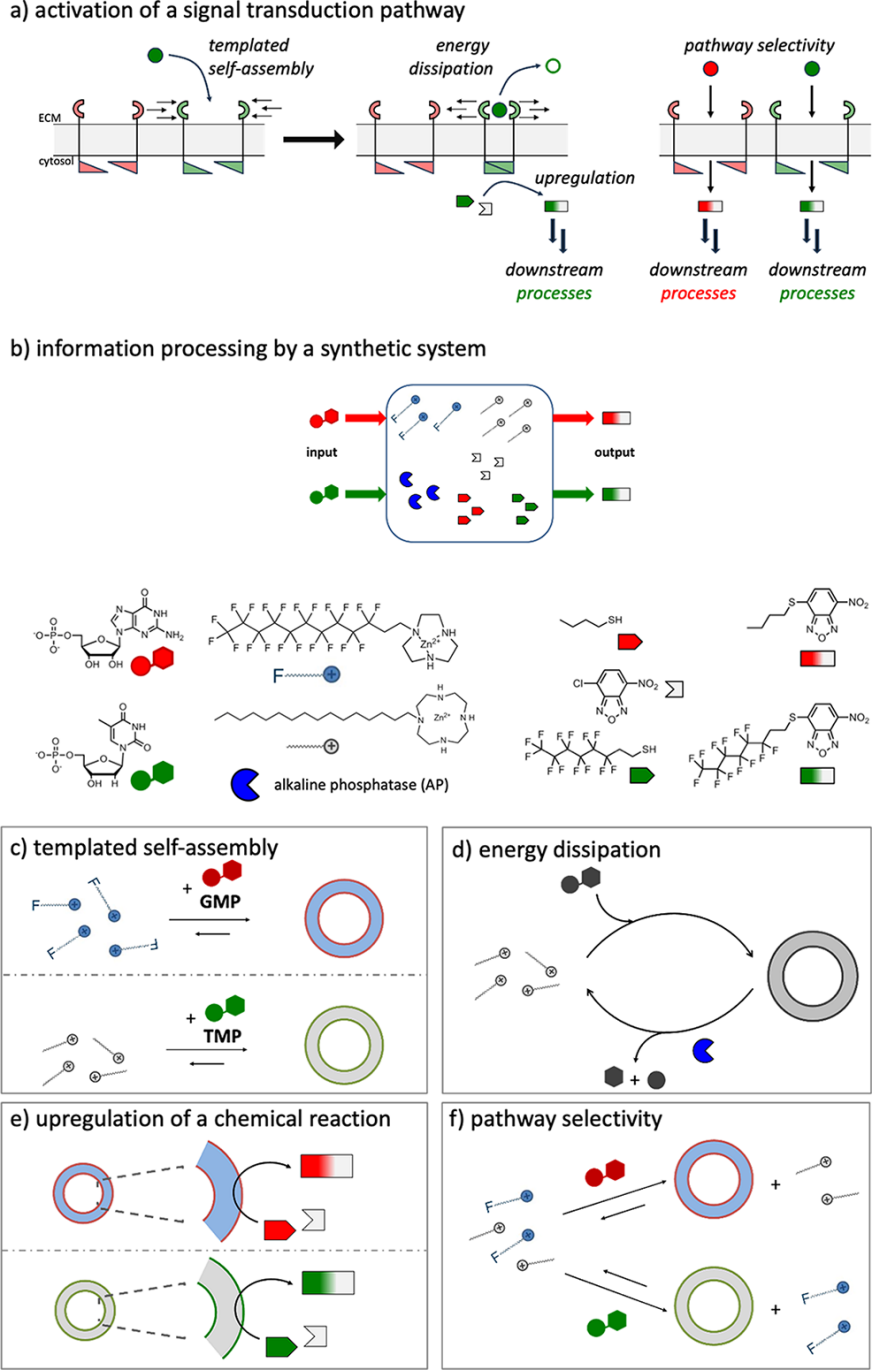
(a) Schematic representation of the processes involved in the activation of a natural signal transduction pathway. (b) Schematic illustration of chemical information processing by a dissipative synthetic system and chemical structures of all components. Cartoons highlighting the key processes interplaying in the system: (c) templated self-assembly, (d) energy dissipation, (e) upregulation, and (f) pathway selectivity. It is noted that throughout the manuscript, the assemblies are depicted as vesicles because of the analogy of these systems with a previously reported analogous systems for which a vesicular structure was determined.^27^ For the purpose of this study, it is not relevant whether the structures are actually vesicles or spherical droplets.

Inspired by the amazing properties that emerge from the chemistry of living systems, chemists have developed a strong interest in the design of synthetic systems with life-like properties.^1−3^ The transition toward life-like chemistry requires a paradigm shift in the way chemists control chemical reactivity in reaction mixtures. Traditionally, the aim of chemists is to produce specific target molecules with the highest possible yield.^4^ This is achieved by carrying out reactions using the smallest set of reagents required, mixed at a relatively high concentration (mM) under perfectly tuned experimental conditions and with little or no interest in the transient activation of chemical reactions. On the contrary, the “reaction flask” of nature, the cell, is loaded with thousands of different reagents, mostly at low concentrations (μM to nM) to avoid undesired reactions.^5^ To control chemical reactivity, nature extensively exploits catalysis and the increase in effective molarities by concentrating reagents in active sites.^6^ Different signal transduction pathways can be activated orthogonally by appropriate external stimuli (small molecules, light, etc.), which induce the self-assembly of active structures (e.g., protein dimers^7^ or clusters^8^) that play an essential role in the upregulation of specific regulatory pathways (Figure 1a).^9,10^ A salient feature is the responsiveness of the chemical reaction network: signal transduction pathways are transiently up- or downregulated^11^ in response to temporarily available external stimuli, which in time disappear as a result of passive (diffusion) or active (dissipative) processes.

The development of synthetic chemical systems that display emerging properties resulting from chemical reaction networks is attracting significant interest. A key feature in this development is the ability to control the reactivity in mixtures of chemicals, just as it happens in nature. Confinement of reagents on template molecules^12^ or in a confined molecular space^13^ has proven highly efficient to accelerate reactions and affect their outcomes. Molecules^14^ and reaction networks^15−17^ have been developed that are able to process molecular input to create output following Boolean logic or, more recently, also through adaptation at the systems level.^18^ Additionally, the development of chemically fueled self-assembly processes has provided a tool to transiently activate chemical processes.^19−21^

Here, we show a synthetic system able to process chemical information embedded in molecular triggers (input) for the transient upregulation of distinct chemical reactions (output) (Figure 1b).^22−26^ The system relies on the same processes that are involved in the activation of natural signal transduction pathways (Figure 1a, c–f): (i) templated self-assembly: selective molecular recognition between the triggers and recognition units present in the building blocks leads to the formation of a self-assembled structure, (ii) energy dissipation: the templating effect induced by the trigger has a limited duration because the trigger is gradually degraded, (iii) upregulation of chemical reactivity: the trigger-induced self-assembled structures accelerate the chemical reaction between reactants present in the system, and (iv) pathway selectivity: different triggers activate the formation of different assemblies, which accelerate different chemical reactions.

## Results and Discussion

To develop a minimalistic synthetic mixture that can transiently generate different output products in response to different chemical triggers, our approach was based on a gradual increase in complexity, by first focusing on the isolated key processes (Figure 1b–e) and then merging all processes in a single system.

### Templated Self-Assembly

Chemical information processing initiates with selective recognition between the trigger (TMP or GMP) and the surfactants **C_16_cyclen·Zn^2+^** and **C_10F_TACN·Zn**^**2**+^. We previously observed that the addition of guanosine monophosphate (GMP) to hydrocarbon surfactant C_16_TACN·Zn^2+^equipped with a 1,4,7-triazacyclononane (TACN)·Zn^2+^ headgroup led to the self-assembly of spherical structures. Interestingly, addition of any of the other nucleotides thymidine monophosphate (TMP), cytidine monophosphate (CMP), or adenosine monophosphate (AMP) at the same concentration did not lead to structure formation. On the other hand, the presence of 1,4,7,10-tetraazacyclododecane (cyclen)·Zn^2+^ in the hydrocarbon surfactant **C_16_cyclen·Zn^2+^** reduced selectivity for TMP, showing the selectivity of the interaction between nucleotides and macrocycle headgroup.^27^ Indeed, it is well documented that macrocyclic Zn^2+^-complexes selectively interact with nucleobases as a result of coordinative interactions between donor atoms on the nucleobase and the Zn^2+^ metal ion, combined with hydrogen bonds that involve the NH moieties present in the ligand.^28−30^ We showed that both assemblies were able to upregulate the same reaction between 4-chloro-7-nitrobenzofurazan (NBD-Cl) and octanethiol C_8H_SH by concentrating the apolar reagents in the hydrophobic part of the assemblies. These previous results indicated that this system can use chemical information embedded in the nucleotides to upregulate a chemical reaction through the activation of a self-assembly process, just as what happens when a natural signal transduction pathway is activated. However, an important discrepancy with natural systems is that both triggers upregulated the same chemical reaction because the C_16_TACN·Zn^2+^ and **C_16_cyclen·Zn^2+^** assemblies contained the same hydrophobic domain. We reasoned that differentiation of the apolar phase would be a possible solution. Different hydrophobic environments would be created if the surfactants were equipped with alkyl and fluoroalkyl hydrophobic chains. This would allow the templated self-assembly of structures with distinct hydrophobic domains that could take up reactants selectively.

**Figure 2:**
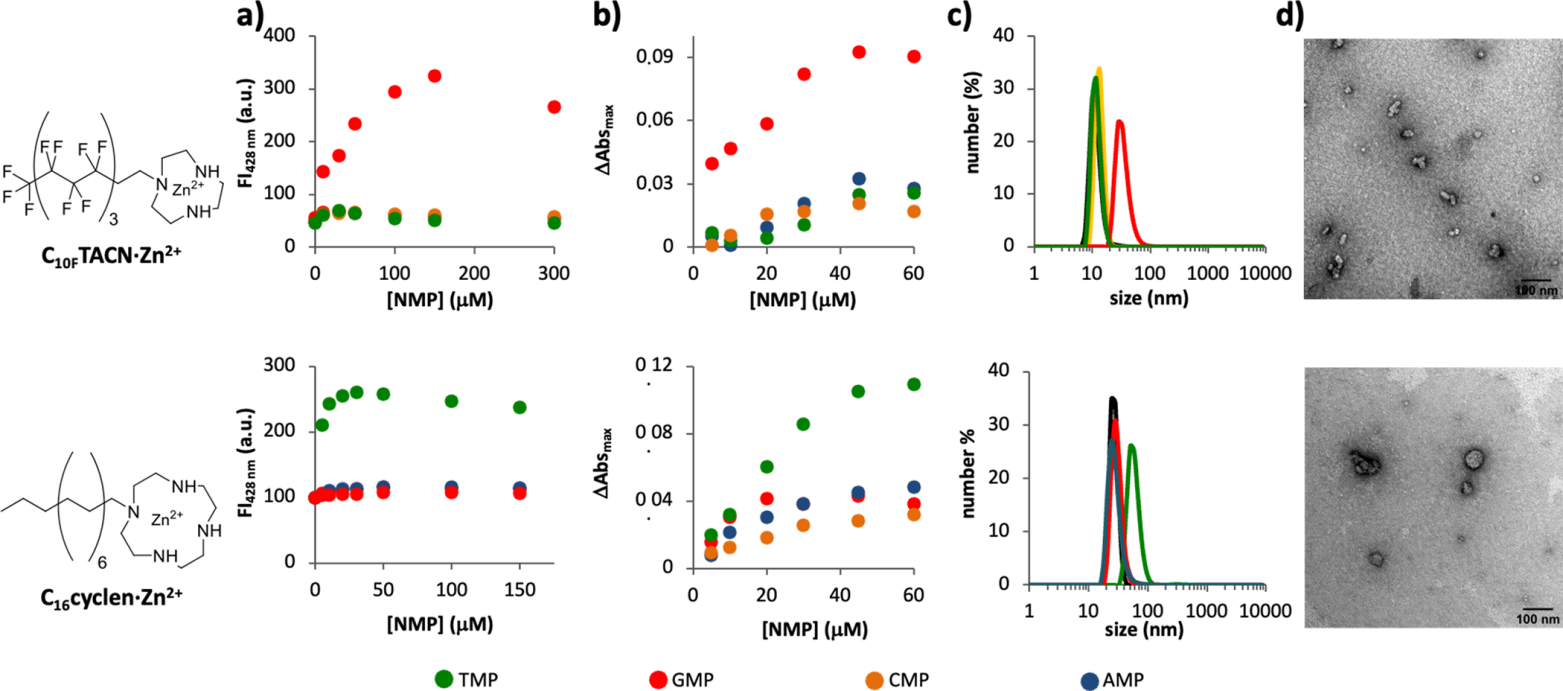
(a) Fluorescence intensity at 428 nm as a function of the *N*MP concentration (*N* = G, T, A, or C) at a fixed surfactant concentration of **C**_**10F**_**TACN·Zn**^**2+**^ (50 μM, top graph) or **C**_**16**_**cyclen·Zn**^**2+**^ (30 μM, bottom graph) in the presence of 1,6-diphenylhexatriene (DPH, 2.5 μM). (b) Difference in absorbance at the respective absorbance maxima (AMP = 259 nm, TMP = 268 nm, GMP = 253 nm, and CMP = 272 nm) of the nucleotides in the presence and absence of amphiphile as a function of the nucleotide concentration (0–60 μM) added to a solution of amphiphile (top graph: **C**_**10F**_**TACN·Zn**^**2+**^, 50 μM, bottom graph: **C**_**16**_**cyclen·Zn**^**2+**^, 30 μM). (c) Size distribution measured by dynamic light scattering (DLS) of the aggregates formed in the absence and presence of NMPs (top: 50 μM; bottom 30 μM) and a fixed amount of surfactants (top graph: **C**_**10F**_**TACN·Zn**^**2+**^, 50 μM, bottom graph: **C**_**16**_**cyclen·Zn**^**2+**^, 30 μM). (d) TEM images of a solution containing NMP (top: GMP (50 μM), bottom: TMP (30 μM)) and amphiphile (top: 50 μM; bottom: 30 μM). Samples were stained with a 2% uranyl acetate solution. Previously reported^27^ data for **C**_**16**_**cyclen·Zn**^**2+**^ (bottom graphs) are shown for comparison. Experimental conditions: [HEPES] = 5 mM, pH 7.0, *T* = 25 °C. The bottom part is reproduced with permission from ref (27). Copyright 2020, Wiley.

Hence, we synthesized a new surfactant, **C**_**10F**_**TACN·Zn**^**2+**^, in which the TACN-macrocycle is attached to a 2,2-tetrahydroperfluorododecyl chain (Figure 2). The assembly properties of **C**_**10F**_**TACN·Zn**^**2+**^ were initially studied through fluorescence titrations using 1,6-diphenyl-1,3,5-hexatriene (DPH) as a fluorescent probe that is sensitive to the formation of hydrophobic domains (Figure S1). The critical aggregation concentration (CAC) for **C**_**10F**_**TACN·Zn**^**2+**^ in aqueous buffer solution at pH 7.0 was around 100 μM, which is similar to the value previously determined for **C**_**16**_**cyclen·Zn**^**2+**^ (100 μM).^27^ We then investigated whether the presence of the fluoroalkyl chain would affect the nucleotide-selective templated assembly of the TACN-based surfactant. The ability of GMP to selectively stabilize assemblies of **C**_**10F**_**TACN·Zn**^**2+**^ was confirmed by titrating increasing amounts of either one of the monophosphate nucleosides *N*MP (*N*= T, G, C, and A) to a 50 μM solution of the fluorinated amphiphile in aqueous buffer at pH 7.0 (Figure 2a, top and Figure S2). Indeed, GMP induced the strongest increase in fluorescence, while the addition of the other nucleotides only led to a minimal signal increase. The ability of GMP to selectively template assembly formation was confirmed by other techniques. UV–vis spectra were measured for all monophosphate nucleosides in the 0–60 μM concentration range in the absence and presence of **C**_**10F**_**TACN·Zn**^**2+**^. When the difference in absorbance at the respective absorbance maxima of the nucleotides in the presence and absence of fluorinated amphiphiles was plotted as a function of the nucleotide concentration, the largest difference was observed for GMP (Figure 2b, top). Next, we used DLS to measure the hydrodynamic diameter of the assemblies present in a solution of **C**_**10F**_**TACN·Zn**^**2+**^ (50 μΜ) and 50 μΜ of *N*MP (*N*= T, G, C, and A) (Figure 2c, top). Stable assemblies with a well-defined diameter of 30 ± 10 nm were observed only in the presence of GMP. The assembly size was confirmed by transmission electron microscopy (TEM) images (Figure 2d, top). Notably, GMP-templated assemblies of **C**_**10F**_**TACN·Zn**^**2+**^ were sensibly smaller than those previously reported for TMP-templated assemblies of **C**_**16**_**cyclen·Zn**^**2+**^ (*d* = 55 ± 10 nm). For comparison, some of the previously reported key structural data^27^ for the TMP-selective templated formation of **C**_**16**_**cyclen·Zn**^**2+**^ are provided to demonstrate that the self-assembly of surfactants **C**_**10F**_**TACN·Zn**^**2+**^ and **C**_**16**_**cyclen·Zn**^**2+**^ can be selectively triggered by the nucleotides GMP and TMP, respectively.

### Energy Dissipation

Transient upregulation of a chemical process is a key feature of natural signal transduction pathways. For example, neural signal transmission relies on the release of a burst of the neurotransmitter acetylcholine (Ach) in the synaptic cleft, which activates the acetylcholine receptor (AchR) on the receiving cell. To avoid a permanent activation of the receptor by Ach, the synaptic cleft contains the enzyme acetylcholinesterase, which cleaves Ach at an astonishing rate of around 2 × 10^4^ molecules/s.^31^ In our system, a similar neutralization of the trigger is achieved by adding the enzyme alkaline phosphatase (AP) to the solution. AP cleaves monophosphate nucleosides in nucleoside and inorganic phosphate, which have a lower templating ability.^27,32^ Consequently, the addition of nucleotide under dissipative conditions installed by the presence of enzyme results in templated self-assembly with a lifetime that is determined by the rate at which the template is cleaved.

**Figure 3:**
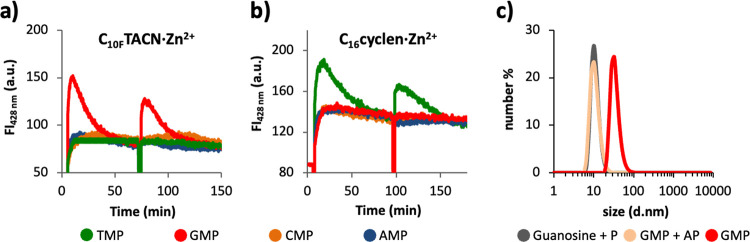
Fluorescence intensity at 428 nm following two repetitive additions of *N*MP (*N* = A, T, G, and C; a: 50 μM; b: 30 μM) to a solution of (a) **C**_**10F**_**TACN·Zn**^**2+**^ (50 μM) or (b) **C**_**16**_**cyclen·Zn**^**2+**^ (30 μM) and DPH (2.5 μM) in the presence of alkaline phosphatase (AP, 1 U/mL). (c) Size distribution measured by dynamic light scattering (DLS) of the aggregates formed with **C**_**10F**_**TACN·Zn**^**2+**^ (50 μM) in the presence of GMP (50 μM, red curve), AP (1 U/mL) and GMP (50 μM, pink curve), or guanosine and phosphate (50 μM each, gray curve). Experimental conditions: [HEPES] = 5 mM, pH 7, *T* = 25 °C. Panel (b) is reproduced with permission from ref (27). Copyright 2020, Wiley.

We studied therefore the templated self-assembly of **C**_**10F**_**TACN·Zn**^**2+**^ in the presence of the enzyme alkaline phosphatase (AP).^33^ Only upon the addition of GMP, but not TMP, CMP, or AMP, could we observe a transient increase in fluorescence intensity (Figure 3a and Figure S3). The half-life of the transient assemblies **C**_**10F**_**TACN·Zn**^**2+**^**·**GMP, measured as the time to reduce the fluorescence intensity from the maximum to 50% of the end value, was about 20 min, which is similar to the half-life previously reported for the assemblies **C**_**16**_**cyclen·Zn**^**2+**^**·**TMP (Figure 3b).^27^ The cycle could be reinitiated with a new addition of GMP (50 μM), showing the reversible nature of this process. Further evidence for the transient assembly formation under dissipative conditions was obtained from DLS (Figure 3c). Addition of GMP to a solution of **C**_**10F**_**TACN·Zn**^**2+**^ and AP resulted in the rapid formation of structures with a hydrodynamic diameter of 30 ± 10 nm, which decreased to a size of 10 ± 5 nm in around 60 min. This final value corresponded to that observed for a fluorinated surfactant in the presence of the waste products of GMP hydrolysis (guanosine + P_*i*_).

### Upregulation of Chemical Reactivity

The study of the surfactant **C**_**10F**_**TACN·Zn**^**2+**^ was motivated by the expectation that the presence of the fluoroalkyl chain could lead to selective uptake of reactants. Noncovalent fluorine–fluorine interactions drive the reciprocal affinity between highly fluorinated molecules. Fluorophilicity has been extensively exploited for the purification of fluorous-tagged molecules from other mixture components.^34−36^ The miscibility of hybrid structures with both hydrogenated and fluorinated chains has been investigated in phospholipids and glycolipids,^37,38^ in dendrimersomes,^39−41^ in polymers,^42^ in fibers,^43^ and nanoparticles.^44−46^ An important consequence of the potential immiscibility between alkyl and fluoroalkyl surfactants is the formation of two different hydrophobic domains, which in our system can be exploited for upregulating distinct chemical reactions.

**Figure 4:**
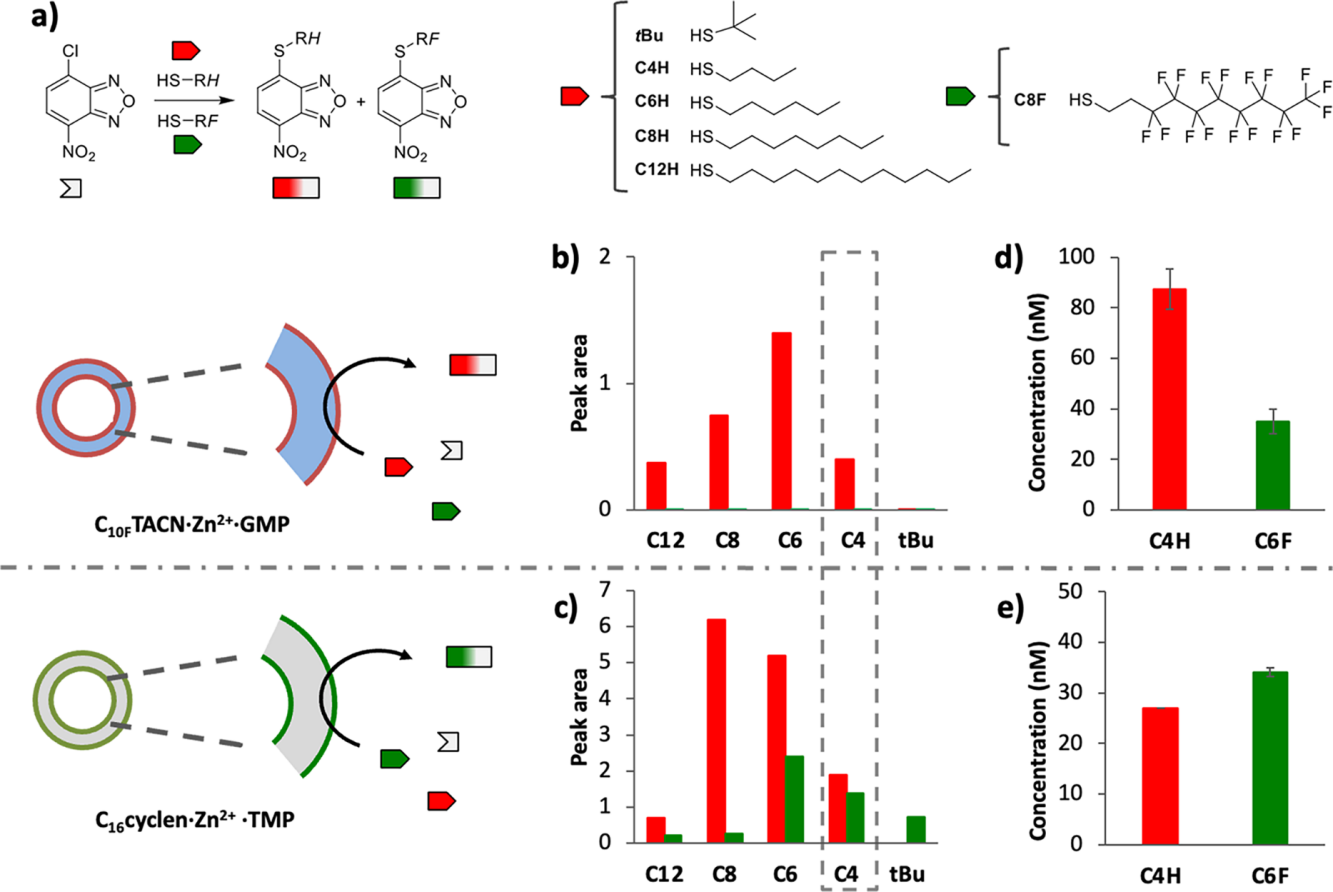
(a) Scheme showing the reactions between NBD-Cl and a set of thiols. HPLC peak area (Section 3.2.3) of NBD-SR products after 20 min of reaction in solutions containing NBD-Cl (2 μM), C_*n*H_SH (2 μM, red columns), and C_6F_SH (2 μM, green columns) in the presence of (b) GMP and **C**_**10F**_**TACN·Zn**^**2+**^ (50 μM each) or (c) TMP and **C**_**16**_**cyclen·Zn**^**2+**^ (30 μM each). Concentration of NBD-SC_4H_ and NBD-SC_6F_ after 20 min of reaction in solutions containing NBD-Cl (2 μM), C_4H_SH (1.5 μM), and C_6F_SH (4.5 μM) in the presence of (d) GMP and **C**_**10F**_**TACN·Zn**^**2+**^ (50 μM each) or (e) TMP and **C**_**16**_**cyclen·Zn**^**2+**^ (30 μM each) (Section 3.2.4). Error bars indicate the standard deviation calculated from the duplicate experiments. Experimental conditions: Zn^2+^ (600 μM), [HEPES] = 5 mM, pH 7.0, *T* = 25 °C.

We carried out a systematic investigation of the nucleophilic aromatic substitution reaction between NBD-Cl and two kinds of thiols containing either an alkyl or a fluoroalkyl chain. To assess whether the assemblies displayed selectivity, experiments were carried out in which the fluoroalkyl thiol (C_6_F_13_(CH_2_)_2_SH) competed with one thiol from a set of alkyl thiols (*t*BuSH and C_*n*_H_2*n*+1_SH with *n* = 4, 6, 8, and 12) for reaction with NBD-Cl (Figure 4a). These competition experiments were carried out separately for assemblies **C**_**10F**_**TACN·Zn**^**2+**^·GMP and **C**_**16**_**cyclen·Zn**^**2+**^·TMP. It is noted that in the absence of nucleotide, product formation occurred very slowly (Figures S23 and S24).

We first tested the reactivity of fluorinated and aliphatic thiols (2 μM each) with NBD-Cl (2 μM) in the presence of **C**_**10F**_**TACN·Zn**^**2+**^·GMP (50 μM). Surprisingly, HPLC analysis after 20 min revealed products from the reactions of alkyl thiols with NBD-Cl, while no products of the expected fluorinated thiols were detected (Section S3.2.3). Plotting of the products’ area as a function of the thiol’s chain length revealed a bell-shaped distribution with the strongest upregulation observed for C_6H_SH (Figure 4b). Interestingly, however, when the same reactions were performed in the presence of **C**_**16**_**cyclen·Zn**^**2+**^·TMP (30 μM), the product of reaction between the fluorinated thiol C_6F_SH and NBD-Cl was indeed detected, even if in most of the cases it was present in trace amounts and with the alkyl thiol in all cases being the dominant product. It is also noted that in general, more products had formed and that the strongest upregulation was observed for C_8H_SH (Figure 4c).

Considering that the highest amount of C_6F_S-NBD relative to the alkyl thiol was observed for C_4H_SH, we selected the C_6F_SH/C_4H_SH-couple for optimization by changing the relative concentrations of reagents. A selective response could indeed be obtained with a reaction mixture composed of 4.5 μM C_6F_SH, 1.5 μM C_4H_SH, and 2 μM of NBD-Cl. In the presence of assemblies **C**_**10F**_**TACN·Zn**^**2+**^·GMP, we observed a selective upregulation of the reaction leading to alkyl product C_4H_S-NBD (C_4H_S-NBD/C_6F_S-NBD = 2.5; Figure 4d), whereas a selectivity in favor of the fluoroalkyl product C_6F_S-NBD was observed in the presence of assemblies **C**_**16**_**cyclen·Zn**^**2+**^·TMP (C_6F_S-NBD/C_4H_S-NBD = 1.2; Figure 4e).

Even if the system displays selectivity in product formation, it is opposed to our expectations: it is in favor of the alkyl thiol for the fluorinated assemblies and in favor of the fluoroalkyl thiol for the alkyl assemblies. If on one hand, fluorophilicity is fundamental for dictating the self-assembly behavior of the amphiphiles (see the next section), then on the other hand, these results indicate that fluorophilicity does not play a key role regarding selective uptake. We speculate that selectivity originates from the different natures of the hydrophobic domains regarding the thickness of the hydrophobic layer (larger in **C**_**16**_**cyclen·Zn**^**2+**^·TMP than in **C**_**10F**_**TACN·Zn**^**2+**^·GMP), curvature, and rigidity (which is higher in fluorinated species). Indeed, alkyl and fluoroalkyl chains have not only different conformations and space requirements but also distinct dynamics: fluorinated chains are usually stiff and more rodlike, while aliphatic ones are more flexible. Consequently, the activation energy for many dynamic processes is usually lower for the alkyl-chains than for the fluoroalkyl chains.^47^

Regarding the experiments, it is of relevance to note that a large excess of Zn^2+^-ions (600 μM) was added to minimize the formation of secondary products between NBD-Cl and the secondary amines of the headgroup macrocycles.^33^ Nonetheless, as we previously observed in the case of **C**_**16**_**cyclen·Zn**^**2+**^·TMP, prolonged reaction times caused the formation of significant amounts of product originating from a reaction between NBD-Cl and cyclen (Figure S22).^27^ Interestingly, as we will show later, the amount of side products observed in the final system containing both surfactants and all reactants was reduced by 70%. Successively, we will indeed show that an increase in complexity of the system minimizes the formation of side products (for a discussion, see also the Supporting Information, Section S3.2.8).

### Pathway Selectivity

Chemical information processing by our system requires an orthogonality of both pathways leading to the respective products C_6F_S-NBD and C_4H_S-NBD. In this section, we provide evidence for the selective assembly of **C**_**16**_**cyclen·Zn**^**2+**^ and **C**_**10F**_**TACN·Zn**^**2+**^ in a mixture of both surfactants when the appropriate trigger is added and the subsequent selective upregulation of the chemical reaction corresponding to the respective assembly.

**Figure 5:**
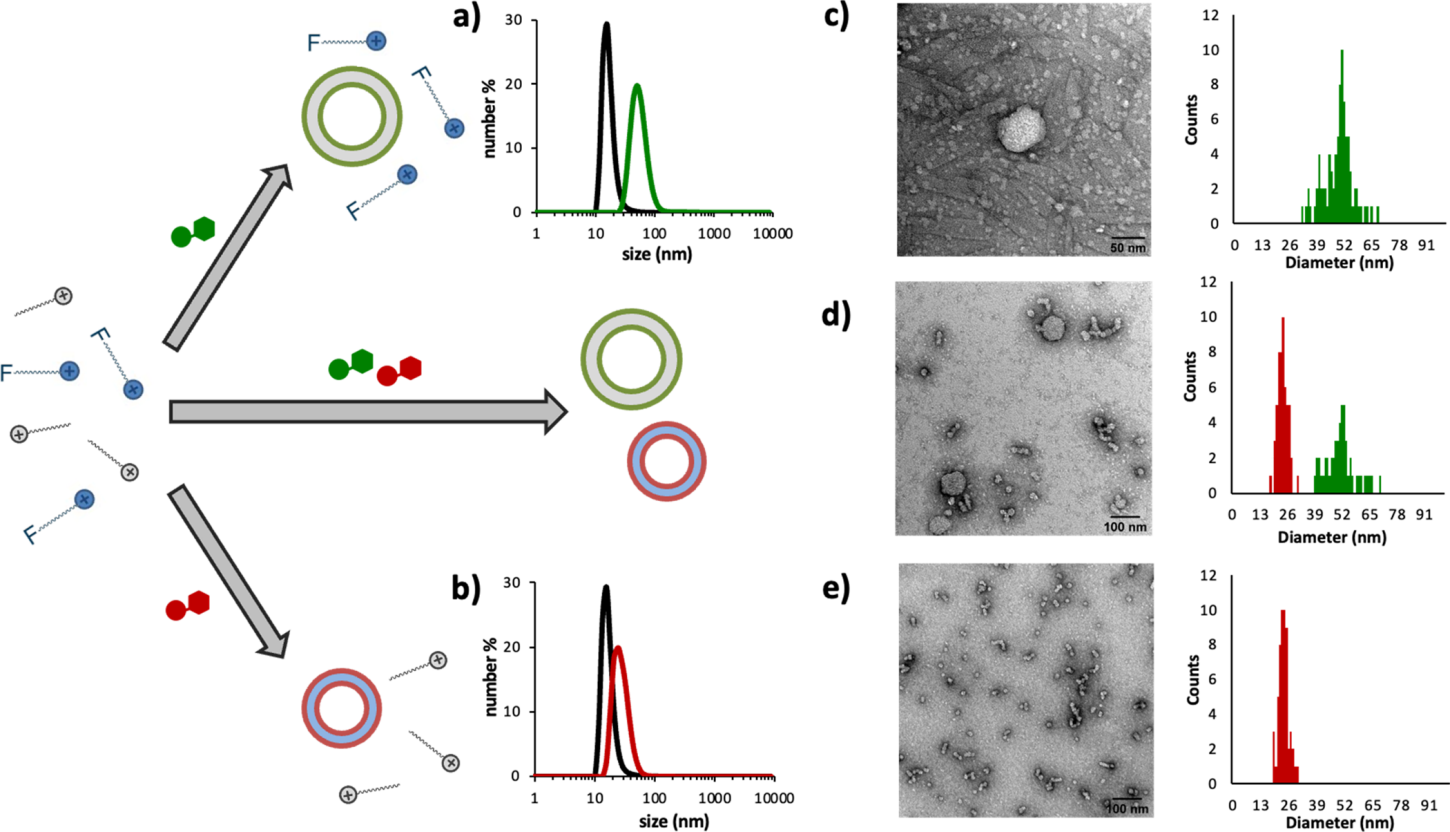
Size distribution measured by dynamic light scattering (DLS) of the aggregates formed in a solution containing both (a) **C**_**16**_**cyclen·Zn**^**2+**^ (30 μM) and (b) **C**_**10F**_**TACN·Zn**^**2+**^ (50 μM), without nucleotides (black curves) and upon addition of GMP (50 μM, red curve) or TMP (30 μM, green curve). TEM images and population distribution of a solution containing both **C**_**16**_**cyclen·Zn**^**2+**^ (30 μM) and **C**_**10F**_**TACN·Zn**^**2+**^ (50 μM), in the presence of (c) GMP (50 μM), (d) GMP and TMP (respectively, 50 and 30 μM), or (e) TMP (30 μM). Freshly prepared samples were stained with 2% uranyl acetate solution. Experimental conditions: [HEPES] = 5 mM, pH 7.0, *T* = 25 °C.

The most important feature for distinguishing the formation of assemblies **C**_**16**_**cyclen·Zn**^**2+**^·TMP and **C**_**10F**_**TACN·Zn**^**2+**^·GMP is their different size. **C**_**16**_**cyclen·Zn**^**2+**^·TMP assemblies have a diameter of around 55 nm (Figure 2c,d bottom), whereas the **C**_**10F**_**TACN·Zn**^**2+**^·GMP assemblies have a significantly smaller size (about 30 nm; Figure 2c,d top). Therefore, we studied the nucleotide-templated self-assembly in a mixture of both surfactants **C**_**16**_**cyclen·Zn**^**2+**^ and **C**_**10F**_**TACN·Zn**^**2+**^ with DLS and TEM. DLS measurements were performed after adding 30 μM TMP or 50 μM GMP to an aqueous buffer solution containing both **C**_**16**_**cyclen·Zn**^**2+**^ (30 μM) and **C**_**10F**_**TACN·Zn**^**2+**^ (50 μM; Figure 5a,b). Reassuringly, we observed that the size of the assemblies after TMP addition corresponded to the size of the assemblies expected for **C**_**16**_**cyclen·Zn**^**2+**^·TMP (55 ± 10 nm). On the other hand, the size of the assemblies when GMP was added to the mixture was consistent with the size of **C**_**10F**_**TACN·Zn**^**2+**^·GMP (30 ± 10 nm). TEM images confirmed these observations (Figure 5c–e and Figures S7 and S8).

In addition, TEM analyses were performed on samples containing both surfactants and nucleotides (30 μM **C**_**16**_**cyclen·Zn**^**2+**^·TMP and 50 μM **C**_**10F**_**TACN·Zn**^**2+**^·GMP). Regardless of the order of nucleotides’ addition, we observed two distinct populations of assemblies: large spherical structures, which corresponded to the **C**_**16**_**cyclen·Zn**^**2+**^·TMP assemblies (52 ± 10 nm), and a set of smaller aggregates (25 ± 10 nm) corresponding to the **C**_**10F**_**TACN·Zn**^**2+**^·GMP assemblies (Figure 5d and Figures S9 and S10). These results indicate that the chemical input leads to a high level of self-sorting in the mixed system.

The next step was to verify whether selective activation of the self-assembly process also activated the corresponding reaction pathway, which would establish a link between each input molecule and a specific output product. Hence, mixtures of both surfactants were prepared containing NBD-Cl (2 μM) and both the hydrogenated and fluorinated thiols (4.5 μM C_6F_SH and 1.5 μM C_4H_SH).

**Figure 6:**
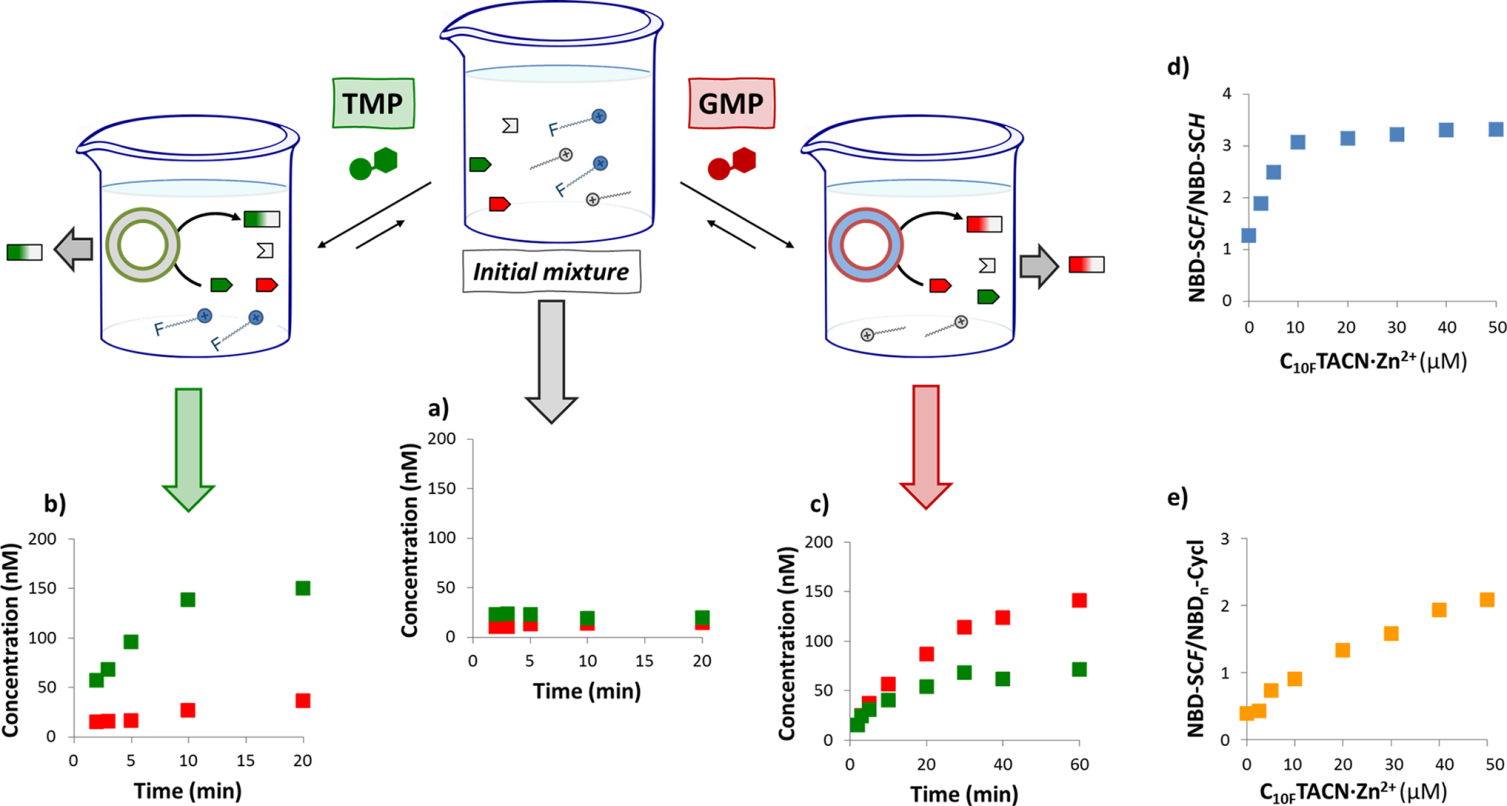
(a) Concentration of products NBD-SC_6F_ and NBD-SC_4H_ (respectively, red and green squares) as a function of time, for a solution containing both **C**_**10F**_**TACN·Zn**^**2+**^ (50 μM) and **C**_**16**_**cyclen·Zn**^**2+**^ (30 μM), NBD-Cl (2 μM), C_4H_SH (1.5 μM), and C_6F_SH (4.5 μM) without nucleotides, in the presence of (b) TMP (30 μM) or (c) GMP (50 μM). Error bars indicating the standard deviation are calculated from triplicate experiments and are smaller than the square indicators. Ratio between the HPLC peak area of (d) NBD-SC_6F_ and NBD-SC_4H_ or (e) NBD-SC_6F_ and secondary products given by reactions of NBD-Cl and cyclen, as a function of **C**_**10F**_**TACN·Zn**^**2+**^, for a solution of **C**_**16**_**cyclen·Zn**^**2+**^ (30 μM), NBD-Cl (2 μM), C_4H_SH (1.5 μM), and C_6F_SH (4.5 μM) after 20 min of reaction. Chromatograms are provided in Section 3.2.6. Experimental conditions: Zn^2+^ (600 μM), [HEPES] = 5 mM, pH 7.0, *T* = 25 °C.

In the absence of any input molecules, only traces of products were detected (Figure 6a and Figure S25). However, the addition of TMP led to the self-assembly of **C**_**16**_**cyclen·Zn**^**2+**^ to give nanoreactors that selectively upregulated the formation of C_6F_S-NBD (Figure 6b and Figure S26). On the other hand, an opposite selectivity was observed when GMP was added instead of TMP. In this case, the system responded by forming fluorinated nanoreactors (**C**_**10F**_**TACN·Zn**^**2+**^·GMP), which upregulated the formation of C_4H_S-NBD to a higher extent compared to C_6F_S-NBD (Figure 6c and Figure S27). The systems’ response to the addition of different triggers demonstrates unequivocally that each trigger activates a specific pathway leading to different products.

Interestingly, however, comparison of the selectivities in these experiments with those obtained when the surfactants were separated (Figure 4 and Supporting Information, Section S3.2.4) showed that the performance had improved. Defining the selectivity as the ratio between the desired and undesired product, it can be observed that when GMP is used as input, both single-surfactant and mixed systems give similar results (respectively, 2.5:1 and 2.0:1 for the C_4H_S-NBD:C_6F_S-NBD ratios). However, using TMP as an input molecule, we observe a large increase in selectivity since the C_6F_S-NBD:C_4H_S-NBD ratio increases from 1.2:1 (just **C**_**16**_**cyclen·Zn**^**2+**^; Figure 4e) to 4.1:1 in the mixed system. Additionally, increasing the complexity of the system by mixing the two surfactants not only increased the pathway’s selectivity but also strongly decreased the amount of undesired secondary products (Figure S28).

To provide an explanation, we focused on the selectivity of the TMP-triggered pathway because this pathway showed the strongest increase in selectivity. The only difference between the single-surfactant and the mixed system is the copresence of unassembled surfactant **C**_**10F**_**TACN·Zn**^**2+**^ in solution. Hence, we followed the reactions between NBD-Cl and both thiols in solutions containing **C**_**16**_**cyclen·Zn**^**2+**^·TMP assemblies (30 μM) and increasing amounts of **C**_**10F**_**TACN·Zn**^**2+**^ (0–50 μM). Plotting the ratio between C_6F_S-NBD and C_4H_S-NBD as a function of the concentration of **C**_**10F**_**TACN·Zn**^**2+**^ indeed showed a gradual increase in selectivity (Figure 6d and Figure S28). The presence of just 10 μM **C**_**10F**_**TACN·Zn**^**2+**^ is sufficient to triplicate the selectivity for the fluorinated product. In addition, increasing the concentration of **C**_**10F**_**TACN·Zn**^**2+**^ up to 50 μM causes also a gradual reduction in the relative amount of side products NBD_*n*_-cyclen (Figure 6e). It is important to consider that the concentration range of 10–50 μM is close to the CAC of **C**_**10F**_**TACN·Zn**^**2+**^ (Figure S1), and the presence of hydrophobic reagents may stabilize the small micelles. It is known that micellar aggregates can participate in solubilizing and sequestering reagents or products (also without upregulating reactions), therefore adding new possible equilibria that influence the overall distribution of compounds in the complex system.^48^ Considering the strong selectivity of **C**_**10F**_**TACN·Zn**^**2+**^·GMP assemblies to upregulate alkyl products, it is licit to hypothesize that fluorinated micelles preferentially accommodate C_4H_SH, thus subtracting it from competition with C_6F_SH for uptake by **C**_**16**_**cyclen·Zn**^**2+**^·TMP. Increasing the amount of **C**_**10F**_**TACN·Zn**^**2+**^·might also sequester the apolar reactant NBD-Cl, hence leading to the decrease in the secondary products NBD_*n*_-cyclen.

### Information Processing

**Figure 7:**
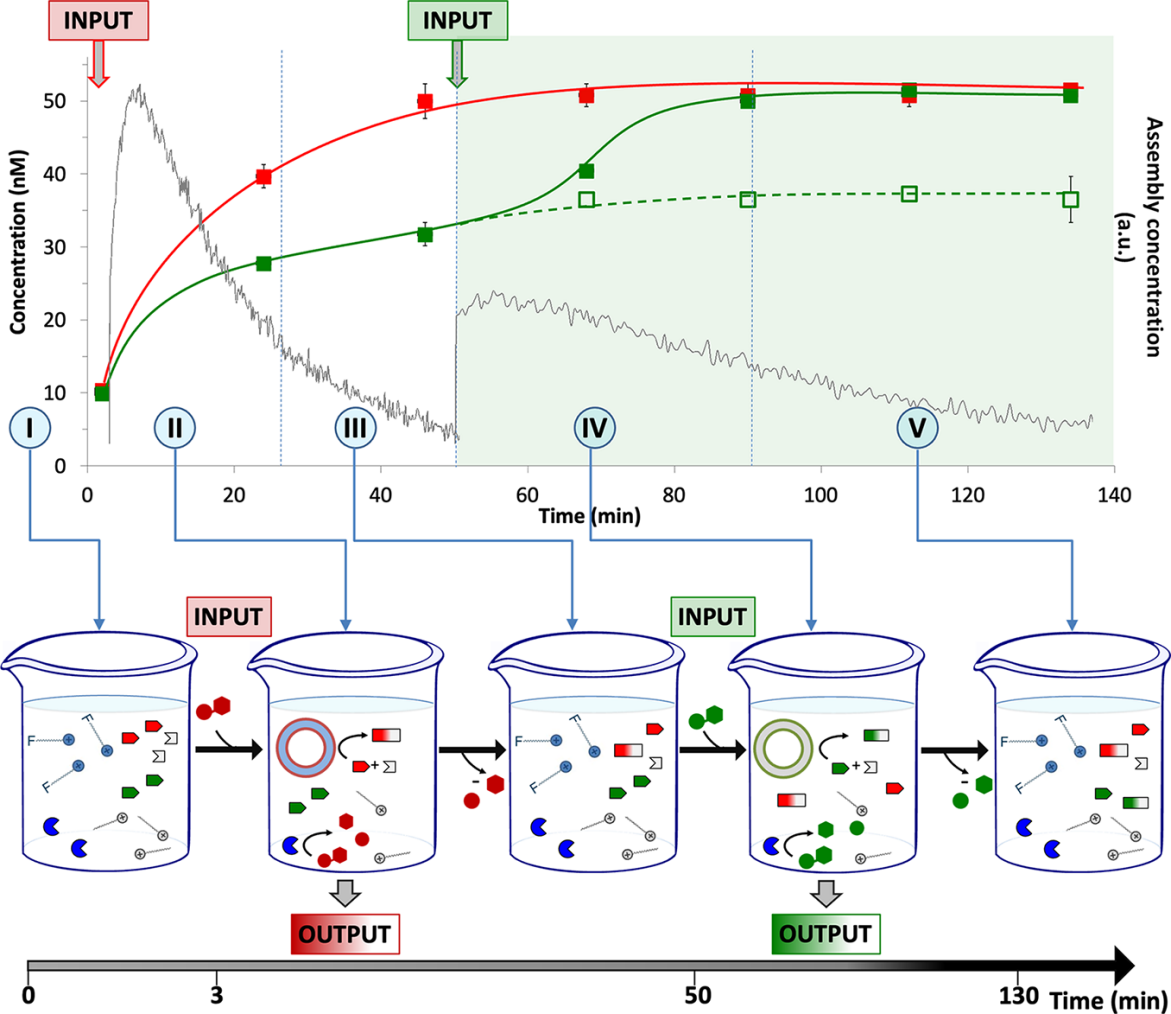
Concentration of C_4H_S-NBD (red squares) and C_6F_S-NBD (green squares) as a function of time for a solution containing both **C**_**10F**_**TACN·Zn**^**2+**^ (50 μM) and **C**_**16**_**cyclen·Zn**^**2+**^ (30 μM), NBD-Cl (2 μM), C_4H_SH (1.5 μM), C_6F_SH (4.5 μM), and AP (1 u/mL). GMP (50 μM) was added at *t* = 0, and the green region represents the time frame after the addition of TMP (30 μM) at *t* = 50 min. Hollow dots show the peak area of C_6F_S-NBD without the addition of TMP. The concentrations were determined via HPLC chromatography (Section S3.2.7). Error bars indicate the standard deviations calculated from four measurements for the first three points. After that, TMP was added to two samples, and the error bars indicate the standard deviation calculated from the duplo measurements. Lines are added to guide the eye. The gray lines are the fluorescence intensities reproduced from Figure 3 and have been added to provide an indication of the lifetime of the assemblies under the experimental conditions. The timeline is divided in five sectors, which are described in the flasks below: I, III, and V represent resting states, and II and IV are intermittent sectors in which the chemical reactivity is upregulated because of the addition of GMP and TMP, respectively. Experimental conditions: Zn^2+^ (600 μM), HEPES buffer (pH 7.0, 5 mM) at 25 °C.

Finally, we combined all separate processes in a single system by mixing the two surfactant molecules **C**_**16**_**cyclen·Zn**^**2+**^ and **C**_**10F**_**TACN·Zn**^**2+**^, three reactants C_6F_SH, C_4H_SH, and 4-cloro-7-nitrobenzofurazan (NBD-Cl), and the enzyme alkaline phosphatase (AP). Under the experimental conditions, products C_4H_S-NBD and C_6F_S-NBD form very slowly as a result of a nucleophilic aromatic substitution reaction between the thiols and NBD-Cl (Figure 7, I). The addition of the nucleotide guanosine monophosphate (GMP) to this mixture templates the self-assembly of **C**_**10F**_**TACN·Zn**^**2+**^ into spherical assemblies that accelerate the formation of product C_4H_S-NBD with selectivity over that of C_6F_S-NBD (Figure 7, II). Yet, under the action of the enzyme AP, the concentration of GMP gradually decreases in time and the waste products guanosine and inorganic phosphate are unable to maintain the structural stability of the assemblies. Upon depletion of GMP, the assemblies dissociate, and after around 50 min, the reaction stops (Figure 7, III). At this point, the addition of a different nucleotide, thymidine monophosphate (TMP), triggers the self-assembly of surfactant **C**_**16**_**cyclen·Zn**^**2+**^ (Figure 7, IV). Also, these assemblies upregulate a chemical reaction but as a result of the different hydrophobic domains composed of hydrocarbon chains, this time with an inverted selectivity for C_6F_S-NBD. The lifetime of these assemblies is again limited because of enzyme activity, and after an additional 50 min, the system returns to the original unassembled resting state but now also enriched in C_6F_S-NBD (Figure 7, V).

Comparison of the performance of the final system (Figure 7) with the same system at equilibrium, i.e., in the absence of enzymes (Figure 6), shows that overall, a much lower amount of product is formed, which is caused by the limited lifetime of the assemblies. This leaves less time for the reactions to occur. For instance, under equilibrium conditions, the addition of GMP to a mixed system caused the formation of 140 nM of C_4H_S-NBD (see Figure 6c), while under dissipative conditions, the production of C_4H_S-NBD is reduced by 65% (see Figure 7). Indeed, analyzing the intensity of the fluorescent signal produced by DPH in **C**_**10F**_**TACN·Zn**^**2+**^·GMP assemblies under dissipative conditions (Figure 3), we can estimate that the assemblies are reduced by 50% within 20 min. Moreover, in the same figure, we can observe that the second addition of GMP causes a 40% lower increase in fluorescence, suggesting that the subsequent addition of trigger molecules causes the formation of an even further reduced number of assemblies. The reduced lifetime of the nanoreactors under dissipative conditions, together with the decreased efficiency in forming assemblies after the first nucleotide addition, can explain the small amount of C_6F_S-NBD formed and the absence of C_4H_S-NBD products formed after the second addition of TMP in the final system (Figure 7).

## Conclusions

In conclusion, we have developed a synthetic chemical system capable of processing chemical information embedded in monophosphate nucleosides for the production of specific output molecules. The same system can generate different output products depending on the chemical information that is provided. Chemical reactions are upregulated for a limited amount of time, which is determined by the lifetime of the chemical trigger under the dissipative conditions. The approach relies on the trigger-induced transient self-assembly of nanoreactors able to upregulate specific synthetic pathways by taking up reactants and enhancing their effective concentrations. The system relies on a combination of key concepts from supramolecular chemistry: molecular recognition, self-sorting, confined space, and energy dissipation. Remarkably, we observed that an increase in complexity of the system resulting from the simultaneous presence of both surfactants led to an improvement in terms of selectivity compared to the isolated systems. The work presented here provides a glimpse into a possible future of synthetic chemistry as an information science in which synthetic networks are able to process chemical information.

## Supporting Information Available

Materials and instrumentation, experimental procedures, and additional supporting data (PDF)
