# Supplementary material for: Chemical Information Processing by a Responsive Chemical System
Source: J Am Chem Soc. 2024 Jan 12;146(3):2080–8. doi: 10.1021/jacs.3c11414 (PMC10811666; doi:10.1021/jacs.3c11414)
Supplement: Supplementary file 1 — ja3c11414_si_001.pdf [file ja3c11414_si_001.pdf]

Supporting Information

Chemical information processing by a responsive chemical system

Luca Gabrielli*, Lorenzo Goldin, Sushmitha Chandrabhas, Andrea Dalla Valle, Leonard J. Prins*

Department of Chemical Sciences, University of Padova, via F. Marzolo 1, Padova 35131, Italy

Table of Contents

**1**	**General Experimental Procedures**	**S2**
**2**	**Characterization of aggregates**	**S4**
2.1	Isolated systems	S4
2.1.1	Single-surfactant system: **C_{10F}TACN** aggregation studies	S4
2.1.2	TEM of **C_{16}Cyclen·Zn^{2+}**	S8
2.1.3	TEM of **C_{10F}TACN·Zn^{2+}**	S9
2.2	Mixed systems	S10
2.2.1	TEM of **C_{10F}TACN·Zn^{2+}** + **C_{16}Cyclen·Zn^{2+}** + TMP	S10
2.2.2	TEM of **C_{10F}TACN·Zn^{2+}** + **C_{16}Cyclen·Zn^{2+}** + GMP	S11
2.2.3	TEM of **C_{10F}TACN·Zn^{2+}** + **C_{16}Cyclen·Zn^{2+}** + GMP + TMP	S12
2.2.4	TEM of **C_{10F}TACN·Zn^{2+}** + **C_{16}Cyclen·Zn^{2+}** + TMP + GMP	S13
**3**	**UHPLC chromatography**	**S14**
3.1	Materials and general methods	S14
3.2	Chromatograms	S15
3.2.1	Reference chromatograms for C_{4H}S-NBD and C_{6F}S-NBD	S15
3.2.2	Calibration curves	S18
3.2.3	Chromatograms for the screening study reported in Figure 4b and 4c	S19
3.2.4	Chromatograms used for Figure 4d and 4e	S23
3.2.5	Control experiments in the absence of nucleotide	S25
3.2.6	Chromatograms used for Figure 6	S27
3.2.7	Chromatograms used for Figure 7	S31
3.2.8.	Formation of side products	S34
**4**	**Synthesis and characterisation**	**S36**
4.1	Synthesis of surfactant **C_{16}Cyclen**	S36
4.2	Synthesis of surfactant **C_{10F}TACN**	S38
4.2.1	Synthesis of **2**	S38
4.2.2	Synthesis of **C_{10F}TACN**	S41
4.3	Synthesis of **C_{4HS}-NBD**	S44
4.4	Synthesis of **C_{6FS}-NBD**	S48
**5**	**References**	**S53**

1. General Experimental Procedures

The reagents, materials and solvents used in the synthesis were bought from international suppliers and used without prior purification. Thin layer chromatography was carried out using Merck 60 F254 silica gel precoated aluminium plates. Column chromatography was carried out manually on Macherey-Nagel silica gel 60 (70-230 mesh). The $Zn(NO_3)_2$-stock solution was standardized using EDTA following standard procedures. The stock solutions were prepared both by weight and UV-vis spectroscopy using the molar extinction coefficients: ε_{259}(AMP) = 15400 $M^{-1}cm^{-1}$, ε_{268}(TMP) = 9600 $M^{-1}cm^{-1}$, ε_{253}(GMP) = 13700 $M^{-1}cm^{-1}$, ε_{272}(CMP) = 8700 $M^{-1}cm^{-1}$. Each weight measurement was repeated three times on an analytical balance accurate to one-hundredth of a milligram, and the three results were averaged. The enzyme was dissolved in the required amount of milli-Q H_2O, then it was partitioned into several 0.5 mL vials and stored at -20°C. This allows to minimise the enzyme degradation caused by defrost cycles.

All the stocks solutions were prepared in Milli-Q water except for **NBD-Cl** (acetonitrile), **R_HSH**, **R_FSH**, **R_FNH_2** (MeOH). All the synthesised compounds and the stock solutions of **NBD-Cl**, nucleotides, C_{16}cyclen and **R_FNH_2** were stored at -20 °C. The stock solution of **C_{10F}TACN** was stored at room temperature. The stock solution of HEPES and $Zn(NO_3)_2$ were stored at 4 °C. The stock solutions of thiols (**R_HSH**, **R_FSH**) were prepared every morning before starting the experiment in deoxygenated methanol.

Fluorescence measurements were performed using a Varian Cary Eclipse fluorescence spectrophotometer equipped with a thermostated cell holder.

NMR spectroscopy experiments were carried out on Bruker AVI250, AVI400, DPX400, AVIII400, 500 MHz AVIII HD Smart Probe, AV600 MHz Cryo spectrometers using the solvent residual signal as the internal standard. All chemical shifts (δ) are reported in ppm and coupling constants given in Hz.

HRMS mass spectra were obtained with a Mariner Applied Biosystem (API-TOF) mass spectrometer (MeOH, 0.5% formic acid). TEM images were recorded on a Jeol 300 PX electron microscope. First the grid was placed on a drop of freshly prepared sample solution for 1 min and then, a drop of uranyl acetate (2 %) solution was added for 30 s. Then the solvent in excess was carefully removed with a clean tissue before imaging of the stained grid.

UV-Vis spectra were registered through a Varian Cary50 spectrophotometer equipped with a 14 slots thermostated cuvettes carrier. Spectra were acquired in Scan mode. Spectra were then exported, analysed, and plotted in Excel. All analysis were done with 1 mL cuvettes.

UHPLC kinetic experiments were conducted with an Agilent 1290 Infinity, equipped with a ZORBAX SB-C3 column (Rapid Resolution HT 2.0 × 100 mm 1.8-Micron 600Bar), UV-Vis Detector Agilent G4212-60008 (10 mm; V(σ) 1.0 µL; Maximum Pressure 60 Bar), and mass spectrometer Agilent 6130 Quadrupole LC/MS.

2. Characterization of aggregates

2.1 Isolated systems

2.1.1 Single-surfactant system: C_{10F}TACN aggregation studies

<u>Critical aggregation concentration (CAC)</u>

The CAC of **C_{10F}TACN·Zn^{2+}** was determined by adding increasing amounts of the surfactant to a solution of 1,6-diphenyl-1,3,5-hexatriene (DPH) (2.5 µM) in aqueous buffer ([HEPES] = 5 mM, pH 7.0). The used stock solutions are described in section 1. DPH is an apolar fluorescence probe that is weakly fluorescent in most polar solvents, but becomes intensely fluorescent in hydrophobic environments. The CAC can be identified as the point where there is a significant change in fluorescence intensity due to the solubilisation of the DPH into the hydrophobic layer of the self-assembled structures.

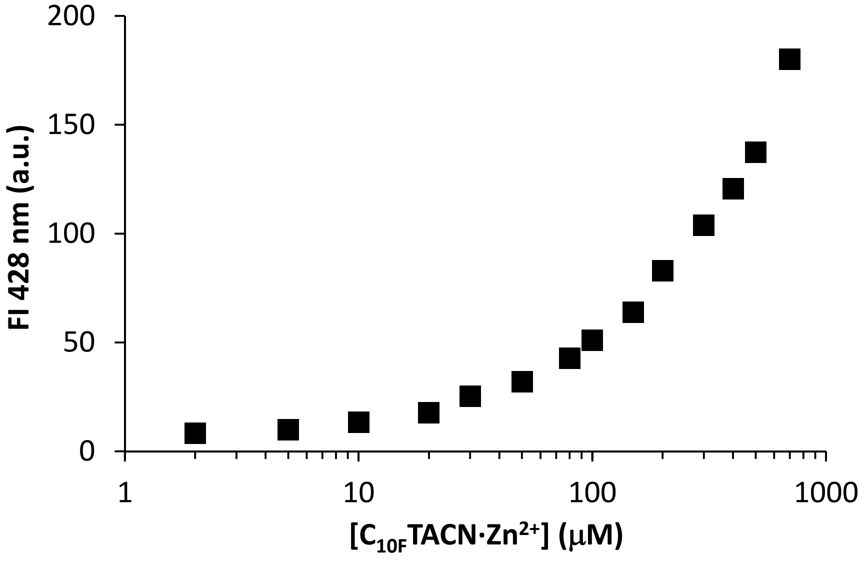

Figure S1. Fluorescence intensity at 428 nm (a.u.) as a function of the amount of **C_{10F}TACN·Zn^{2+}** added to an aqueous buffer solution containing DPH (2.5 µM). Experimental conditions: [HEPES] = 5 mM, pH 7.0, T = 25 °C, Excitation wavelength = 355 nm, slit width (ex/em=5/10 nm).

Selectivity studies

In addition to the data reported in Figure 3 (top), the selective interaction between nucleotides and the amphiphile **C_{10F}TACN·Zn^{2+}** was studied by adding increasing amount of **C_{10F}TACN·Zn^{2+}** to a solution of DPH (2 μM) and nucleotides monophosphate (*N*MP, 30 μM) in aqueous buffer ([HEPES] = 5 mM, pH 7.0). Stock solutions are described in section 1. The largest decrease in the CAC was observed for GMP, thus confirming the selective interaction with GMP over TMP, AMP and CMP.

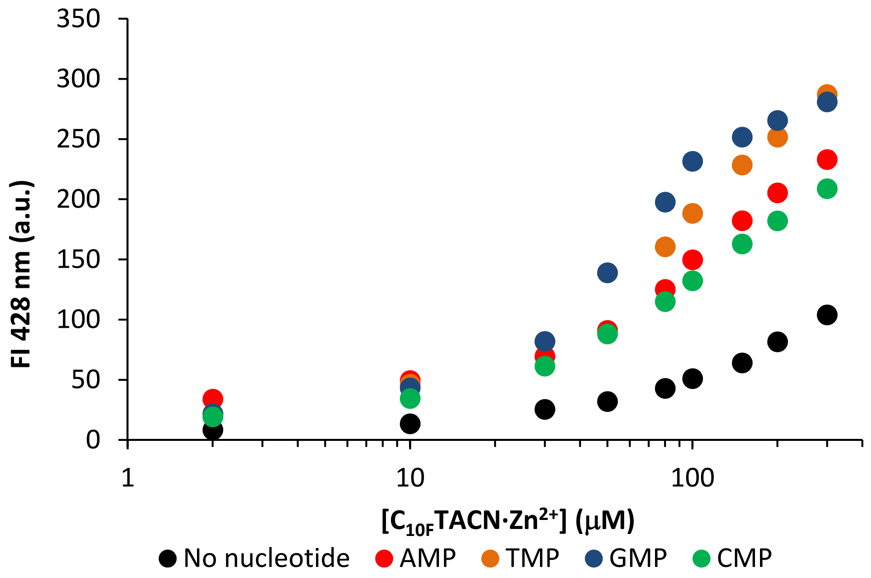

Figure S2. Fluorescence intensity at 428 nm as a function of the amount of **C_{10F}TACN·Zn^{2+}** added to an aqueous buffer solution (HEPES 5 mM, pH 7.0) in the presence of a fixed concentration of NMP (30 μM; N = G, T, A, or C) and DPH (2.5 μM) as the fluorescent probe.

Dissipative studies

The templated self-assembly under dissipative conditions of **C_{10F}TACN·Zn^{2+}** was studied by measuring the fluorescence intensity at 428 nm as a function of time after the addition (at t= 5 min) of nucleotide monophosphate (*N*MP, *N*= A, T, G, C, 50 μM) to a solution containing **C_{10F}TACN·Zn^{2+}** (50 μM) DPH (2.5 μM) and the enzyme alkaline phosphatase (1 U/ml) For a description of the used stock solutions see section 1. Under the experimental conditions (see caption to Figure S24) only for GMP a transient increase in fluorescence intensity is observed indicating the transient formation of self-assembled structures.

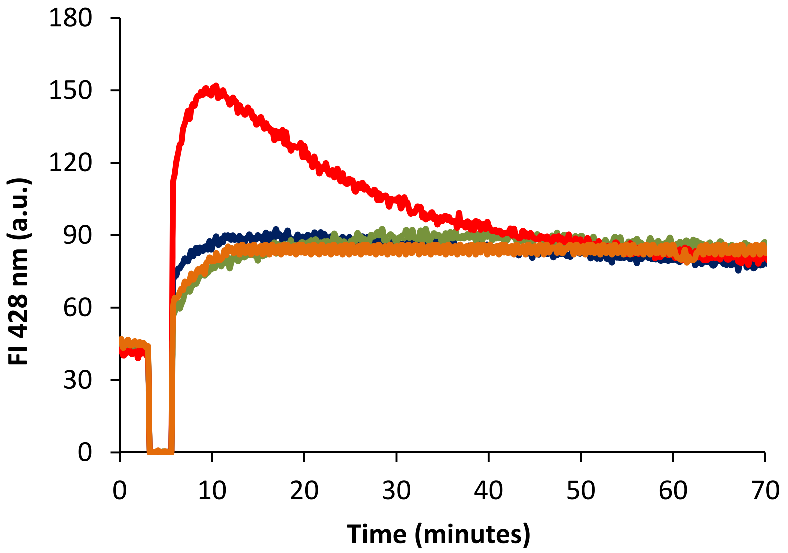

Figure S3. Fluorescence intensity at 428 nm after the additions of NMP (50 μM; N=A, T, G, C) to a solution of **C_{10F}TACN·Zn^{2+}** (50 μM) and DPH (2.5 μM) in the presence of alkaline phosphatase (AP, 1 U/mL), 5 mM HEPES pH 7.0, 25°C. (AMP blue, TMP green, GMP red and CMP orange curves).

To further prove that the products of *N*MP hydrolysis (nucleoside and phosphate) are not able to template the amphiphile's self-assembly, the experiment was repeated but a 1:1 mixture of inorganic phosphate and nucleoside (A, T, G, C) at 50 µM each was added instead of the monophosphate nucleotide.

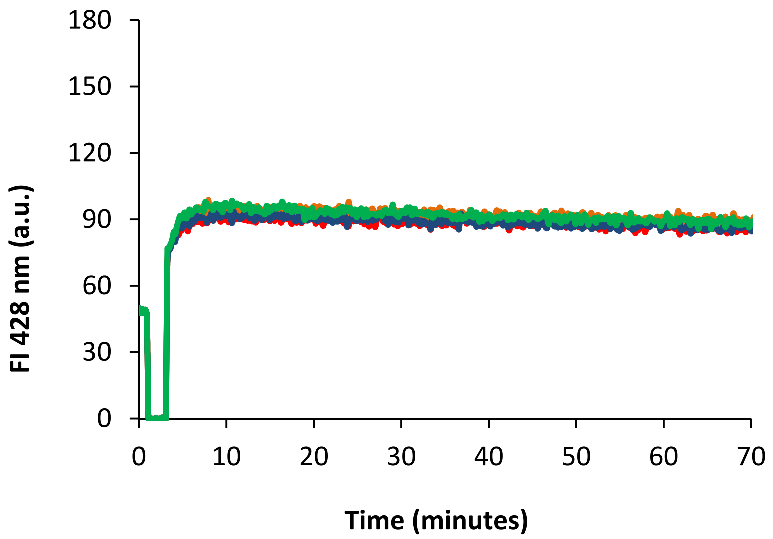

Figure S4. Fluorescence intensity at 428 nm after the additions of nucleoside (50 µM; N=A, T, G, C) and phosphate (50 µM) to a solution of **C_{10F}TACN·Zn^{2+}** (50 µM) and DPH (2.5 µM) in the presence of alkaline phosphatase (AP, 1 U/mL), 5 mM HEPES pH 7.0, 25°C. (A blue, T green, G red and C orange curves).

2.1.2 *TEM of C_{16}Cyclen·Zn^{2+} TMP*

TEM images were recorded on a Jeol 300 PX electron microscope. A drop of freshly prepared sample solution was placed on a grid for 1 min and then a drop of uranyl acetate (2 %) solution was added for 30 s. Then the solvent in excess was carefully removed with a clean tissue before imaging of the stained grid.

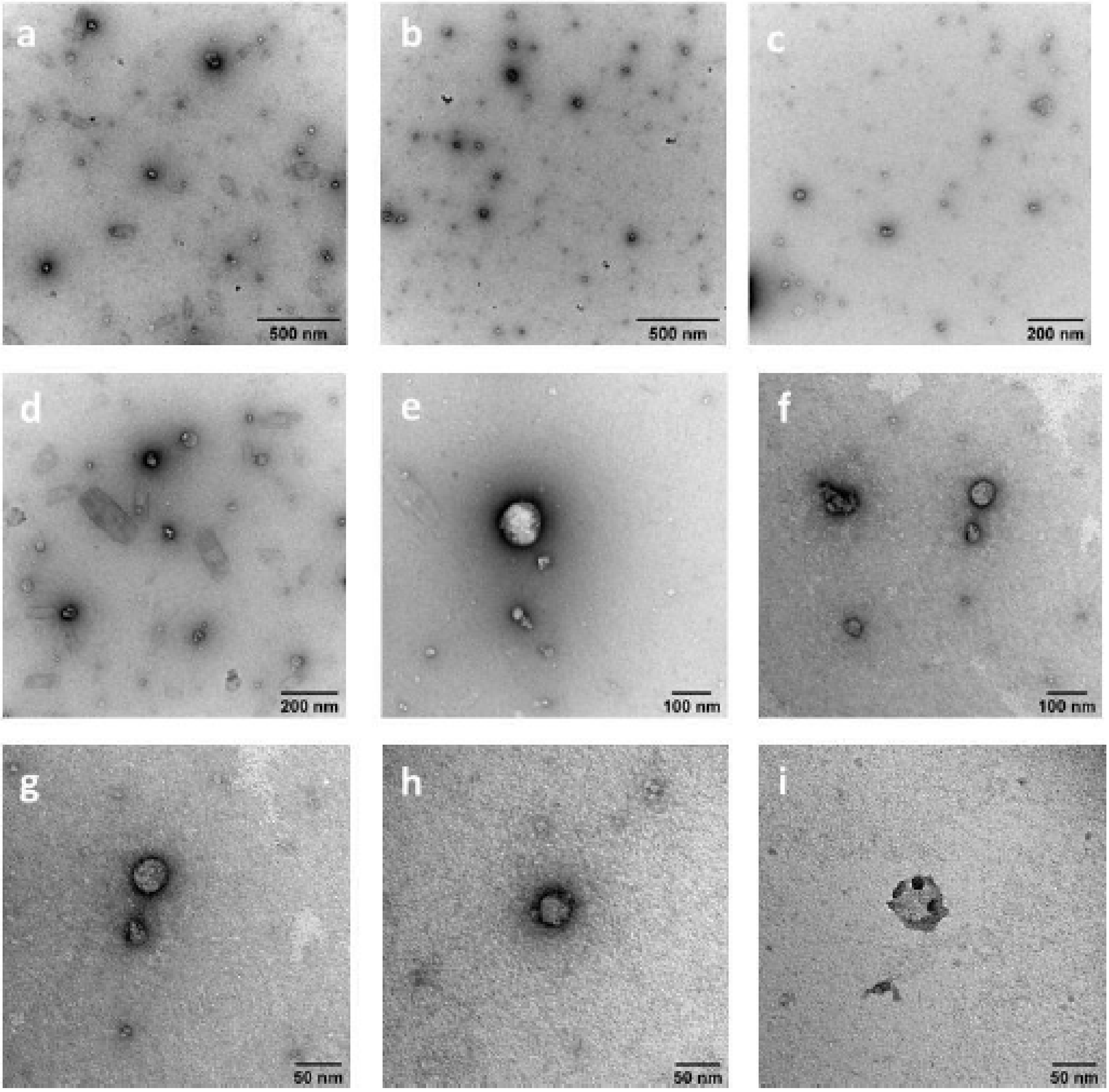

Figure S5. TEM images of a solution containing **C_{16}cyclen·Zn^{2+}** (30 µM) and TMP (30 µM). Staining was performed using 2% uranyl acetate in all cases. Experimental conditions: [HEPES] = 5 mM, pH 7.0.

2.1.3 TEM of $C_{10F}TACN \cdot Zn^{2+}$ GMP

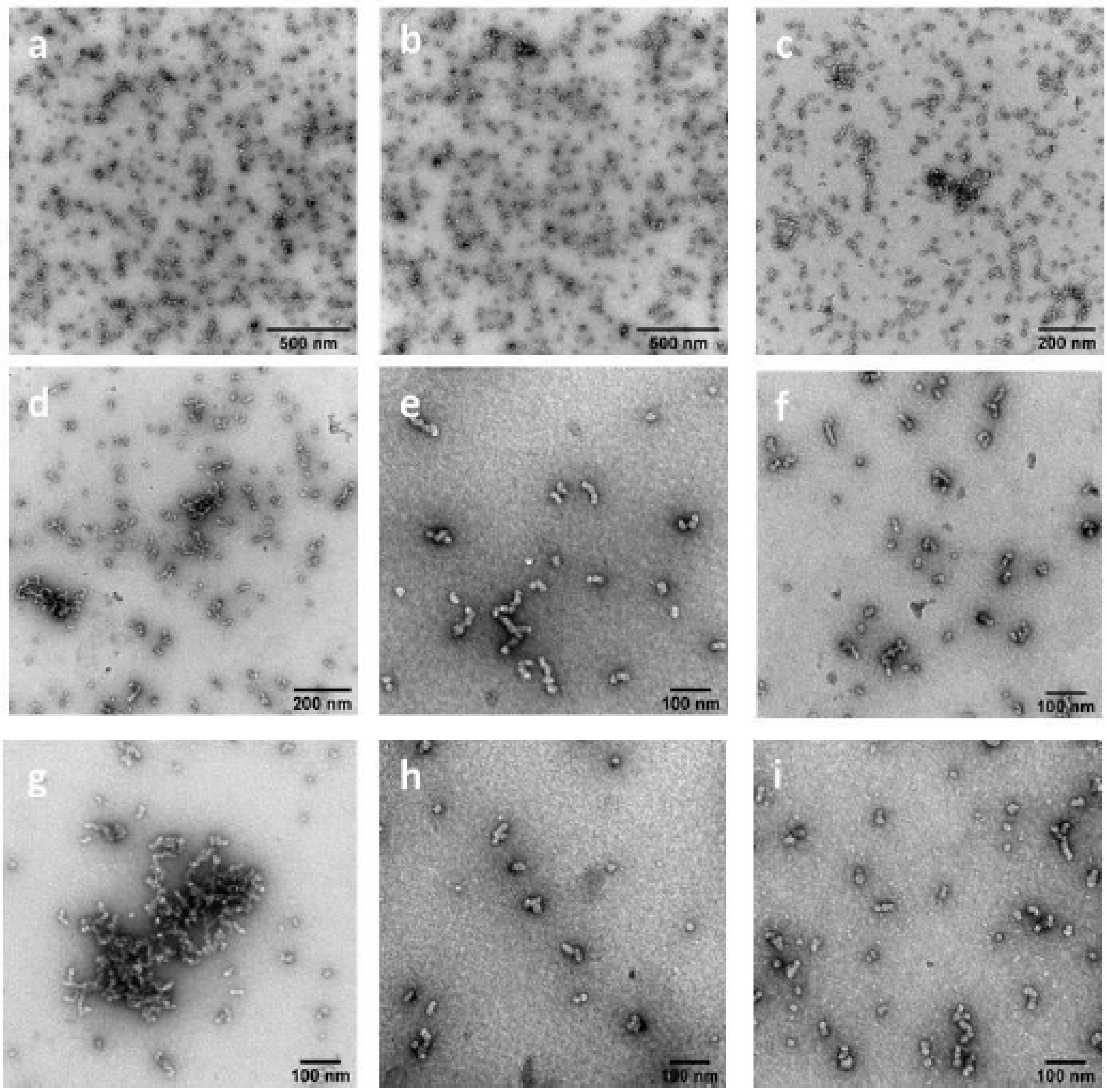

Figure S6. TEM images of a solution containing $\mathbf{C_{10F}TACN \cdot Zn^{2+}}$ (50 µM) and GMP (50 µM). Staining was performed using 2% uranyl acetate in all cases. Experimental conditions: [HEPES] = 5 mM, pH 7.0.

2.2 Mixed systems

2.2.1 TEM of $C_{10F}TACN{\cdot}Zn^{2+}$ + $C_{16}Cyclen{\cdot}Zn^{2+}$ + TMP

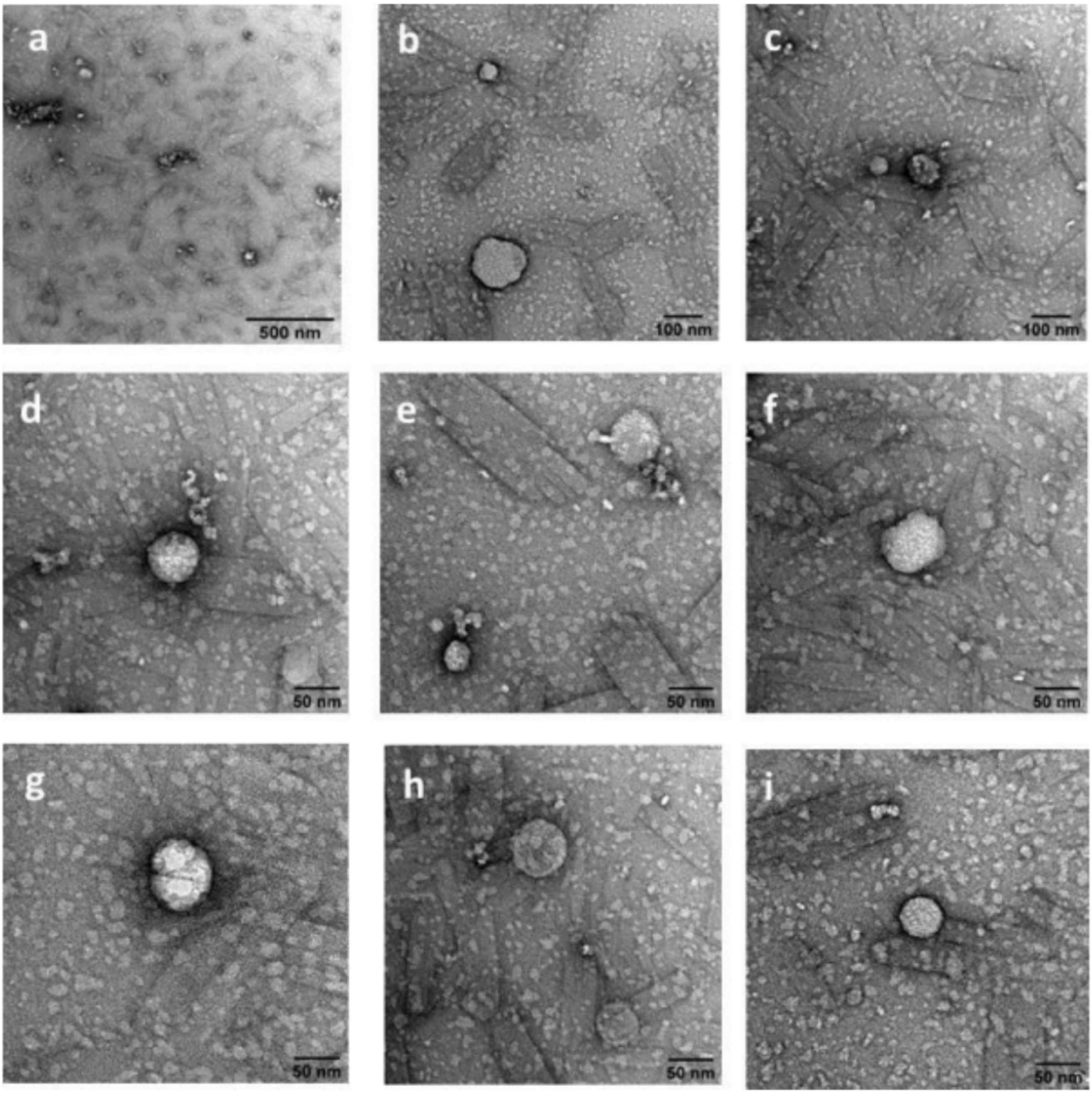

Figure S7. TEM images of a solution containing a mixture of surfactants (**C_{16}cyclen·Zn^{2+}** 30 μM and **C_{10F}TACN·Zn^{2+}** 50 μM) and TMP 50 μM. Staining was performed using 2% uranyl acetate in all cases. Experimental conditions: [HEPES]= 5 mM, pH 7.0.

2.2.2 TEM of $C_{10F}TACN\cdot Zn^{2+}$ + $C_{16}Cyclen\cdot Zn^{2+}$ + GMP

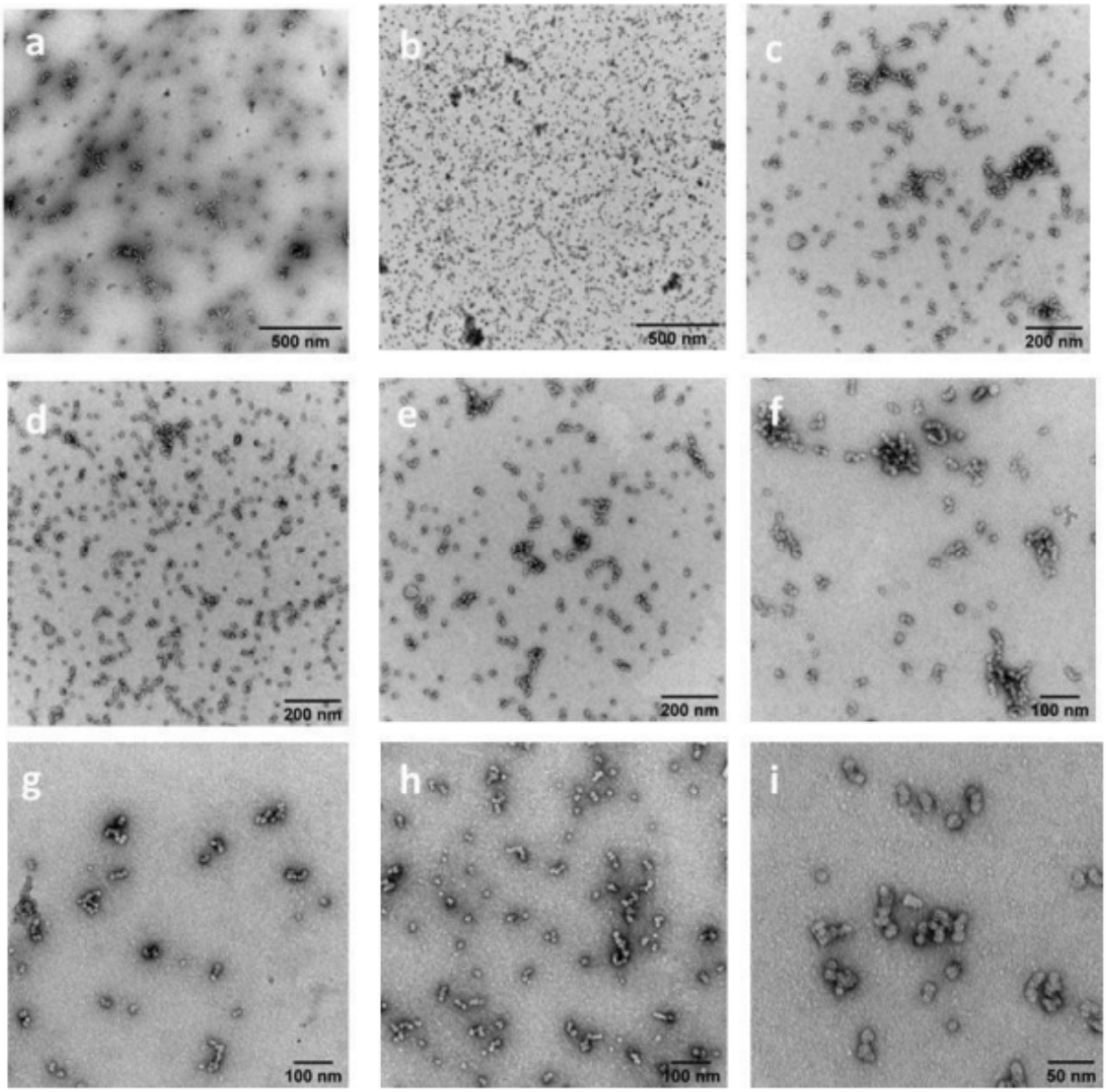

Figure S8. TEM images of a solution containing a mixture of surfactants (**C_{16}cyclen·Zn^{2+}** 30 μM and **C_{10F}TACN·Zn^{2+}** 50 μM) and GMP 50 μM. Staining was performed using 2% uranyl acetate in all cases. Experimental conditions: [HEPES]= 5 mM, pH 7.0.

2.2.3 TEM of C_{10F}TACN·Zn^{2+} + C_{16}Cyclen·Zn^{2+} + GMP + TMP

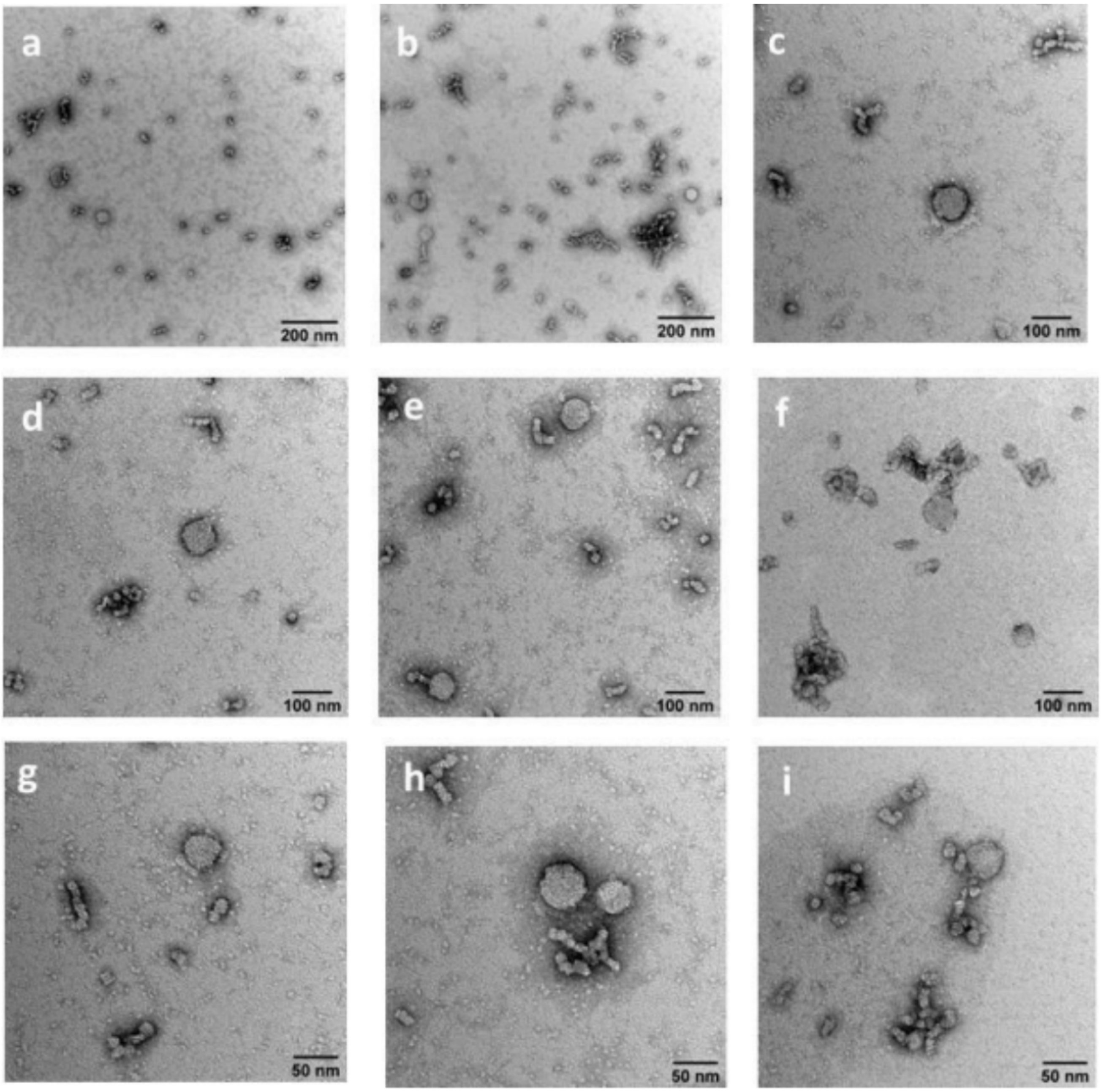

Figure S9. TEM images of a solution containing a mixture of surfactants (**C_{16}cyclen·Zn^{2+}** 30 μM and **C_{10F}TACN·Zn^{2+}** 50 μM) and 50 μM of both nucleotides (GMP and TMP). Here TMP was added 20 minutes after the addition of GMP to the system. Staining was performed using 2% uranyl acetate in all cases. Experimental conditions: [HEPES]= 5 mM, pH 7.0.

2.2.4 TEM of C_{10F}TACN·Zn^{2+} + C_{16}Cyclen·Zn^{2+} + TMP + GMP

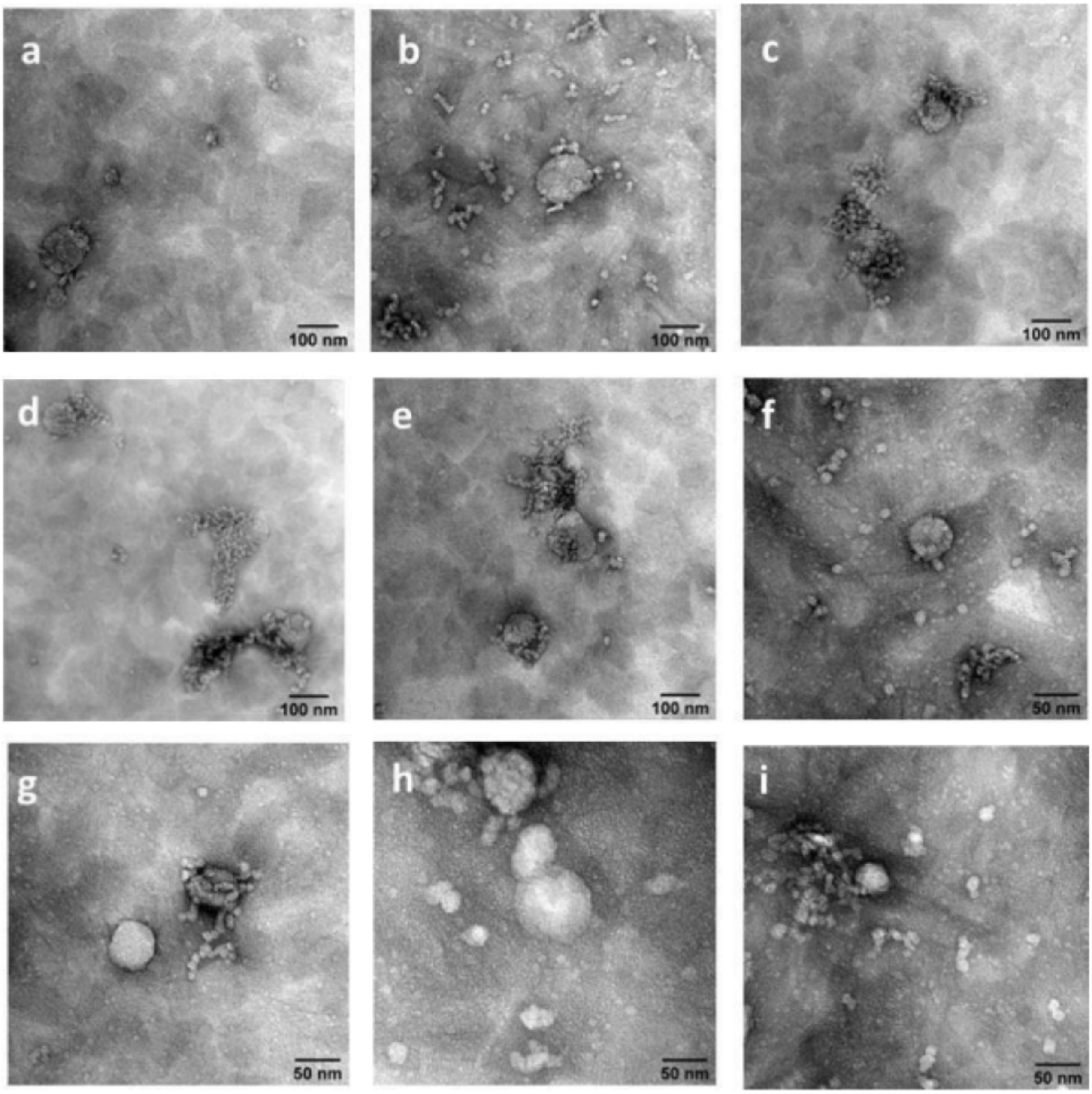

Figure S10. TEM images of a solution containing a mixture of surfactants (**C_{16}cyclen·Zn^{2+}** 30 μM and **C_{10F}TACN·Zn^{2+}** 50 μM) and 50 μM of both nucleotides (GMP and TMP). Here, GMP was added 20 minutes after the addition of TMP to the system. Staining was performed using 2% uranyl acetate in all cases. Experimental conditions: [HEPES]= 5 mM, pH 7.0.

3. UHPLC chromatograms

3.1 Materials and general methods

Surfactants stock solutions were prepared in milli-Q water and they were sonicated in a closed vial for 30 minutes before use. Thiols stock solutions containing both the thiols at the desired fluorinated/hydrogenated thiol ratio were prepared using degassed HPLC grade MeOH. NBD-Cl stock solutions (1 mM range) were prepared in anhydrous ACN. The pH of a 100 mM HEPES buffer solution was adjusted to 7.0 with a pH-meter, at room temperature.
Nucleoside monophosphate stock solutions were prepared in milli-Q water and their concentrations were verified through UV-Vis spectroscopy (ε_{268}(TMP) = 9600 $M^{-1}cm^{-1}$, ε_{253}(GMP) = 13700 M^{-1} cm^{-1}). UHPLC eluents were: milli-Q water + 0.1% HPLC grade formic acid as solvent A, and HPLC grade ACN + 0.1% HPLC grade formic acid as solvent B. UHPLC column: ZORBAX SB-C3 column (Rapid Resolution HT 2.0 × 100 mm 1.8-Micron).
All stock solutions and UHPLC eluents were conserved at 4°C, except for thiol, nucleoside and nucleotide monophosphate stock solutions, which were conserved at -20°C.

Operative conditions for typical UHPLC experiments:

The appropriate amount of milli-Q H_2O to have a final volume of 1 mL was added into a 2 mL mass vial. Then the required system components were added in the following order:

- HEPES 5 mM pH = 7.0
- Zn^{2+} 600 µM
- **C_{10F}TACN** 50 µM and/or **C_{16}Cyclen** 30 µM
- C_{6F}SH 4.5 µM + C_{4H}SH 1.5 µM
- alkaline phosphatase (AP, 1 U/mL)
- NBD-Cl 2 µM
- GMP 50 µM or TMP 30 µM

Immediately after the *N*MP addition the vial was closed and shaken for 10 seconds, then it was injected in the UHPLC.

<u>UHPLC method</u>

Flow = 0.300 mL/min, Column temperature T = 50°C

- From 50% to 62.2% of B in 6.5 min.
- From 62.2% to 85% of B in 5 min.
- From 85% to 99% of B in 1.5 min.
- After 0.1 min the flow increases up to 0.600 mL/min
- After 3.9 min from 99% to 50% of B in 0.1 min
- After 3.4 min flow returns to 0.300 mL/min in 0.1 min. The method finishes after 0.5 min.

3.2 Chromatograms

3.2.1. Reference chromatograms for C_{4H}S-NBD and C_{6F}S-NBD

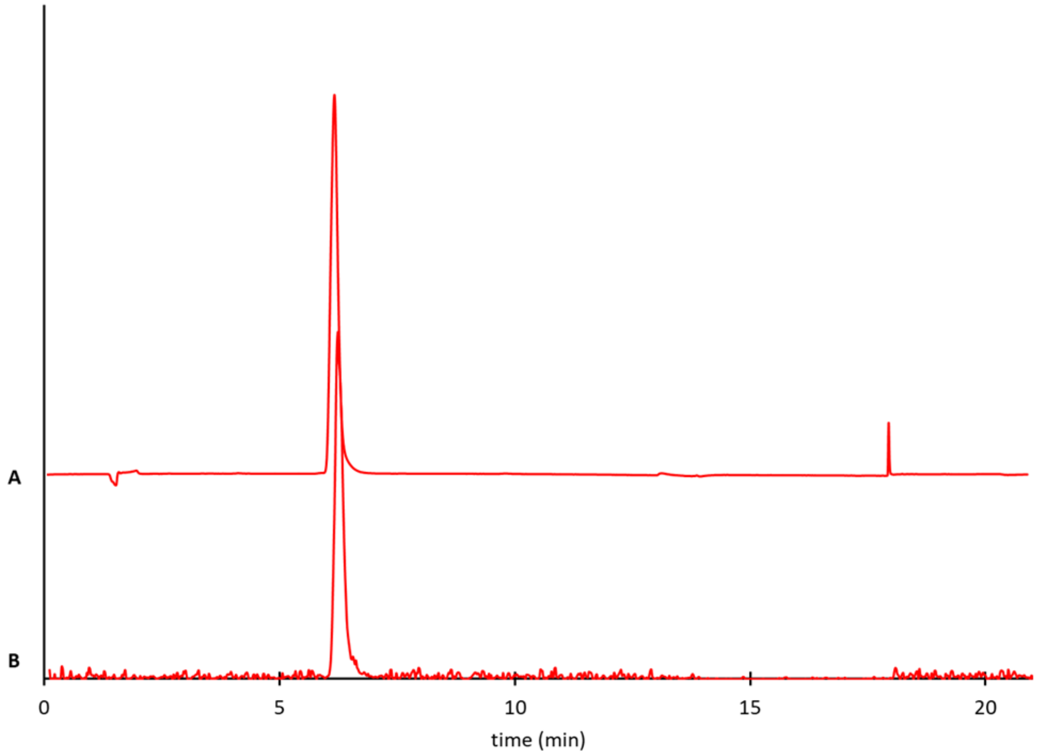

Figure S11. Chromatograms of **C_{4H}S-NBD** 4 µM standard solution obtained with (**A**) UV-Vis detector (416 nm) and (**B**) ESI-MS ($[M+H]^+$=254.1).

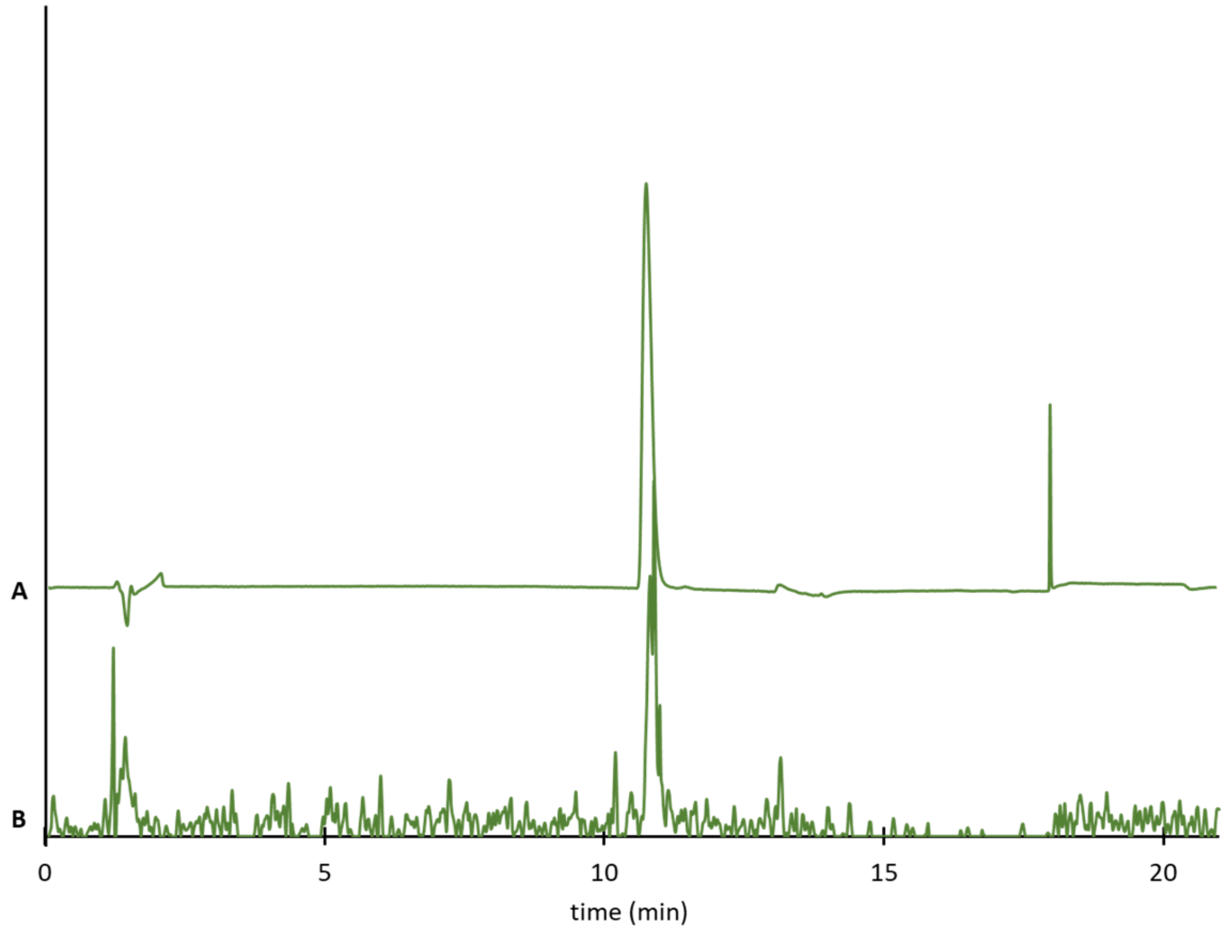

Figure S12. Chromatograms of **C_{6F}S-NBD** 4 µM standard solution obtained with (**A**) UV-Vis detector (416 nm) and (**B**) ESI-MS ($[M+H]^+$=542.5).

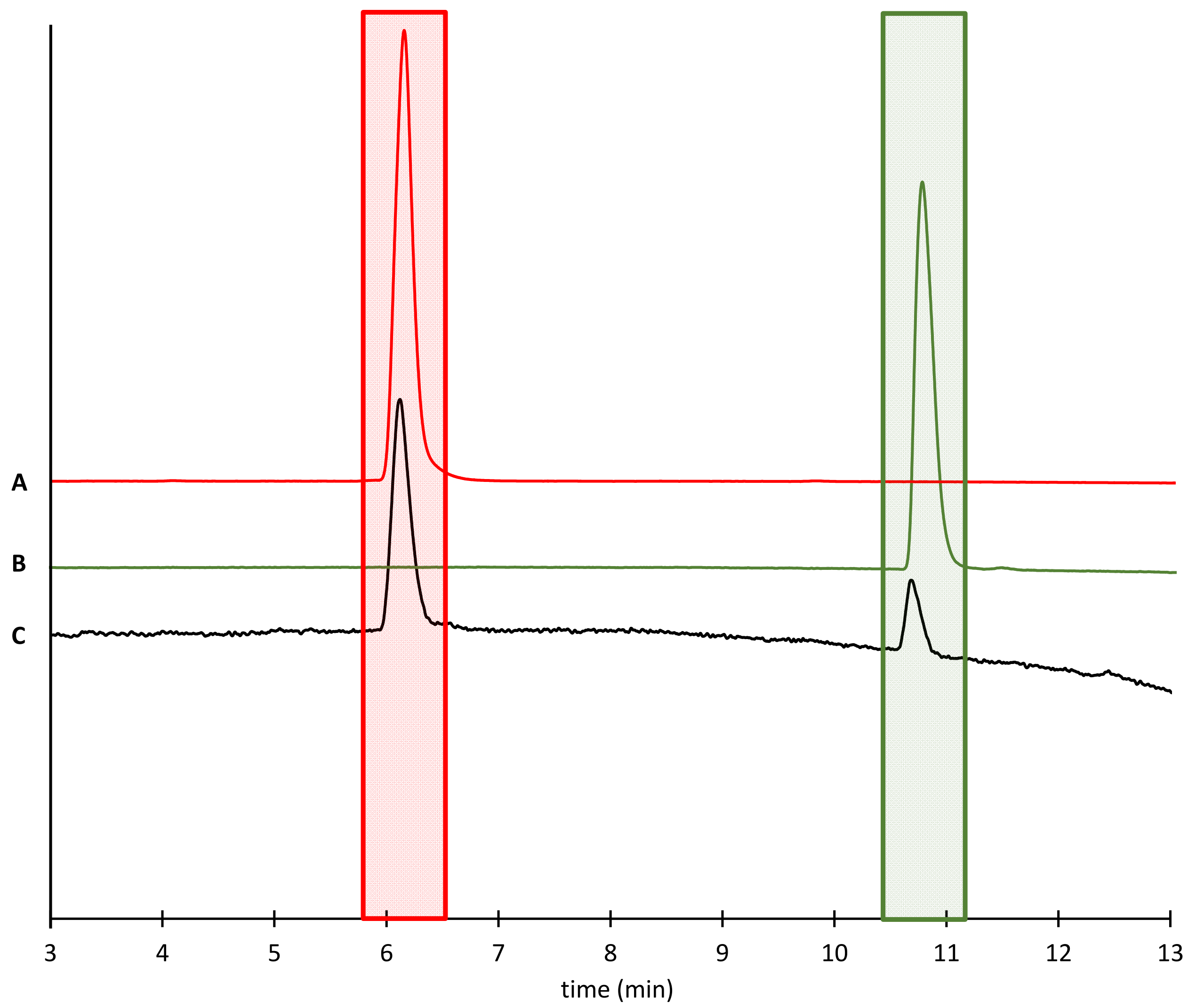

Figure S13. Comparison between (**A**) **C_{4H}S-NBD** 4 µM standard solution, (**B**) **C_{6F}S-NBD** 4 µM standard solution and (**C**) reaction mixture (HEPES 5 mM pH = 7.0, Zn^{2+} 600 µM, **C_{10F}TACN** 50 µM, **C_{4H}SH** 4.5 µM, **C_{6F}SH** 1.5 µM, NBD-Cl 2 µM and GMP 50 µM) after 45 min of reaction.

3.2.2. Calibration curves

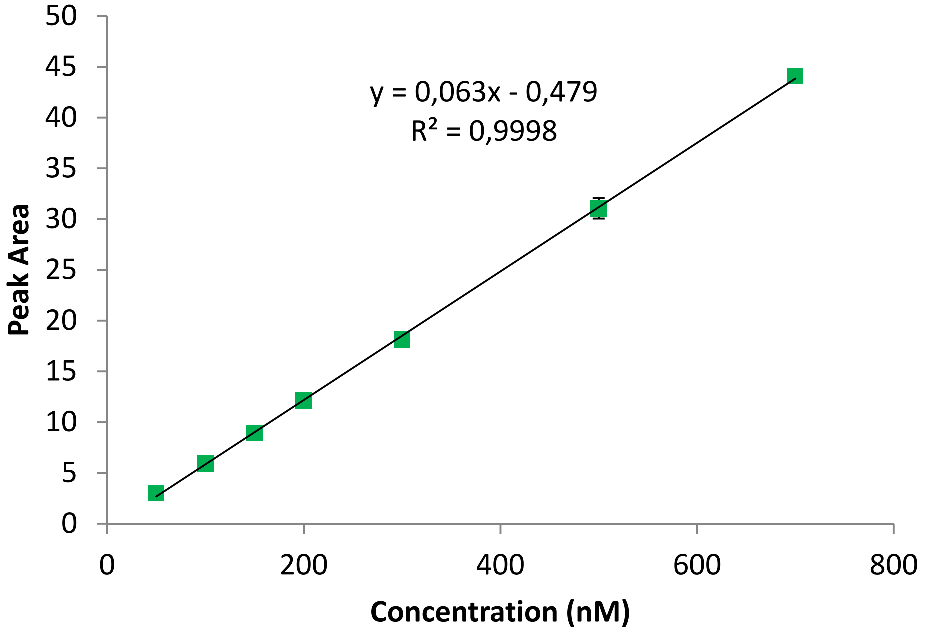

Figure S14. Calibration plot prepared by plotting the HPLC peak area of **C_{6F}S-NBD** as a function of the **C_{6F}S-NBD** concentration. Experimental conditions: HEPES = 5mM, pH 7.0; λ_{det} = 416 nm, 20 µL injection volume. For most of the points the error bars (indicating the standard errors) are smaller than the square indicators (experiments repeated in duplicate).

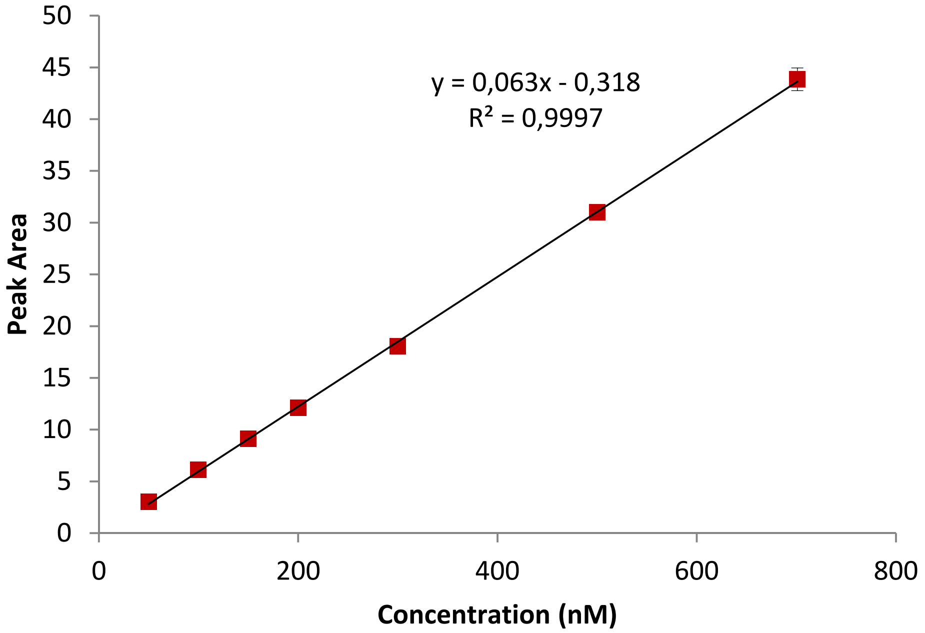

Figure S15. Calibration plot prepared by plotting the HPLC peak area of **C_{4H}S-NBD** as a function of the **C_{4H}S-NBD** concentration. Experimental conditions: HEPES = 5mM, pH 7.0; λ_{det} = 416 nm, 20 µL injection volume. For most of the points the error bars (indicating the standard errors) are smaller than the square indicators (experiments repeated in duplicate).

3.2.3. Chromatograms for the screening study reported in Figure 4b and 4c

We report here the representative chromatograms used for the preliminar thiol screening shown in Figure **4b** and **4c** of the manuscript. The preliminary studies using thiols **C_{12}SH**, **C_8SH**, **C_6SH** and **tBuSH** have been performed with the following UHPLC method, which is slithly different from that one optimised for thiol **C_4SH** (which was used for all the other experiments).

<u>UHPLC method</u> for **C_{12}SH**, **C_8SH**, **C_6SH** and **tBuSH** thiols:

Flow = 0.300 mL/min, Column temperature T = 50°C

- From 50% to 81% of B in 7 min.
- From 81% to 88% of B in 1.2 min.
- From 88% to 99% of B in 0.05 min.
- After 0.05 min the flow increases up to 0.600 mL/min
- After 2.7 min from 99% to 50% of B in 0.1 min
- After 3.5 min flow returns to 0.300 mL/min in 0.1 min. The method finishes after 0.5 min.

C_{6F}SH/C_{12H}SH

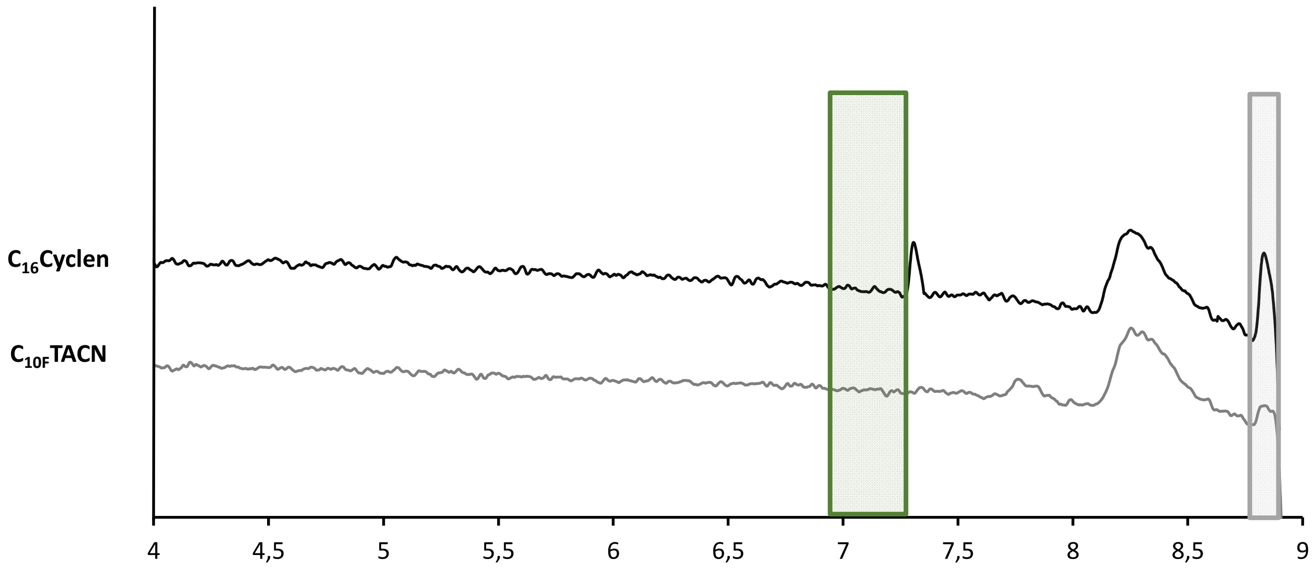

Figure S16. Chromatogram comparison of 20 minutes reaction mixtures composed of **C_{12H}SH** 2 µM, **C_{6F}SH** 2 µM and NBD-Cl 2 µM, in the presence of **C_{16}cyclen** 30 µM and TMP 30 µM (top) or **C_{10F}TACN** 50 µM and GMP 50 µM (bottom). HEPES 5 mM pH = 7.0, Zn^{2+} 600 µM. **C_{12H}S-NBD** is highlighted in grey, **C_{6F}S-NBD** in green.

C_{6F}SH/C_{8H}SH

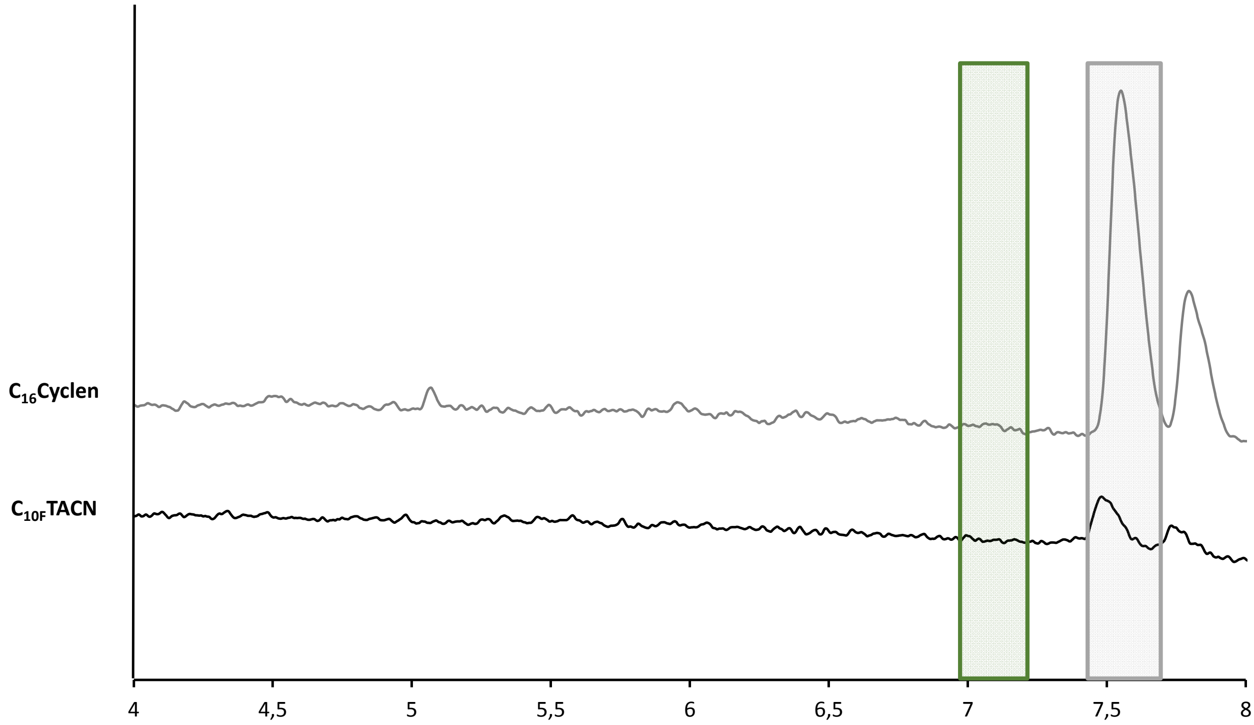

Figure S17. Chromatogram comparison of 20 minutes reaction mixtures composed of **C_{8H}SH** 2 µM, **C_{6F}SH** 2 µM and NBD-Cl 2 µM, in the presence of **C_{16}cyclen** 30 µM and TMP 30 µM (top) or **C_{10F}TACN** 50 µM and GMP 50 µM (bottom). HEPES 5 mM pH = 7.0, Zn^{2+} 600 µM. **C_{8H}S-NBD** is highlighted in grey, **C_{6F}S-NBD** in green.

C_{6F}SH/C_{6H}SH

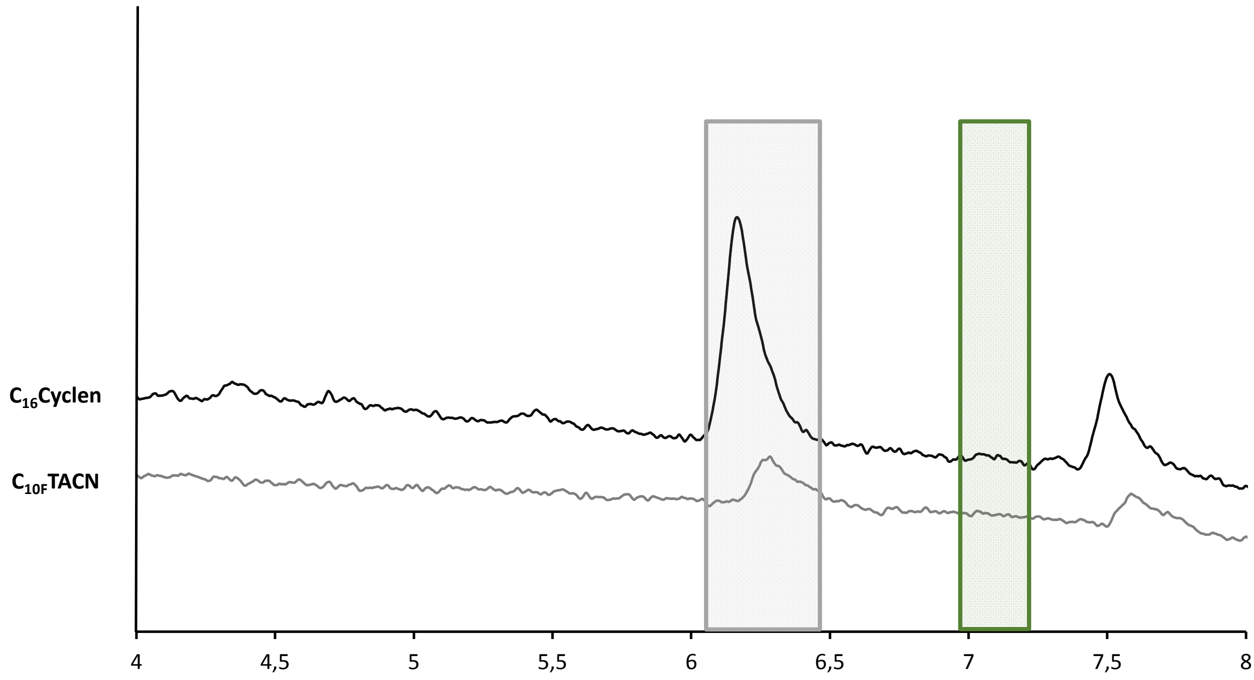

Figure S18. Chromatogram comparison of 20 minutes reaction mixtures composed of **C_{6H}SH** 2 µM, **C_{6F}SH** 2 µM and NBD-Cl 2 µM, in the presence of **C_{16}cyclen** 30 µM and TMP 30 µM (top) or **C_{10F}TACN** 50 µM and GMP 50 µM (bottom). HEPES 5 mM pH = 7.0, Zn^{2+} 600 µM. **C_{6H}S-NBD** is highlighted in grey, **C_{6F}S-NBD** in green.

C_{6F}SH/C_{tBuH}SH

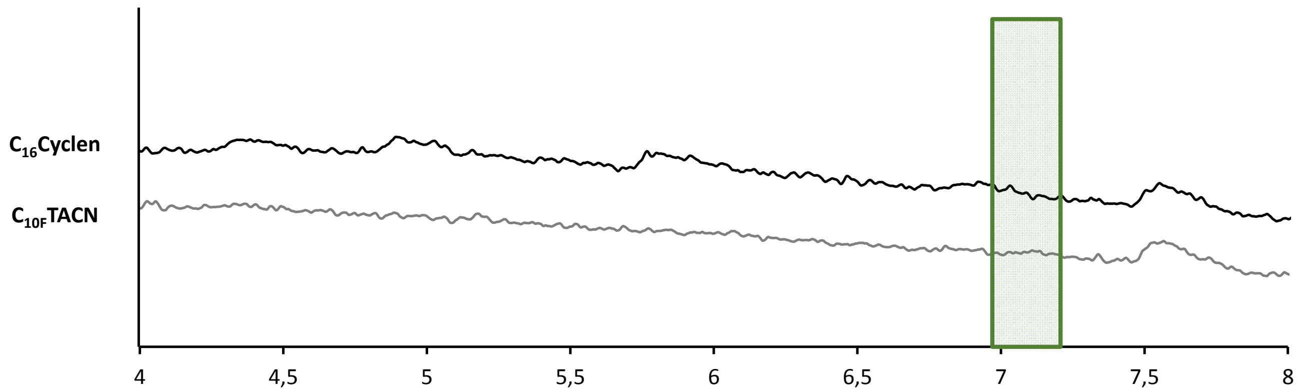

Figure S19. Chromatogram comparison of 20 minutes reaction mixtures composed of **C_{tBuH}SH** 2 µM, **C_{6F}SH** 2 µM and NBD-Cl 2 µM, in the presence of **C_{16}cyclen** 30 µM and TMP 30 µM (top) or **C_{10F}TACN** 50 µM and GMP 50 µM (bottom). HEPES 5 mM pH = 7.0, Zn^{2+} 600 µM. **C_{6F}S-NBD** is highlighted in green, while **C_{tBuH}S-NBD** was not formed even in acetonitrile because of its poor reactivity.

$C_{6F}SH/C_{4H}SH$

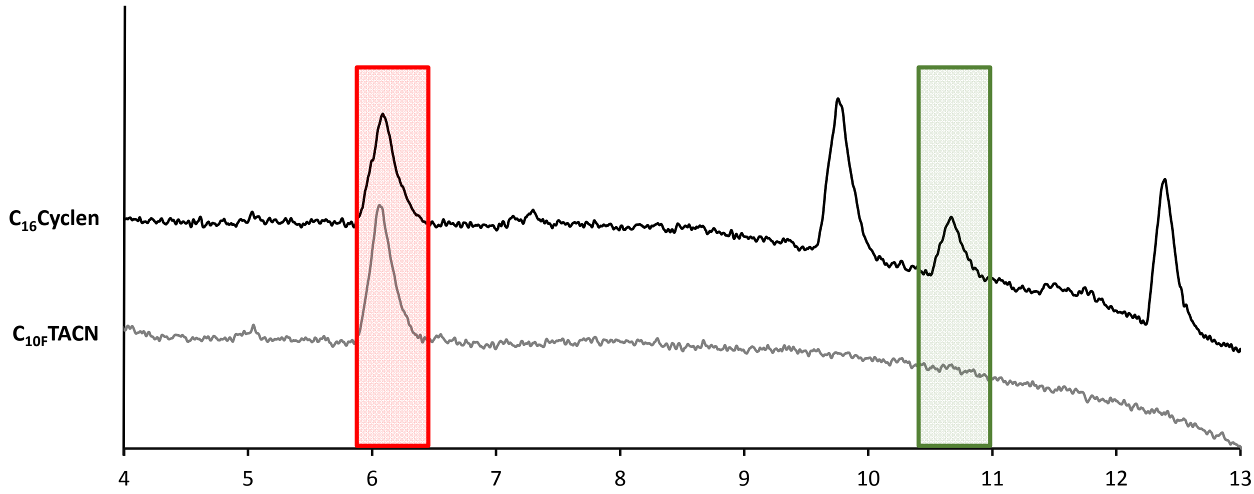

Figure S20. Chromatogram comparison of 20 minutes reaction mixtures composed of **$C_{4H}SH$** 2 µM, **$C_{6F}SH$** 2 µM and NBD-Cl 2 µM, in the presence of **C_{16}cyclen** 30 µM and TMP 30 µM (top) or **C_{10F}TACN** 50 µM and GMP 50 µM (bottom). HEPES 5 mM pH = 7.0, Zn^{2+} 600 µM. **C_{4H}S-NBD** is highlighted in red, **C_{6F}S-NBD** in green. These chromatograms were obtained using the optimised method reported at page S31.

3.2.4. Chromatograms used for Figure 4d and 4e

Representative chromatograms used for Figure **4d** in the manuscript. The experiment was carried out in duplicate.

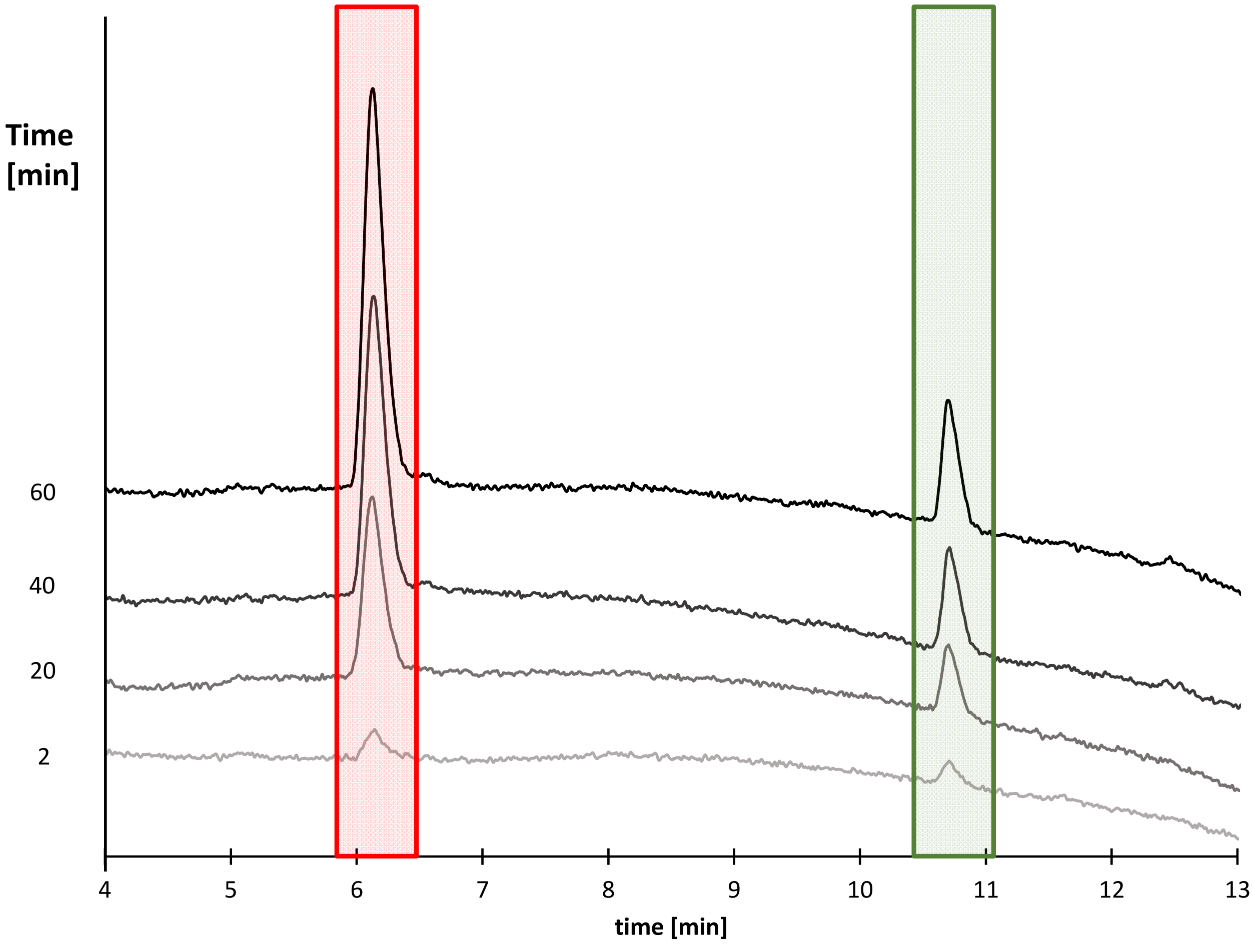

Figure S21. Product formation as a function of time in the reaction mixture composed of $\mathbf{C_{4H}SH}$ 4.5 µM, $\mathbf{C_{6F}SH}$ 1.5 µM and NBD-Cl 2 µM, in the presence of $\mathbf{C_{10F}TACN}$ 50 µM and GMP 50 µM. HEPES 5 mM pH = 7.0, Zn^{2+} 600 µM, experiment repeated in duplicate. $\mathbf{C_{4H}S}$**-NBD** is highlighted in red, $\mathbf{C_{6F}S}$**-NBD** in green.

Representative chromatograms used for Figure **4e** in the manuscript. The experiment was carried out in duplicate.

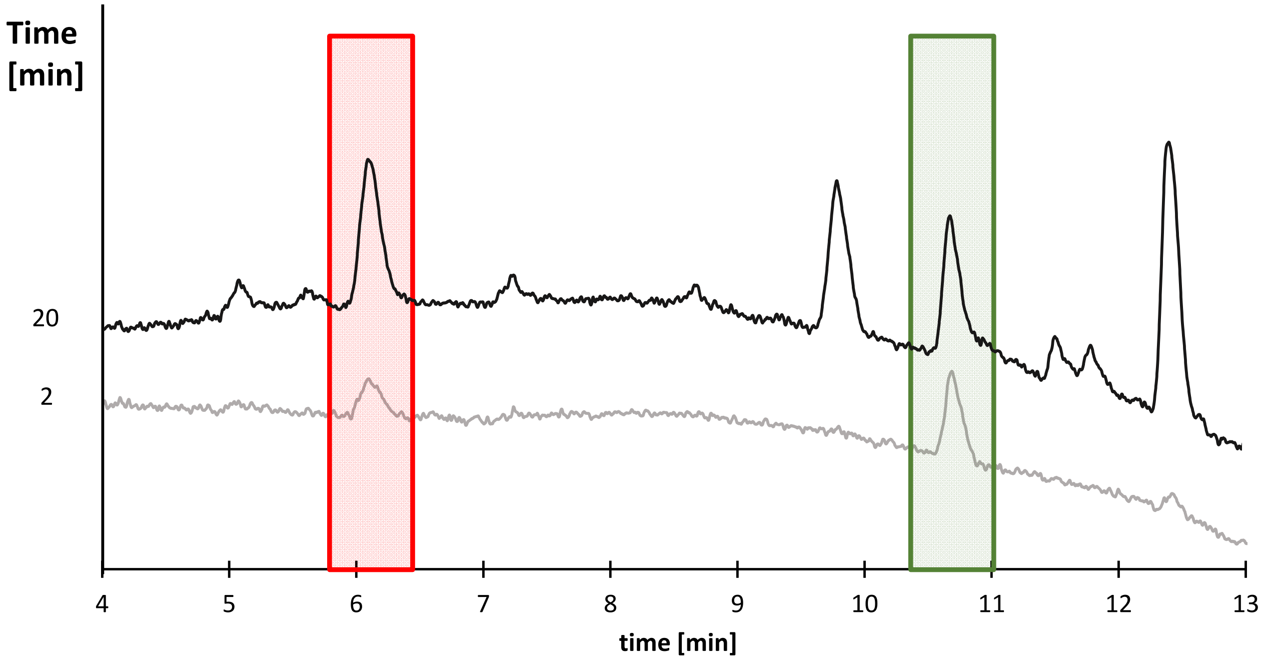

Figure S22. Product formation as a function of time in the reaction mixture composed of **C_{4H}SH** 4.5 µM, **C_{6F}SH** 1.5 µM and NBD-Cl 2 µM, in the presence of **C_{16}cyclen** 30 µM, and TMP 30 µM. HEPES 5 mM pH = 7.0, Zn^{2+} 600 µM, experiment repeated in duplicate. After 20 minutes of reaction two main impurities become relevant with retention times of 9.8 and 12.5 minutes, respectively. These impurities have been previously observed[1] and MS analysis suggested that these are **C_{16}cyclen** functionalized with respectively 2 ($[M+H]^+$: found: 723.4, calcd: 723.4) and 3 NBD moieties ($[M+Zn]^{2+}$: found: 474.6, calcd: 474.7). A more detailed discussion on side products is given on page S46. **C_{4H}S-NBD** is highlighted in red, **C_{6F}S-NBD** in green.

3.2.5. Control experiments in the absence of nucleotide

Control experiment for the isolated surfactant **C_{10F}TACN** in the absence of nucleotide

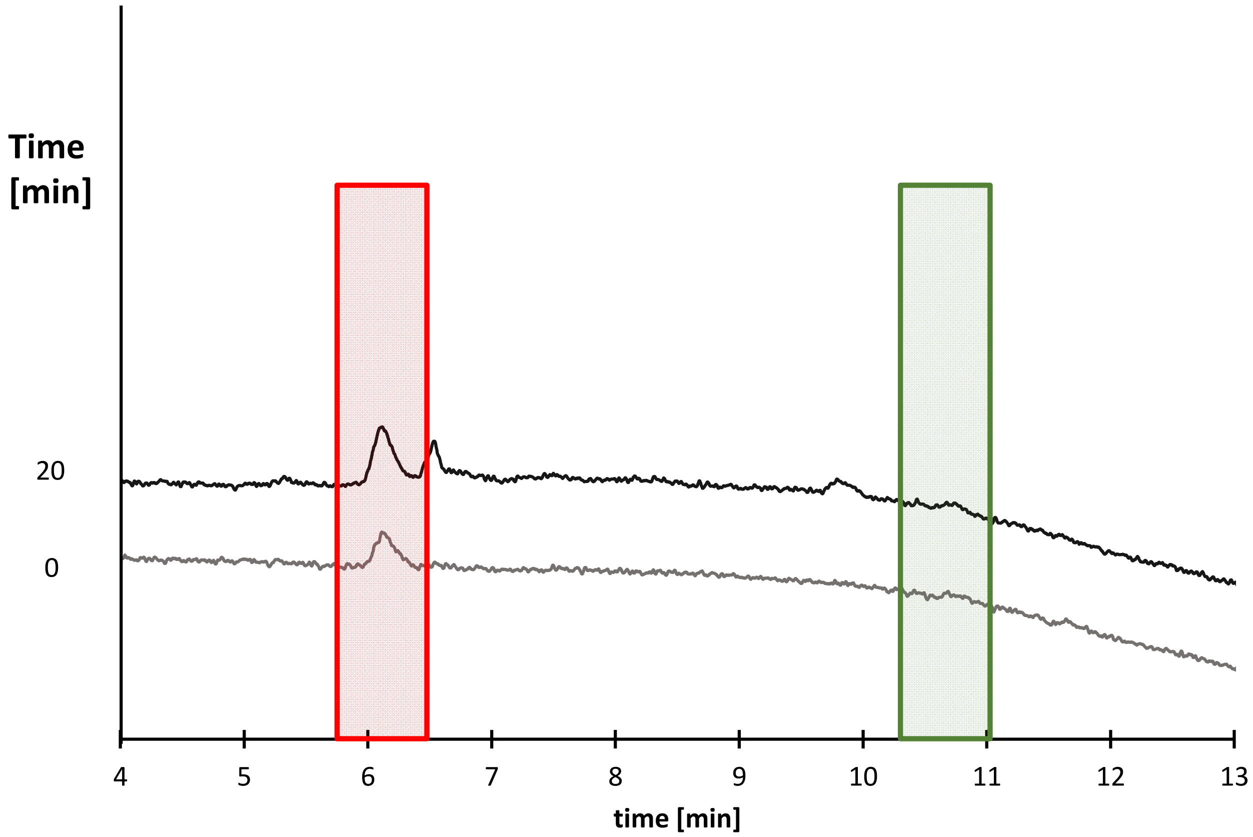

Figure S23. Product formation as a function of time in the reaction mixture composed **C_{4H}SH** 4.5 µM, **C_{6F}SH** 1.5 µM and NBD-Cl 2 µM, in the presence of **C_{10F}TACN** 50 µM but in the absence of any NMPs. HEPES 5 mM pH = 7.0, Zn^{2+} 600 µM, experiment repeated in duplicate. **C_{4H}S-NBD** is highlighted in red, and its concentration is 18.8 nM and 28.1 nM respectively after 2 min and 20 min of reaction. **C_{6F}S-NBD** is highlighted in green, and it is 0 nM and 2.9 nM respectively after 2 min and 20 min of reaction.

Control experiment for the isolated surfactant **C_{16}cyclen** in the absence of nucleotide

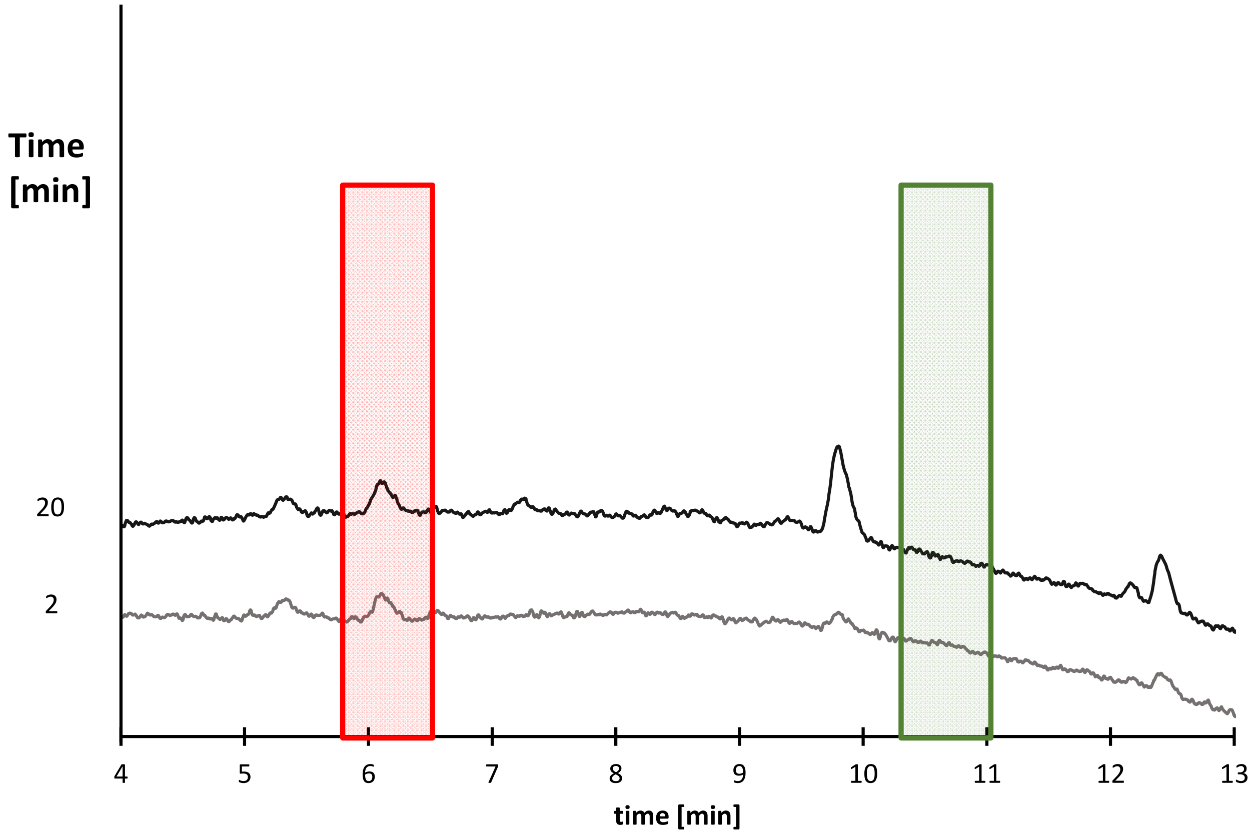

Figure S24. Product formation as a function of time in the reaction mixture composed **C_{4H}SH** 4.5 µM, **C_{6F}SH** 1.5 µM and NBD-Cl 2 µM, in the presence of **C_{16}cyclen** 30 µM but in the absence of any NMPs. HEPES 5 mM pH = 7.0, Zn^{2+} 600 µM, experiment repeated in duplicate. **C_{4H}S-NBD** is highlighted in red, and its concentration is 8.3 nM and 12.7 nM respectively after 2 min and 20 min of reaction. **C_{6F}S-NBD** is highlighted in green, and it is absent in the observed conditions.

3.2.6. Chromatograms used for Figure 6

Representative chromatograms used for the control experiment shown in Figure **6a** in the manuscript. The experiment was carried out in duplicate.

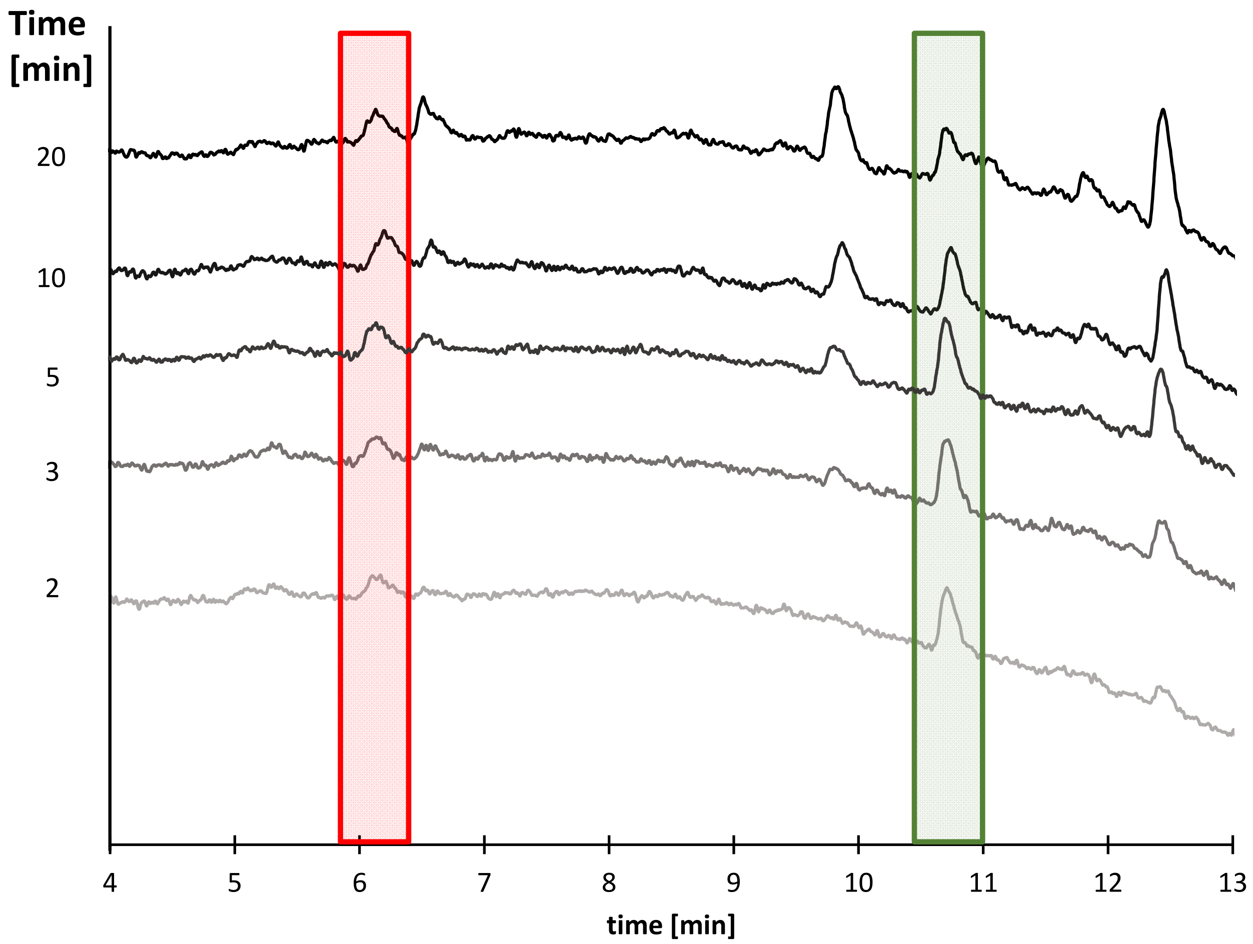

Figure S25. Product formation as a function of time in the reaction mixture composed **C_{4H}SH** 4.5 µM, **C_{6F}SH** 1.5 µM and NBD-Cl 2 µM, in the presence of **C_{16}cyclen** 30 µM, **C_{10F}TACN** 50 µM but in the absence of any *N*MPs. HEPES 5 mM pH = 7.0, Zn^{2+} 600 µM, experiment repeated in duplicate. **C_{4H}S-NBD** is highlighted in red, **C_{6F}S-NBD** in green. For a discussion on the additional peaks see page S46.

Representative chromatograms used for Figure **6b** in the manuscript. The experiment was carried out in triplicate.

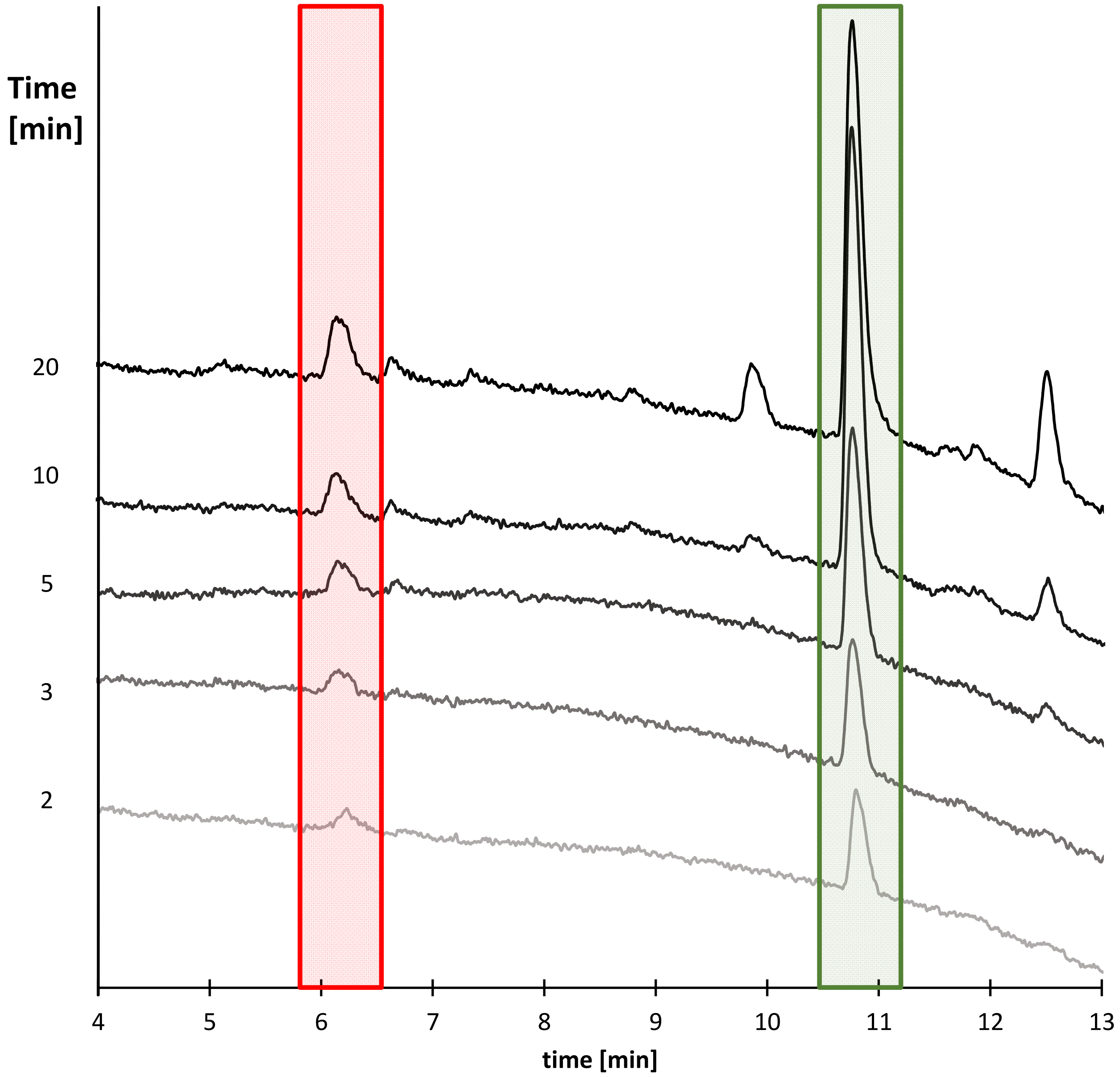

Figure S26. Product formation as a function of time in the reaction mixture composed of **C_{4H}SH** 4.5 µM, **C_{6F}SH** 1.5 µM and NBD-Cl 2 µM, in the presence of **C_{16}cyclen** 30 µM, **C_{10F}TACN** 50 µM, and TMP 30 µM. HEPES 5 mM pH = 7.0, Zn^{2+} 600 µM, experiment repeated in triplicate. **C_{4H}S-NBD** is highlighted in red, **C_{6F}S-NBD** in green. For a discussion on the additional peaks see page S46.

Representative chromatograms used for Figure **6c** in the manuscript. The experiment was carried out in triplicate.

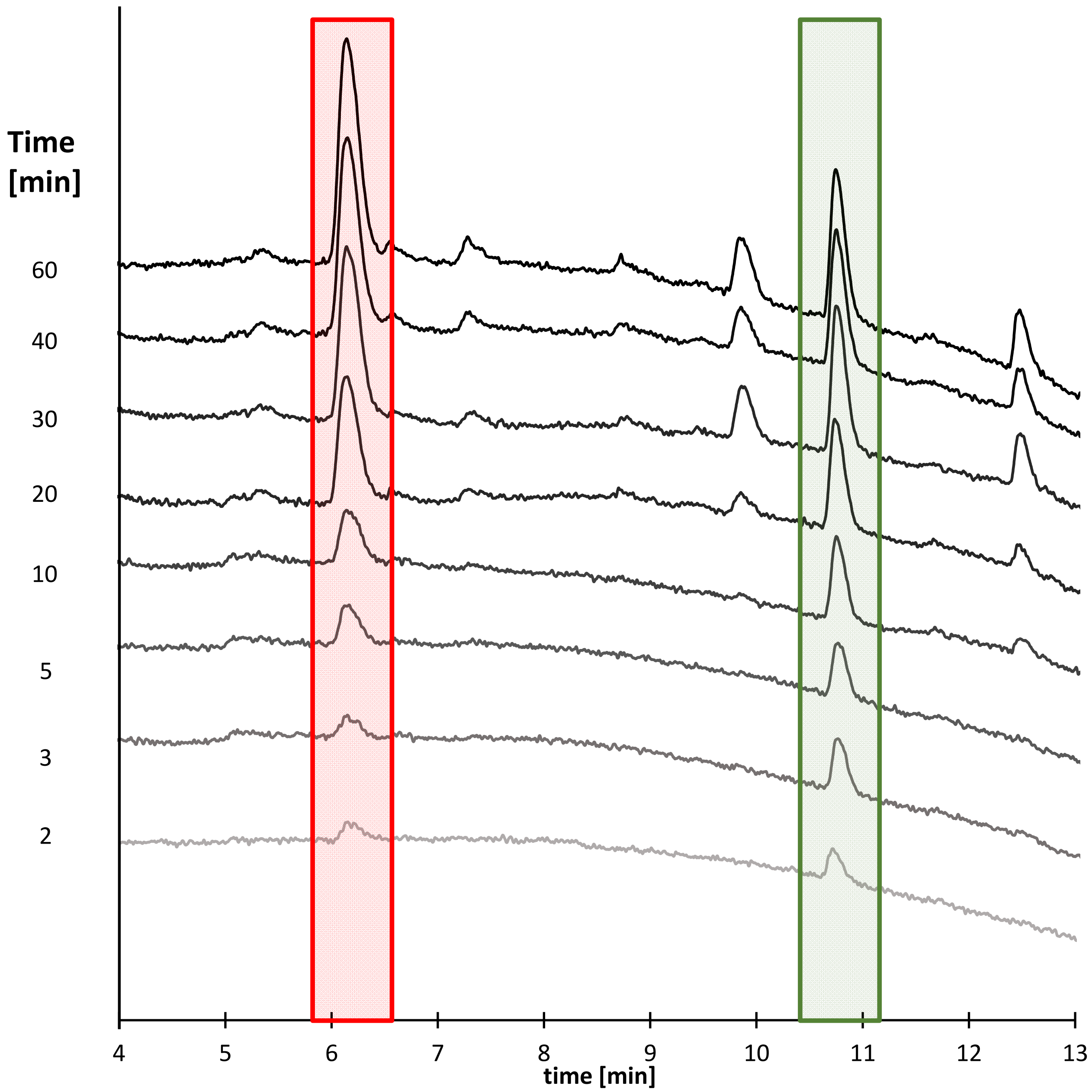

Figure S27. Product formation as a function of time in the reaction mixture composed of **C_{4H}SH** 4.5 µM, **C_{6F}SH** 1.5 µM and NBD-Cl 2 µM, in the presence of **C_{16}Cyclen** 30 µM, **C_{10F}TACN** 50 µM, and GMP 50 µM. HEPES 5 mM pH = 7.0, Zn^{2+} 600 µM, experiment repeated in triplicate. **C_{4H}S-NBD** is highlighted in red, **C_{6F}S-NBD** in green. For a discussion on the additional peaks see page S46.

Representative chromatograms used for Figures **6d** and **6e** in the manuscript. The experiment was carried out in duplicate.

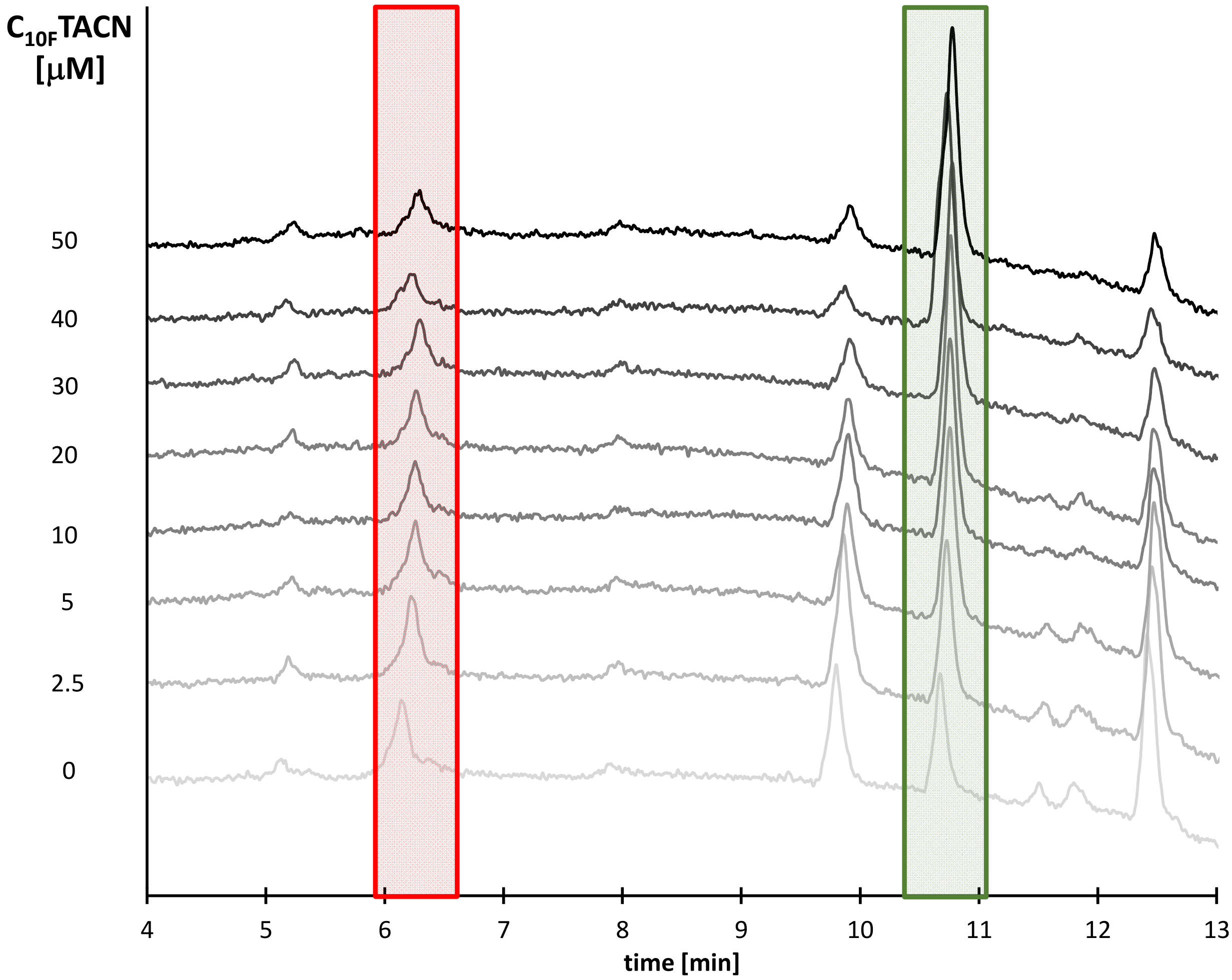

Figure S28. Chromatograms for 20 min reaction of mixtures composed of **C_{4H}SH** 4.5 μM, **C_{6F}SH** 1.5 μM and NBD-Cl 2 μM, in the presence of **C_{16}Cyclen** 30 μM, TMP 30 μM and containing increasing amount of **C_{10F}TACN** (from 0 μM to 50 μM). HEPES 5 mM pH = 7.0, Zn^{2+} 600 μM, experiment repeated in duplicate. **C_{4H}S-NBD** is highlighted in red, **C_{6F}S-NBD** in green. For a discussion on the additional peaks see page S46.

3.2.7. Chromatograms used for Figure 7

Representative chromatograms used for the experiment shown in Figure **7** in the manuscript in which just GMP is added at t=0. The experiment was carried out in quadruplicate. Changes in the composition of two samples were monitored for 134 minutes (Figure S29), whereas to other two samples TMP was added after t = 50 minutes (Figure S30).

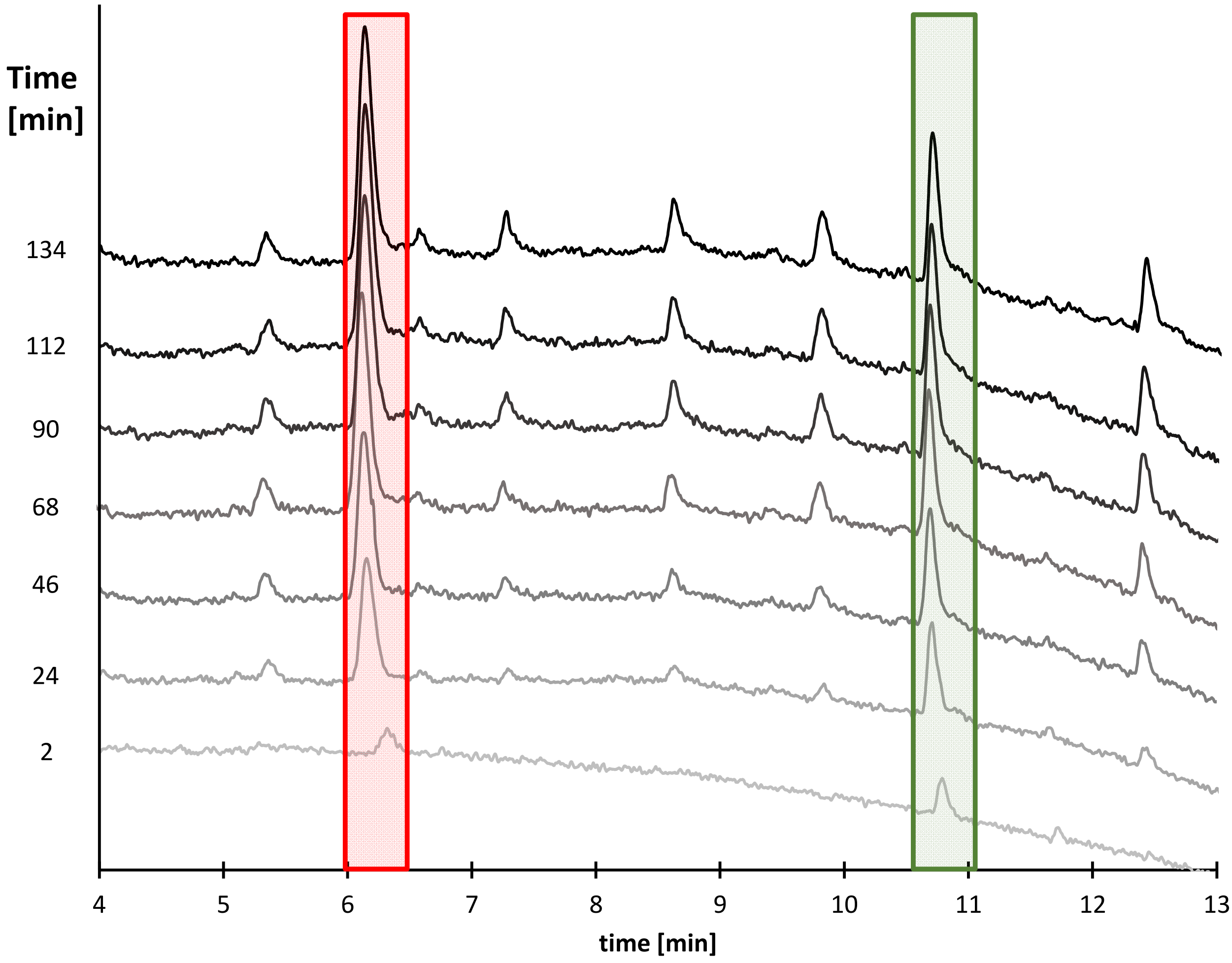

Figure S29. Product formation as a function of time in the reaction mixture composed of **C_{4H}SH** 4.5 µM, **C_{6F}SH** 1.5 µM and NBD-Cl 2 µM, in the presence of **C_{16}cyclen** 30 µM, **C_{10F}TACN** 50 µM, AP (1 u/mL) and GMP 50 µM. HEPES 5 mM pH = 7.0, Zn^{2+} 600 µM, experiment repeated in duplicate. **C_{4H}S-NBD** is highlighted in red, **C_{6F}S-NBD** in green. For a discussion on the additional peaks see page S46.

Representative chromatograms used for the experiment shown in Figure **7** in the manuscript in which GMP is added at t=0 minutes and TMP is added at t= 50 minutes.

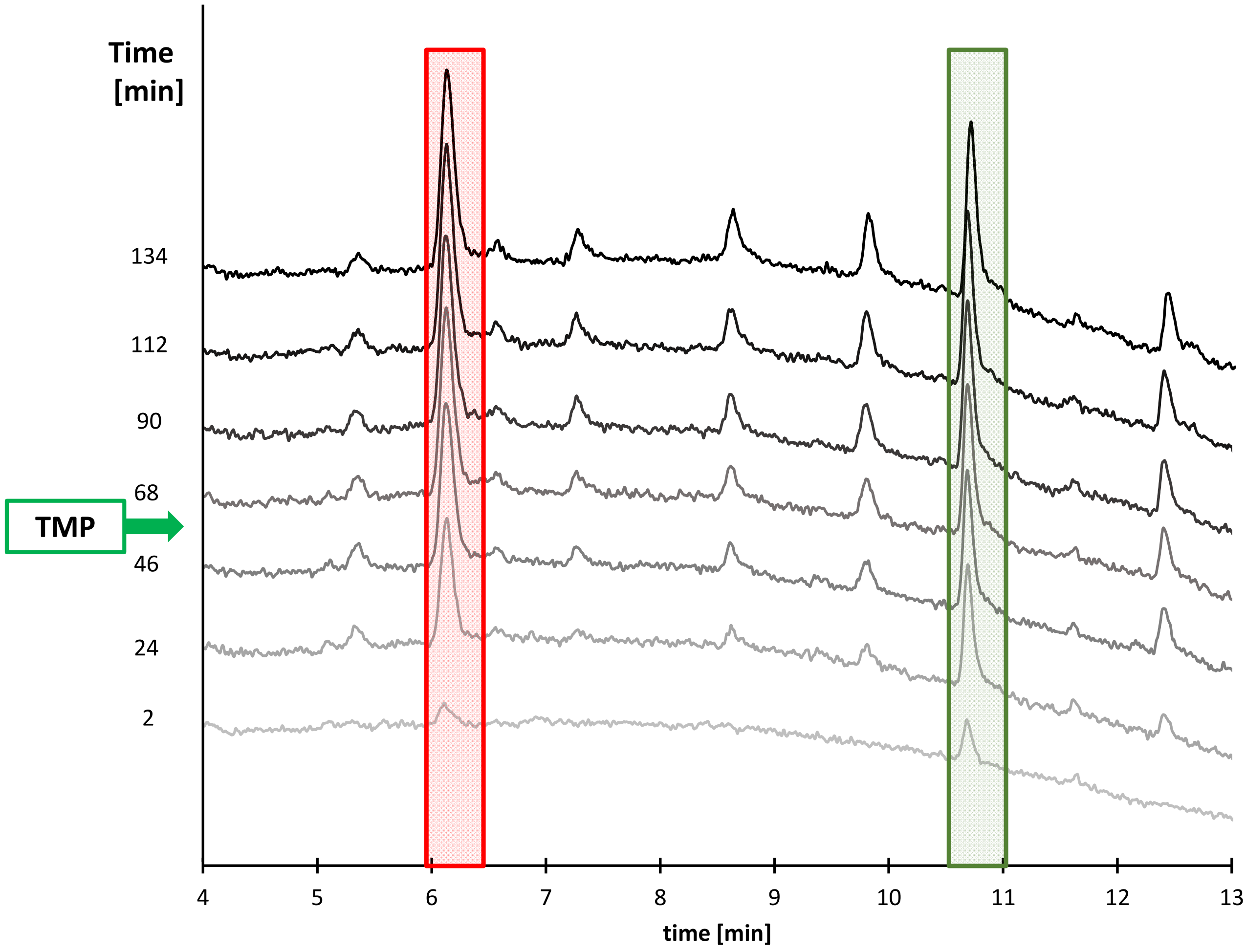

Figure S30. Product formation as a function of time in the reaction mixture composed of **C_{4H}SH** 4.5 µM, **C_{6F}SH** 1.5 µM and NBD-Cl 2 µM, in the presence of **C_{16}cyclen** 30 µM, **C_{10F}TACN** 50 µM, AP (1 u/mL) and GMP 50 µM. TMP (30 µM) was added after 50 minutes. HEPES 5 mM pH = 7.0, Zn^{2+} 600 µM, experiment repeated in duplicate. **C_{4H}S-NBD** is highlighted in red, **C_{6F}S-NBD** in green. Discussion on the formation of side products is provided in figure S48. For a discussion on the additional peaks see page S46.

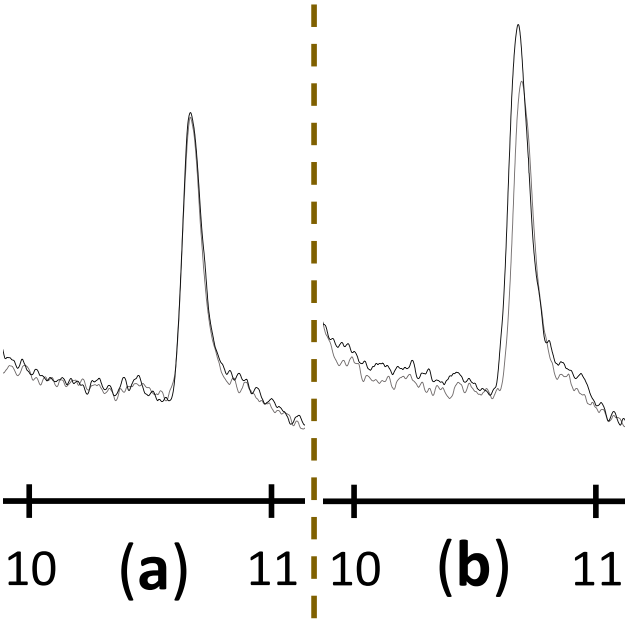

Figure S31. Comparison between **C_{6F}S-NBD** product observed after 68 min (grey) and 133 min (black) of kinetic experiments reported in figures S43 ((**a**), no TMP added) *vs* figures S44 ((**b**), TMP added at minute 50, see also Figure 2 in the manuscript).

3.2.8. Formation of side products

Many of the chromatograms shown in this section show the slow gradual increase in intensity of additional peaks not corresponding to the reaction products between **C_{4H}S-NBD** (red squares) and **C_{6F}S-NBD** (green squares). These peak appear particularly prominent when the **C_{16}cyclen·Zn^{2+}** surfactant is used.

We investigated the formation of side products that appear in the key experiment reported in Figure 2a of the manuscript (chromatograms given in Figures S43 and S44). The area of all the observed peaks is plotted as a function of time in Figure S48.

Considering the detection wavelength (416 nm) these peaks are attributed to NBD-adducts, most likely to the surfactants. Indeed, major peaks could indeed be attributed by MS-spectrometry to **C_{16}cyclen** adducts with 2 and 3 NBD-moieties (discussed in Figure S36). Because of the unknown molar extinction coefficients of side products, the peak areas have been plotted in figure S48. However, since the impurities are mostly equipped with more than one NBD unit, the peak area is likely to overestimate the real concentration of the side products.

From a kinetic point of view, the formation of the side products happens with a much slower rate compared to the formation of **C_{4H}S-NBD** (red squares) and **C_{6F}S-NBD** (green squares), indicating that their formation is unrelated to the self-assembly process of nanoreactors. Indeed, the kinetic profiles resemble those observed in the absence of nucleotide monophosphate (see Figure 7a in the manuscript).

In conclusion, although the chromatograms do not appear clean, the formation of side products does not affect the working principle of the system:

1.the concentration of the side products is very low and is likely to be even lower than suggested by the peak areas

2.the rate of formation of the side products occurs on a much slower time scale and is not affected by the self-assembly process

3.the amount of modified surfactant is insignificant compared to the total amount of surfactant present.

4.the amount of side product formed is lower in the complex system containing both **C_{16}cyclen·Zn^{2+}** and **C_{10F}TACN·Zn^{2+}** compared to the solutions containing just one of the surfactants (compare Figure S22 to Figure S26 and see the titration experiment in Figure S28).

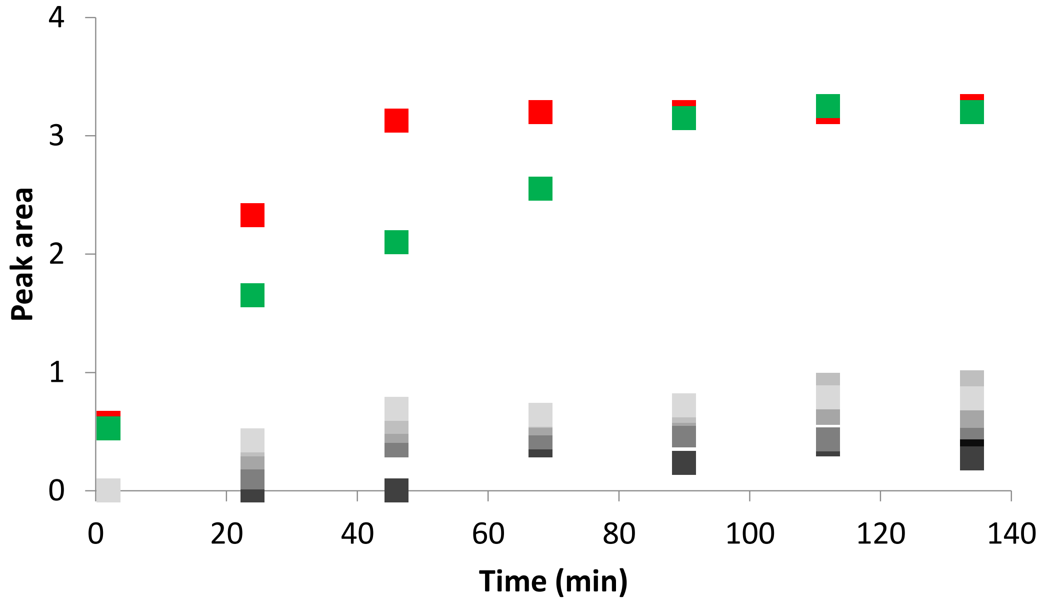

Figure S32. Peak area of **C_4HS-NBD** (red squares), **C_6FS-NBD** (green squares) and the side products with retention time of 5.3, 6.6, 7.3, 8.6, 9.8 and 12.4 min (from black to light grey squares) as a function of time for a solution containing both **C_{10F}TACN·Zn^{2+}** (50 μM) and **C_{16}cyclen·Zn^{2+}** (30 μM), **NBD-Cl** (2 μM), **C_4HSH** (1.5 μM), **C_6FSH** (4.5 μM) and AP (1 u/mL). **GMP** (50 μM) was added at t = 0 and **TMP** (30 μM) was added at t = 50 minutes. HEPES 5 mM pH = 7.0, Zn^{2+} 600 μM.

4. Synthesis and characterisation

4.1 Synthesis of C_{16}Cyclen

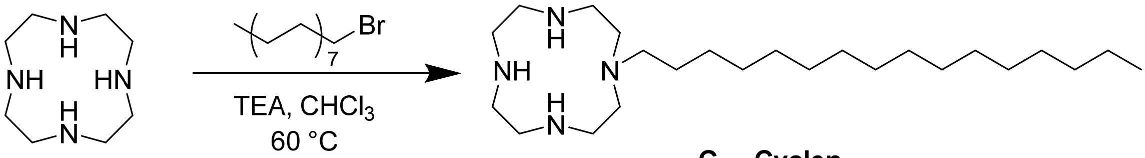

C16HCyclen

1,4,7,10-Tetraazacyclododecane (1.5 g, 8.72 mmol, 4 equiv) and triethylamine (364 µL, 2.62 mmol, 1.2 equiv) were dissolved in 22 mL of chloroform and refluxed under nitrogen atmosphere. 1-Bromohexadecane (665 mg, 2.18 mmol, 1 equiv) was then slowly added and the solution was reacted for 5 hours. The mixture was cooled to room temperature, filtered and washed with NaOH 1 M (3x)and finally with brine. The organic fractions were collected and dried with Na_2SO_4. Then the solution was filtered, the solvent was evaporated under reduced pressure and the crude product was purified through flash column chromatography (silica gel, gradient from 95:5 to 9:1 Isopropanol:NH_3 (30% aq)), giving the desired surfactant **C_{16}Cyclen** (796 mg, 92% yield).

^{1}H NMR: (δ ppm, 500 MHz, $CDCl_3$): δ 2.83 – 2.76 (m, 4H), 2.64 (t, *J* = 5.1 Hz, 4H), 2.61 – 2.57 (m, 4H), 2.57 – 2.50 (m, 4H), 2.45 – 2.38 (m, 2H), 1.53 – 1.42 (m, 2H), 1.33 – 1.23 (m, 28H), 0.89 (t, *J* = 6.7 Hz, 3H).
^{13}C NMR: (δ ppm, 300 MHz, $CDCl_3$): δ 54.6, 51.6, 31.9, 29.7, 29.4, 27.5, 27.3, 22.7, 14.1.
ESI-MS (ESI+, H_2O:CH_3CN = 1:1): $[M+H]^+$: found: 397.5; calcd: 397.4. $[M+ Na]^+$: found: 419.5; calcd: 419.4.
Spectroscopic data are in agreement with those reported in literature.[1]

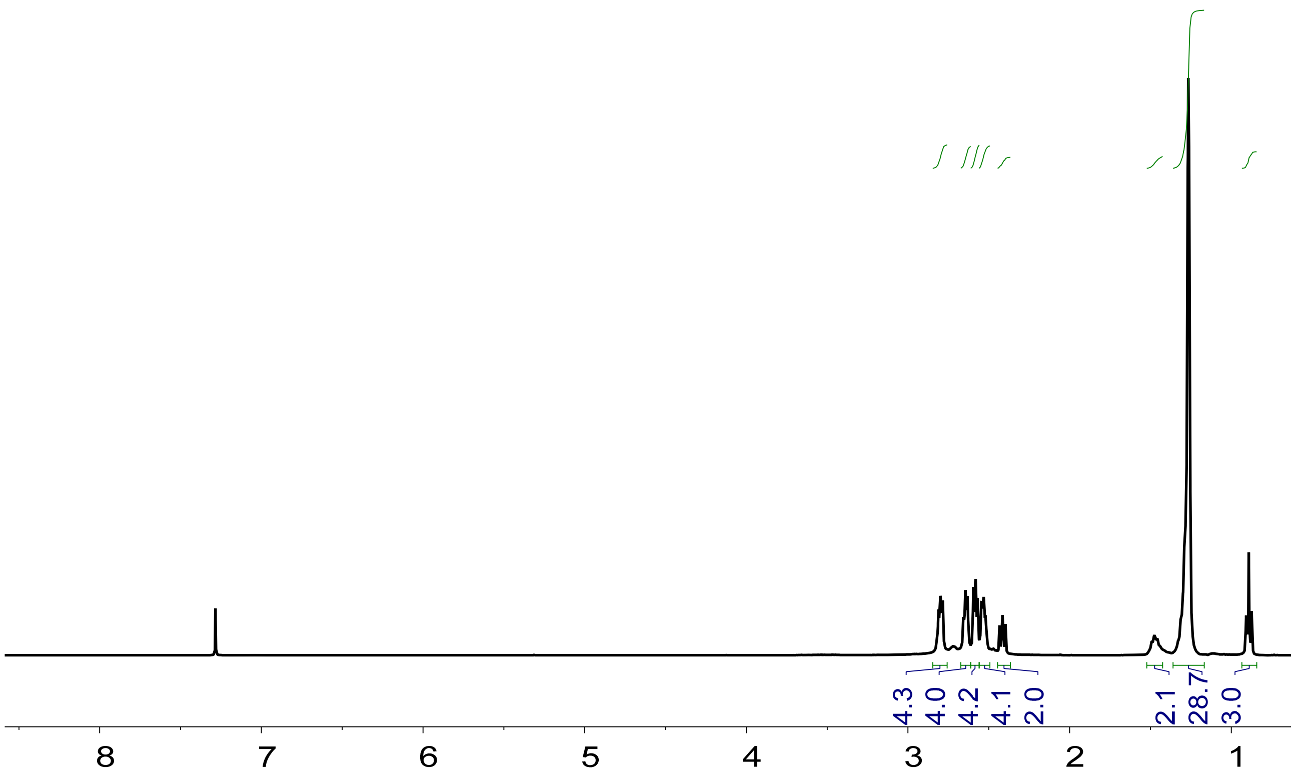

Figure S33. ^{1}H-NMR of **C_{16}Cyclen** in $CDCl_3$

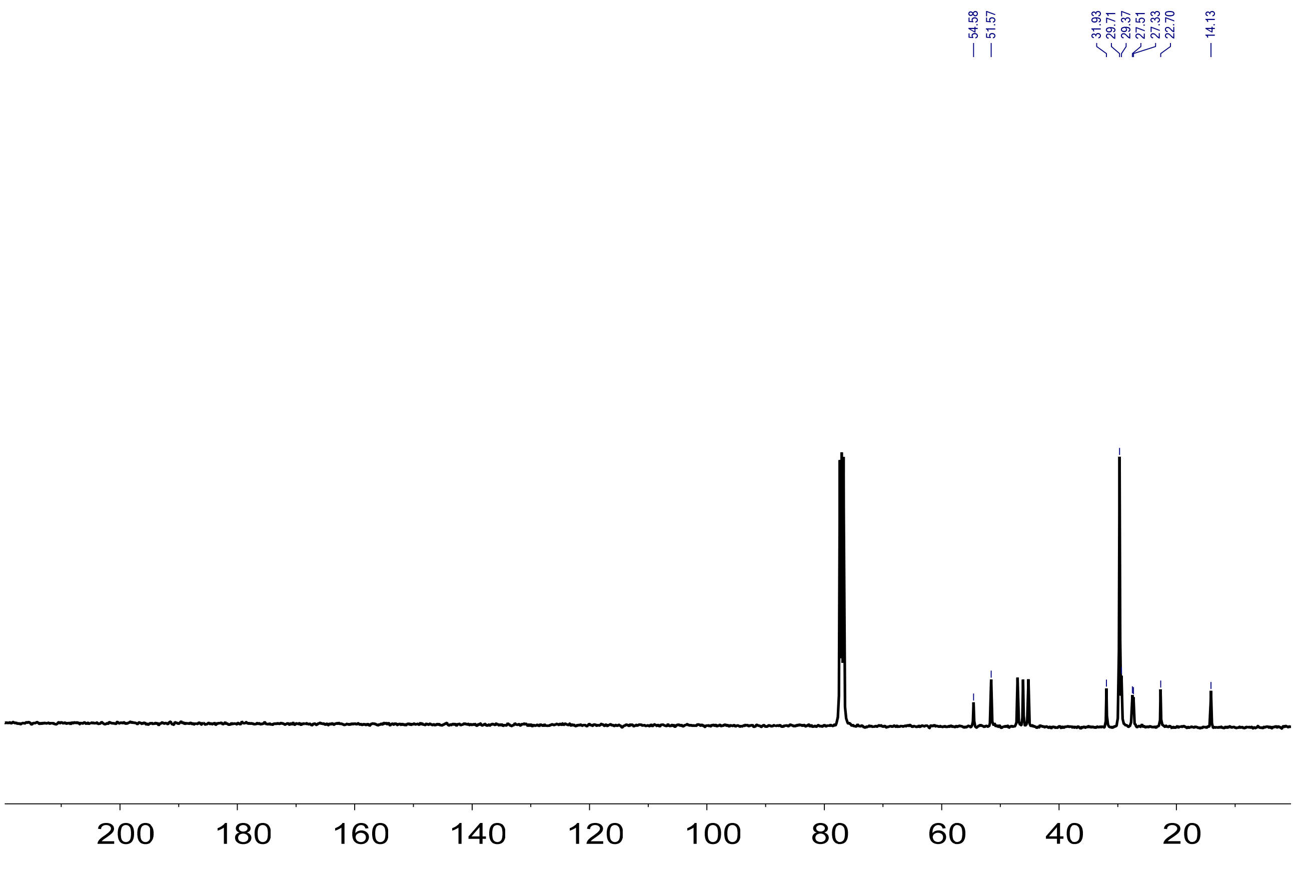

Figure S34. ^{13}C-NMR of **C_{16}Cyclen** in $CDCl_3$

4.2 Synthesis of C_{10F}TACN

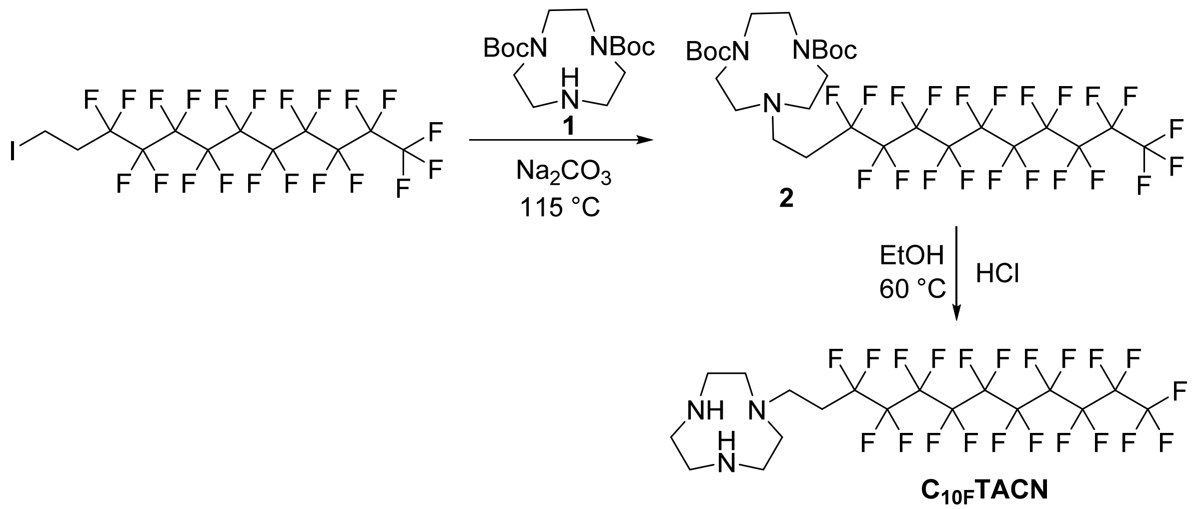

*2.2.1 Synthesis of **2***

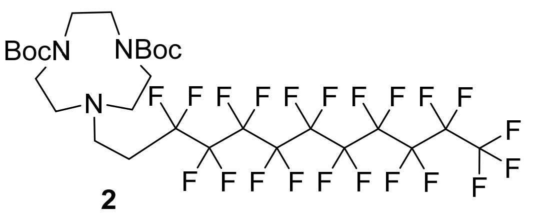

2

Protected compound **1** (116 mg; 0.352 mmol, 1 equiv, synthesized following the procedure reported in literature)[2] was mixed with 1-iodo-1H,1H,2H,2H-perfluorododecane (261 mg; 0.387 mmol, 1.1 equiv) and Na_2CO_3 (56 mg; 0.528 mmol, 1.5 equiv). The mixture was stirred into a closed vial for 18 hours at 115°C. The reaction crude was then purified through column chromatography (silica gel, eluent PE:EtOAc 9:1) and 90 mg of the desired compound **2** were obtained (yield 30%).

^{1}H NMR (δ ppm, 600 MHz, $CDCl_3$): δ 3.53 – 3.40 (m, 4H), 3.28 (d, J = 21.6 Hz, 4H), 2.96 – 2.82 (m, 2H), 2.75 – 2.62 (m, 4H), 2.26 (h, J = 13.4, 9.9 Hz, 2H), 1.48 (s, 18H).
^{13}C-NMR (151 MHz, $CDCl_3$) δ 155.63, 155.58, 155.41, 128.79, 127.81, 79.70, 79.62, 79.58, 79.50, 53.64, 53.46, 53.41, 51.14, 50.84, 50.80, 50.50, 49.89, 49.81, 49.70, 47.71, 47.52, 47.18, 28.48.
^{13}C-NMR (^{19}F-decoupled - 151 MHz, $CDCl_3$) δ 155.62, 155.41, 118.32, 118.01, 116.25, 114.45, 111.97, 111.14, 110.91, 110.77, 110.75, 110.22, 109.66, 109.19, 108.75, 108.51, 108.27, 108.03, 79.54, 77.91, 76.52, 54.32, 53.65, 53.43, 52.78, 52.55, 52.05, 51.72, 51.40, 51.14, 50.82, 50.51, 50.23, 49.90, 49.71, 49.61, 48.98, 48.78, 48.43, 47.69, 47.50, 47.19, 46.63, 46.31, 29.74, 28.90, 28.06, 27.22.
^{19}F-NMR (188 MHz, $CDCl_3$) δ -81.24 (t, J = 9.8 Hz), -114.30, -122.27, -123.20, -123.85, -126.62.
ESI MS (ESI+, H_2O:CH_3CN = 1:1). $[M+H-Boc]^+$: found: 776.4; calcd: 776.2.
(Mass measured by ESI-MS only shows mass of the compound missing one Boc group)

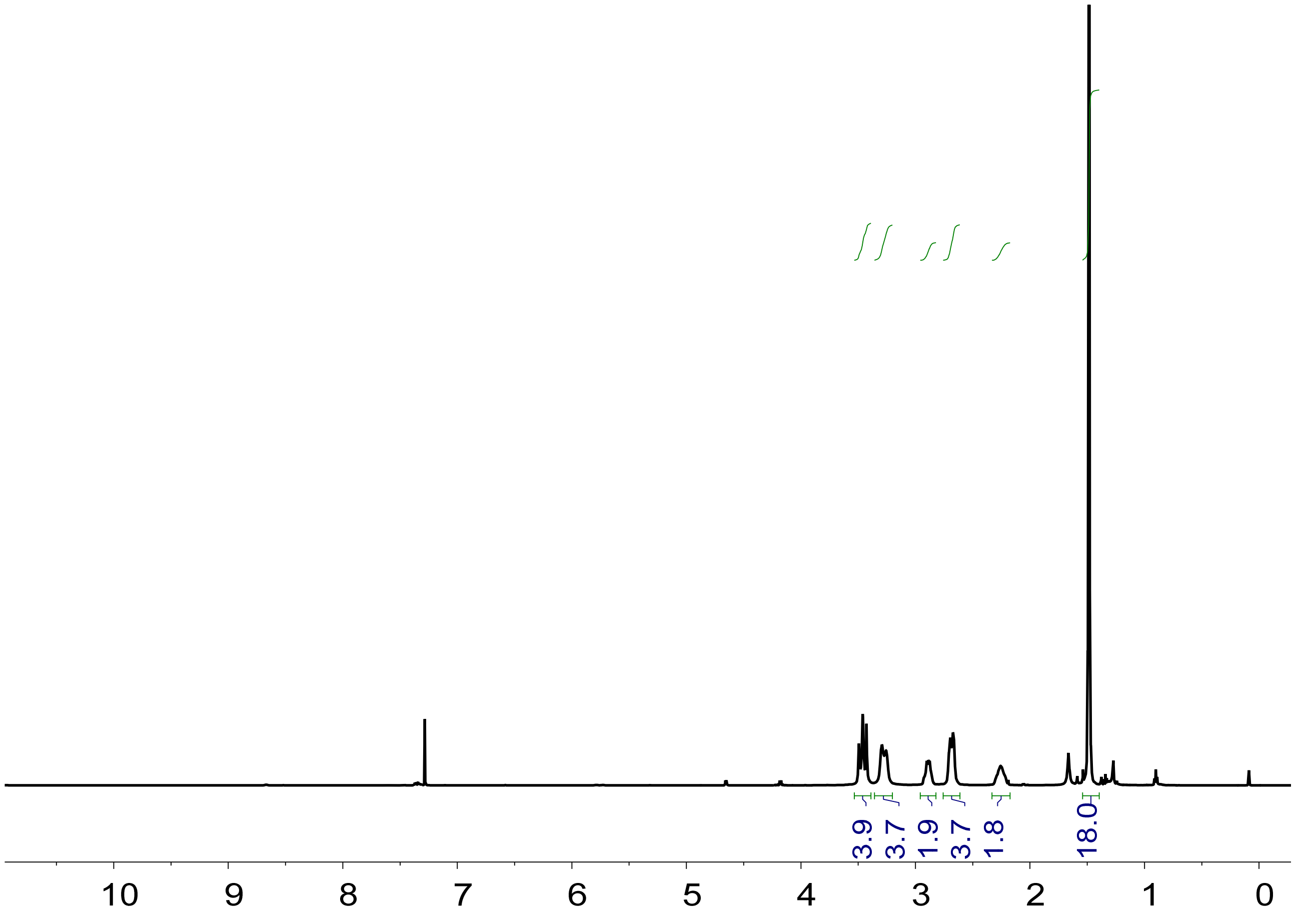

Figure S35. ^{1}H-NMR of **2** in $CDCl_3$

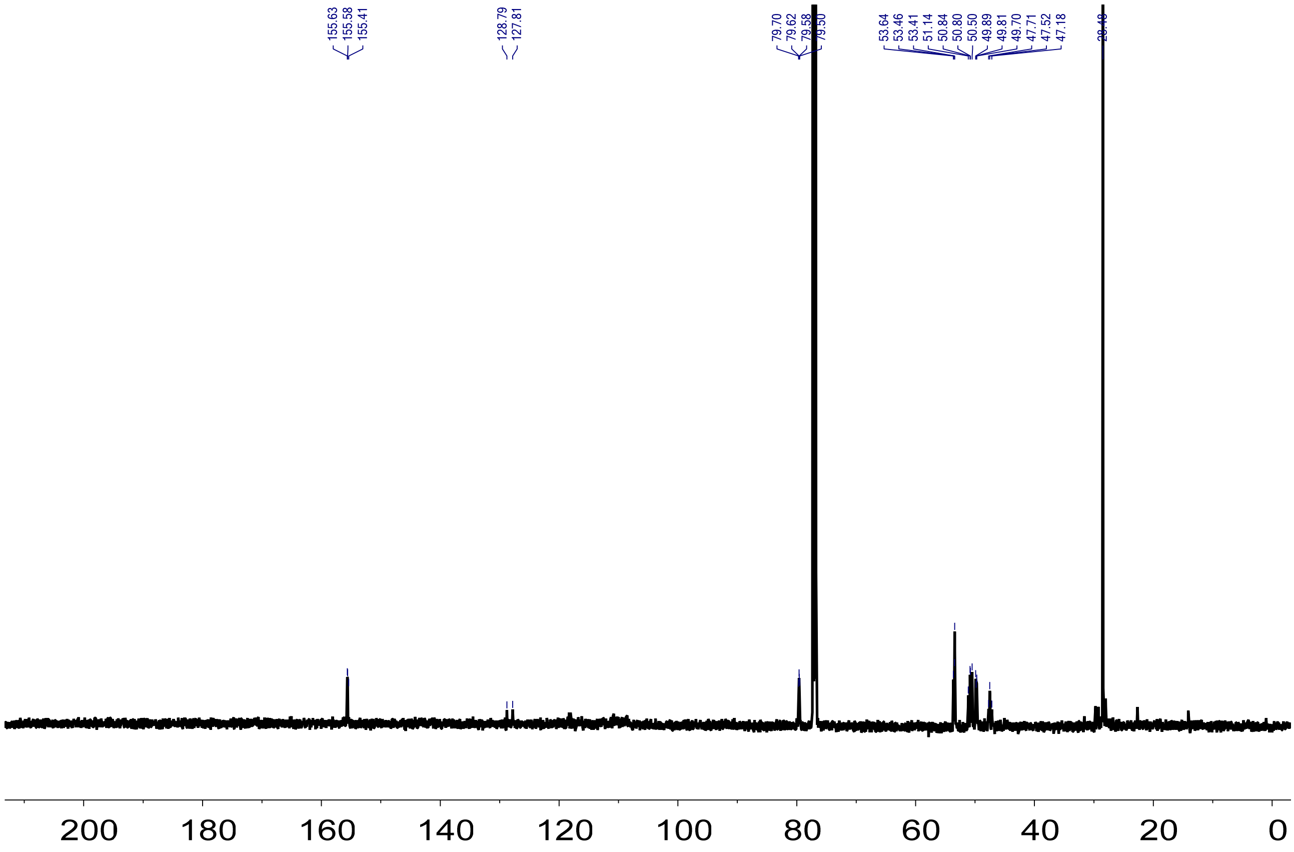

Figure S36. ^{13}C-NMR of **2** in $CDCl_3$

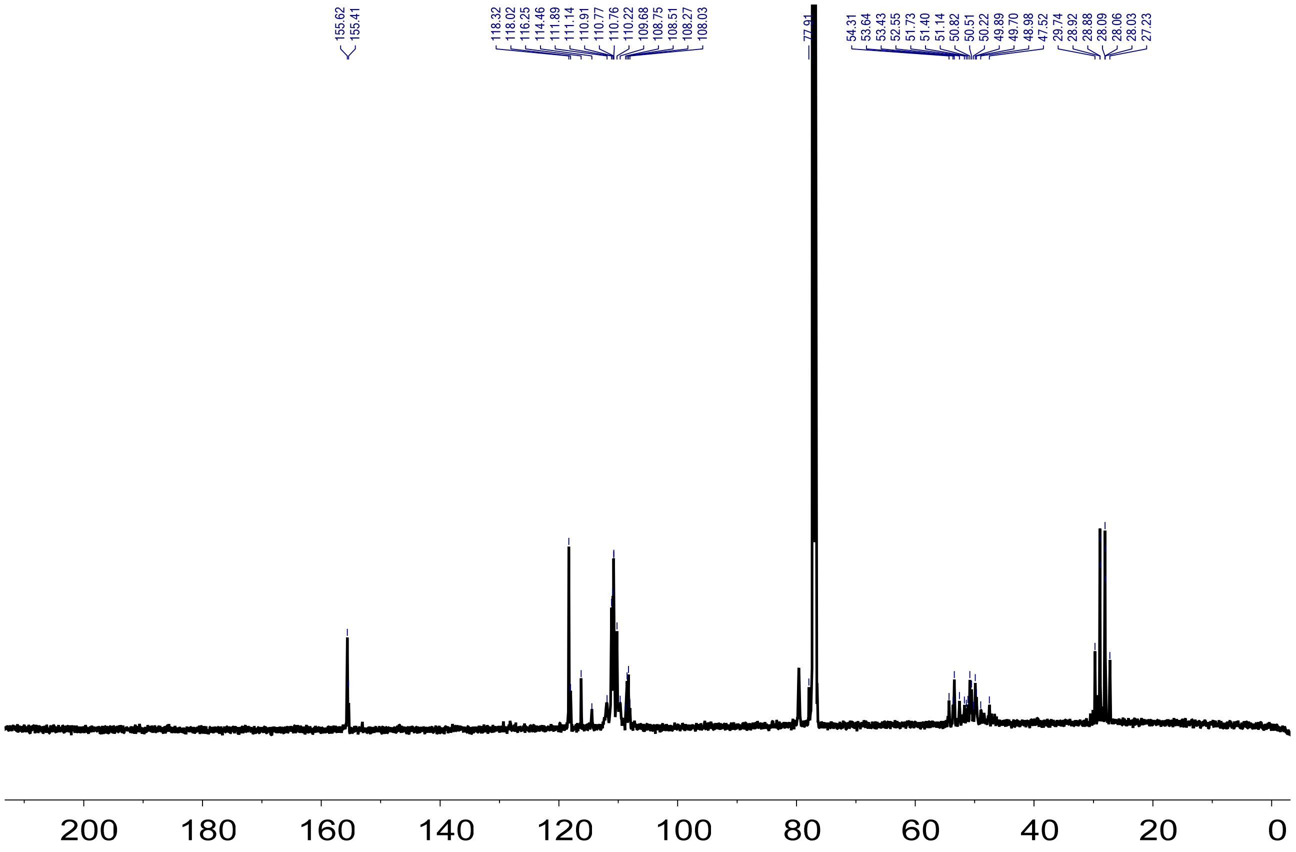

Figure S37. ^{13}C-NMR ^{19}F decoupled of **2** in $CDCl_3$

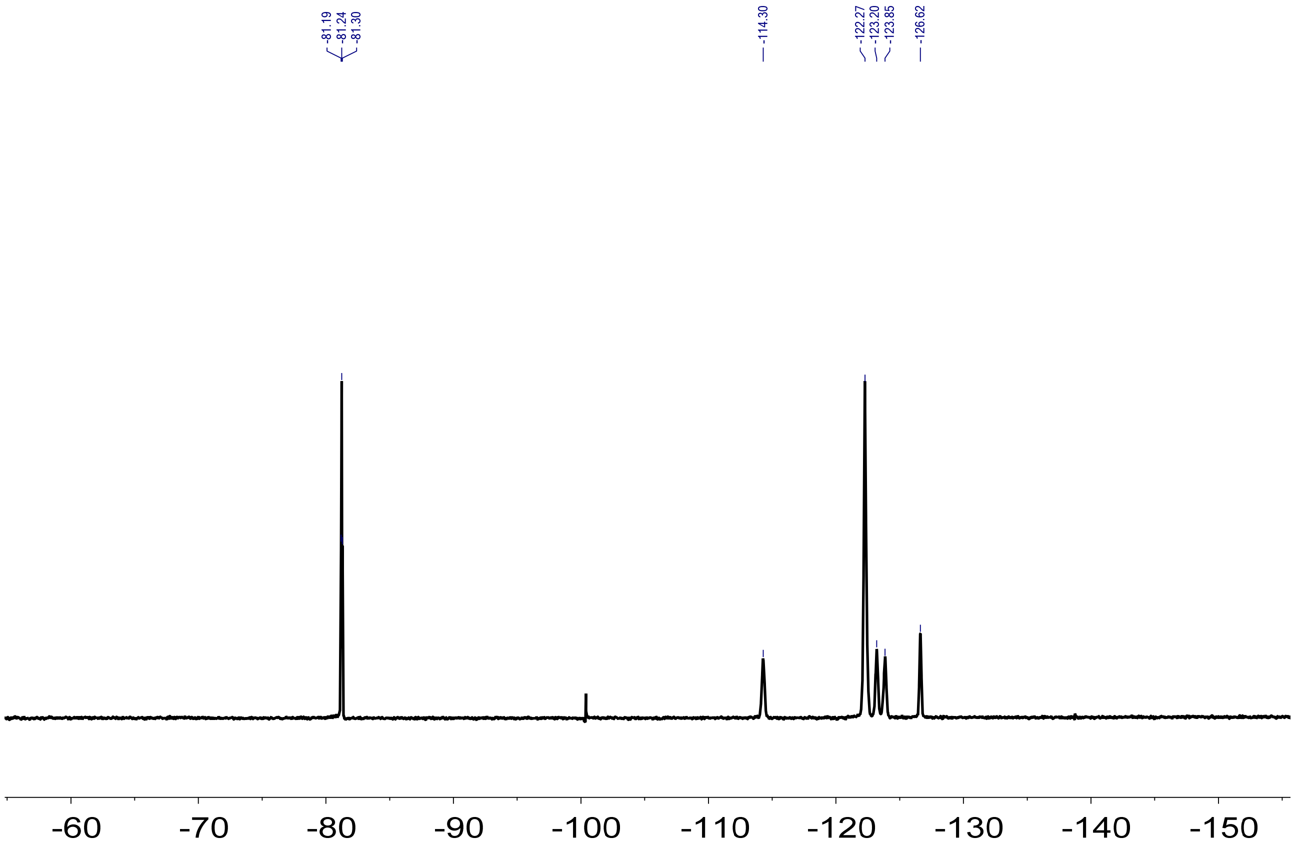

Figure S38. ^{19}F-NMR of **2** in $CDCl_3$

2.2.2 Synthesis of C_{10F}TACN

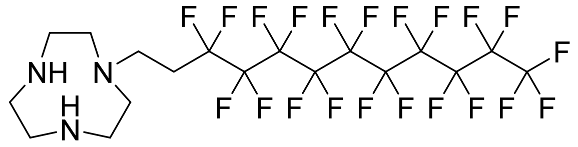

C_{10F}TACN

Compound **2** (90 mg, 0.103 mmol) was dissolved in 1.5 mL of EtOH and 0.5 mL of HCl 6 M. The mixture was stirred overnight at 60°C. Then the solvent was evaporated and the product **C_{10F}TACN** was obtained as a white solid chloride salt (70 mg, 99%).

^{1}H NMR (δ ppm, 600 MHz, MeOD) δ 3.69 – 3.63 (m, 4H), 3.37 – 3.35 (m, 4H), 3.16 (t, 2H), 3.04 – 3.01 (m, 4H), 2.61 (t, 2H).
^{13}C-NMR (^{19}F-decoupled, 151 MHz, MeOD) δ 118.71, 117.95, 116.16, 111.22, 110.86, 110.20, 108.51, 108.28, 48.63, 48.48, 48.34, 48.20, 46.85, 45.95, 45.79, 44.26, 43.31, 42.77, 42.35, 41.80, 40.84, 26.60, 25.74, 24.87.
^{13}C-NMR (151 MHz, MeOD) δ 46.85, 45.78, 43.31, 41.81, 25.74.
^{19}F-NMR (188 MHz, $CDCl_3$) δ -82.75 (t, *J* = 10.1 Hz), -115.30, -123.15, -124.12, -124.54, -127.70.
ESI MS (ESI+, H_2O:CH_3CN = 1:1). $[M+H]^+$: found: 676.1324; calcd: 676.1244

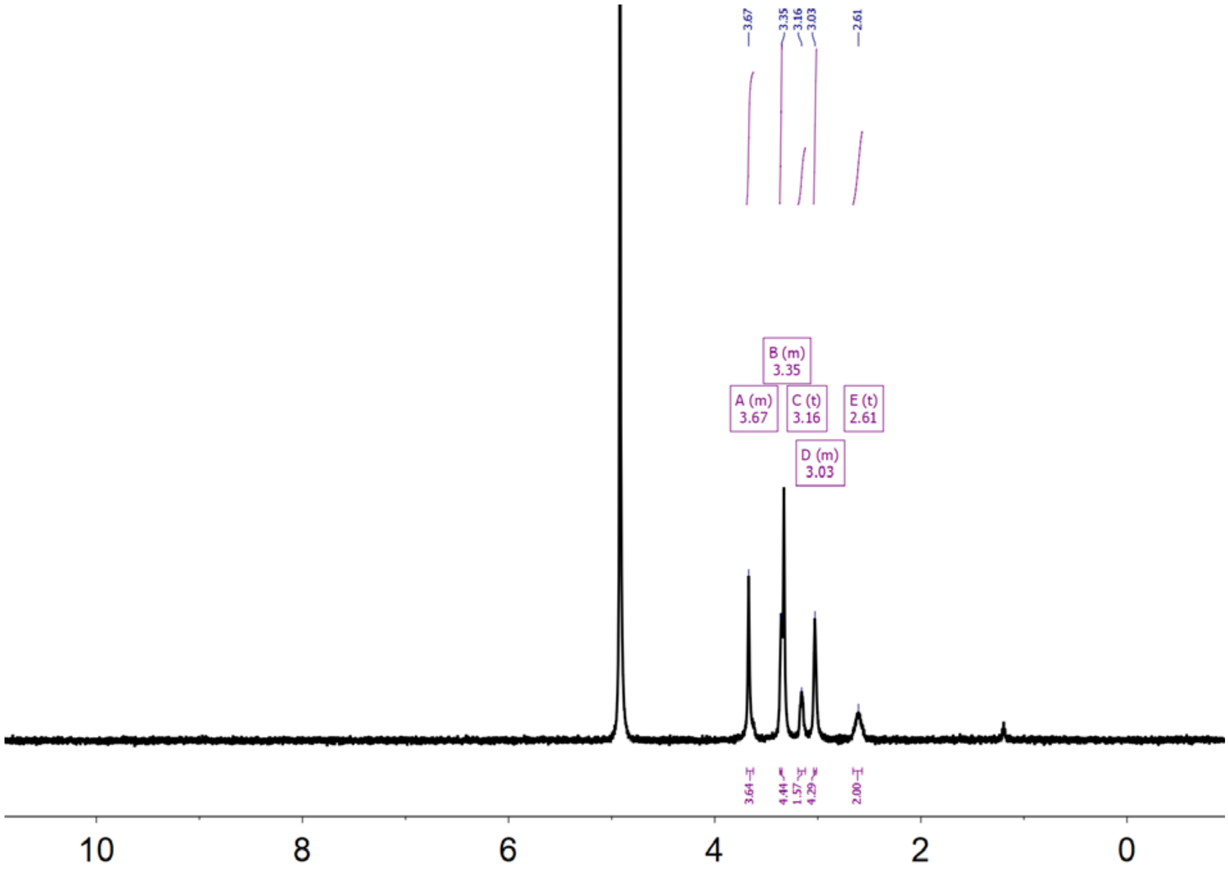

Figure S39. ^{1}H-NMR of **C$_{10F}$TACN** in MeOD

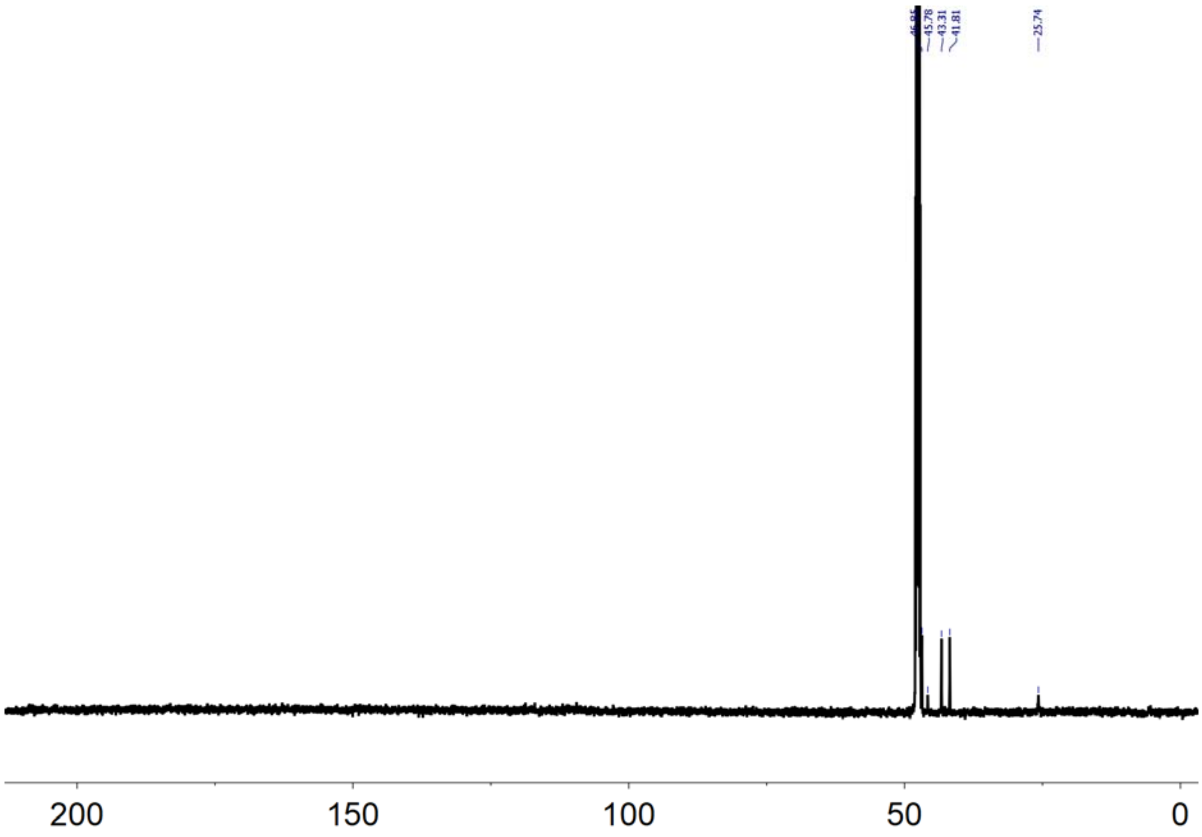

Figure S40. ^{13}C-NMR of **C$_{10F}$TACN** in MeOD

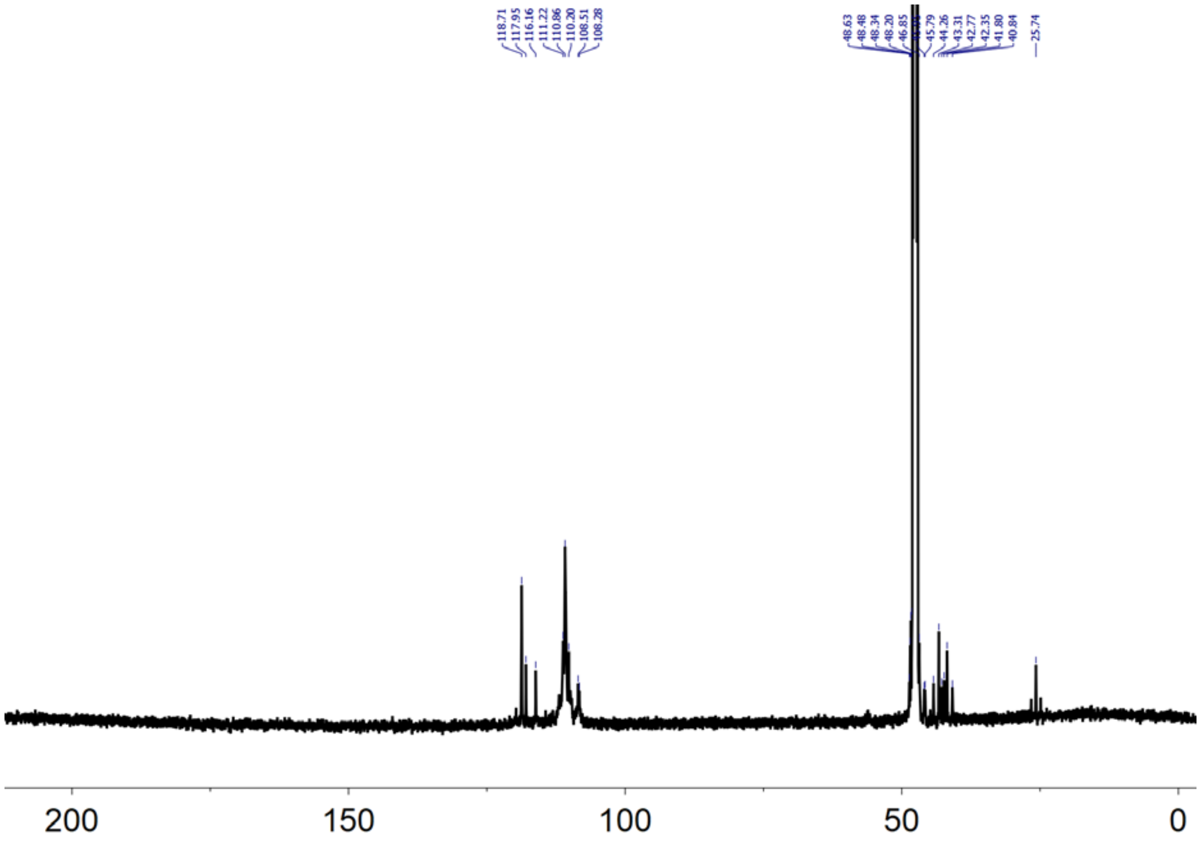

Figure S41. ^{13}C-NMR ^{19}F decoupled of **C_{10F}TACN** in MeOD

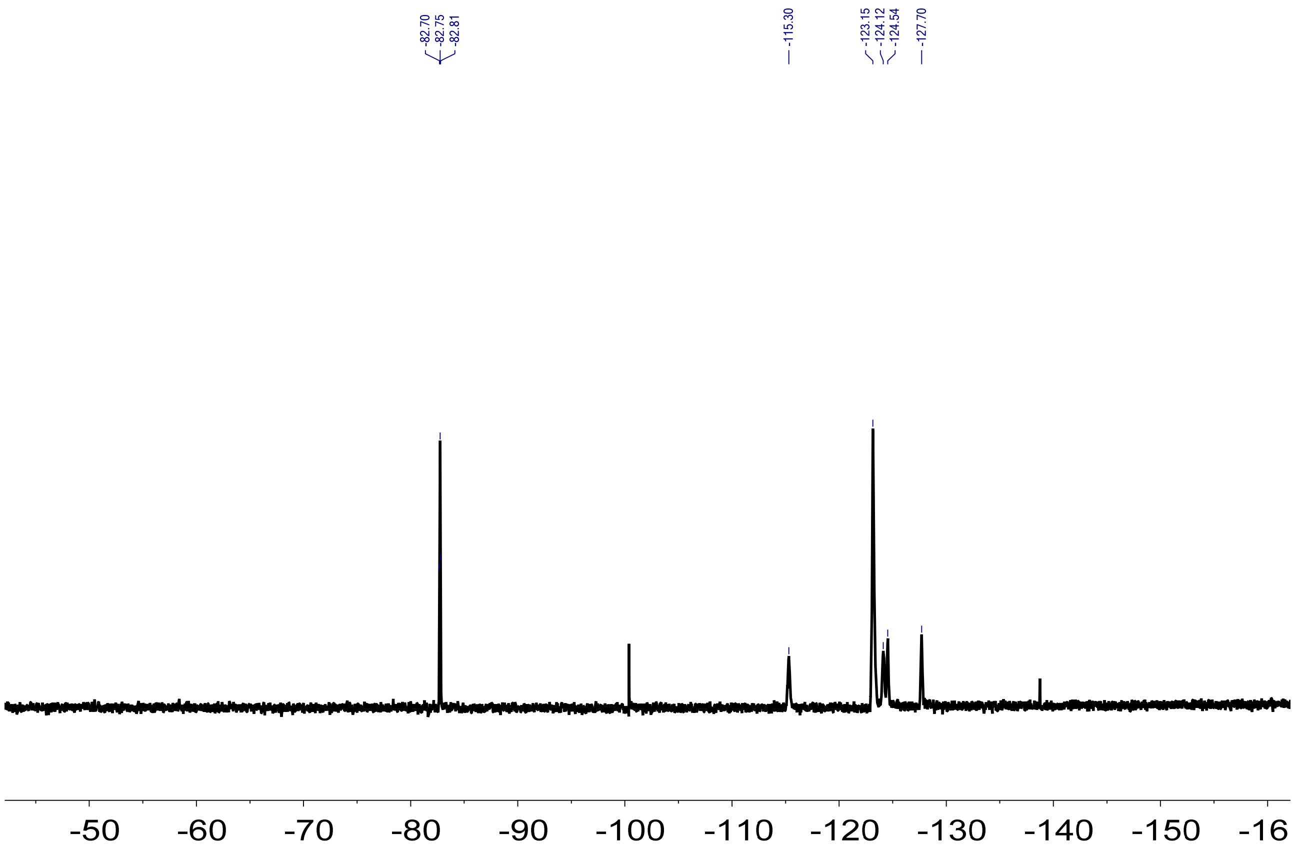

Figure S42. ^{19}F-NMR of **C_{10F}TACN** in $CDCl_3$

4.3 Synthesis of C_{4H}S-NBD

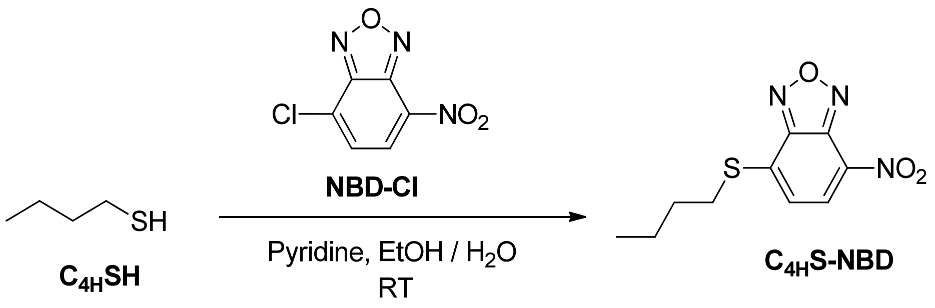

4-Chloro-7-nitrobenzofurazan (**NBD-Cl**, 99 mg, 0.496 mmol), 1-butanethiol (**C_{4H}SH**, 90 mg, 0.992 mmol), pyridine (118 μg, 1.448 mmol, 3 equiv) and 5 mL of EtOH/H_2O mixture (0.3/1) were stirred overnight at room temperature. The reaction mixture was then treated with brine and the crude product was extracted with EtOAc. After evaporation at reduced pressure, the product was purified using column chromatography (silica gel, eluent PE/EtOAc 8.5/1.5). **C_{4H}S-NBD** was obtained as an orange solid (101 mg, 0.399 mmol, yield 80%).

^{1}H NMR (δ ppm, 600 MHz, $CDCl_3$) δ 8.42 (d, *J* = 7.9 Hz, 1H), 7.18 (d, *J* = 7.9 Hz, 1H), 3.29 (t, *J* = 7.4 Hz, 2H), 1.93 – 1.78 (m, 2H), 1.60 (dt, *J* = 14.9, 7.4 Hz, 2H), 1.02 (t, *J* = 7.4 Hz, 3H).
^{13}C NMR (151 MHz, $CDCl_3$) δ 149.22, 142.50, 142.20, 132.44, 130.78, 120.21, 31.55, 29.86, 22.06, 13.55.
ESI-MS (ESI+, H_2O:ACN = 1:1). $[M+H]^+$: found: 254.0609; calcd: 254.0599

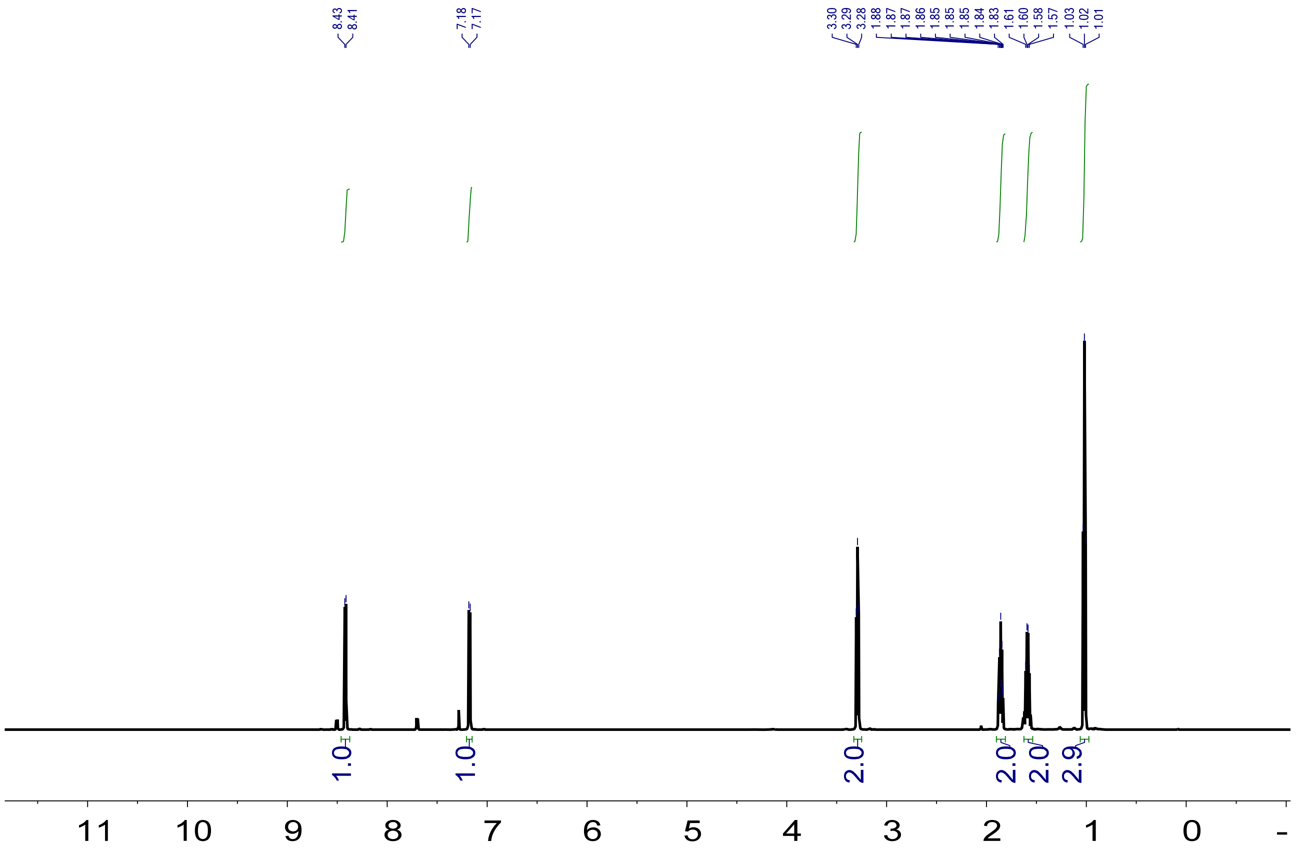

Figure S43. ^{1}H-NMR of **C$_{4H}$S-NBD** in $CDCl_3$

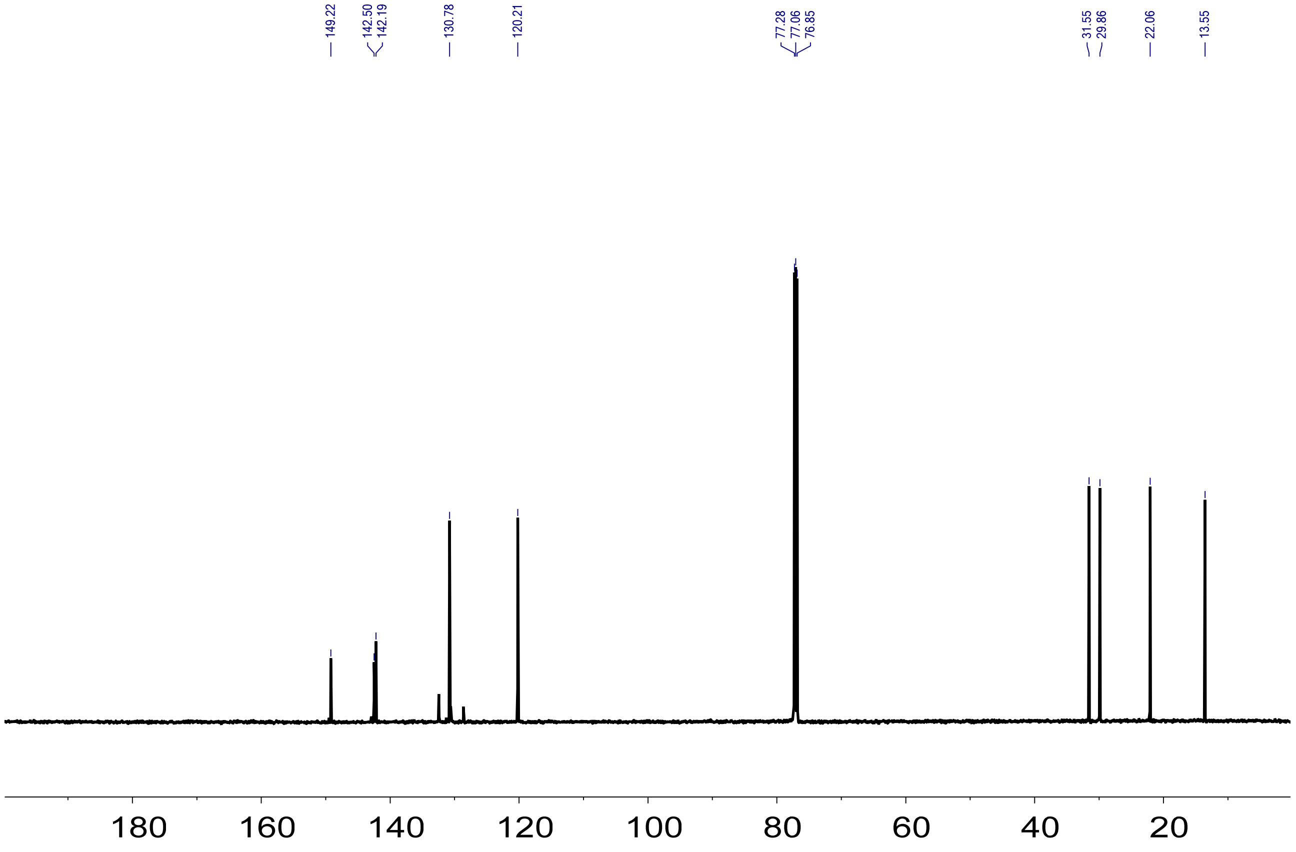

Figure S44. ^{13}C-NMR of **C$_{4H}$S-NBD** in $CDCl_3$

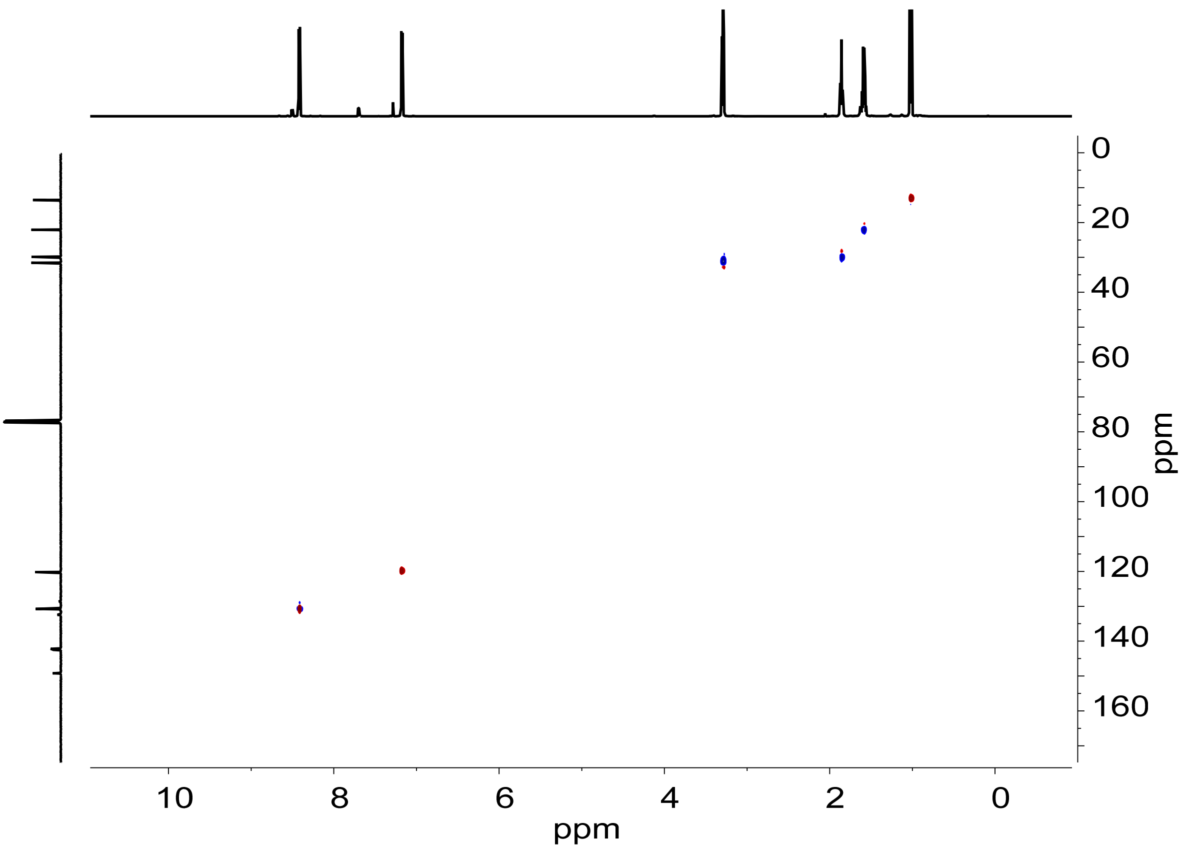

Figure S45. HSQC of $\mathbf{C_{4H}S}$**-NBD** in $CDCl_3$

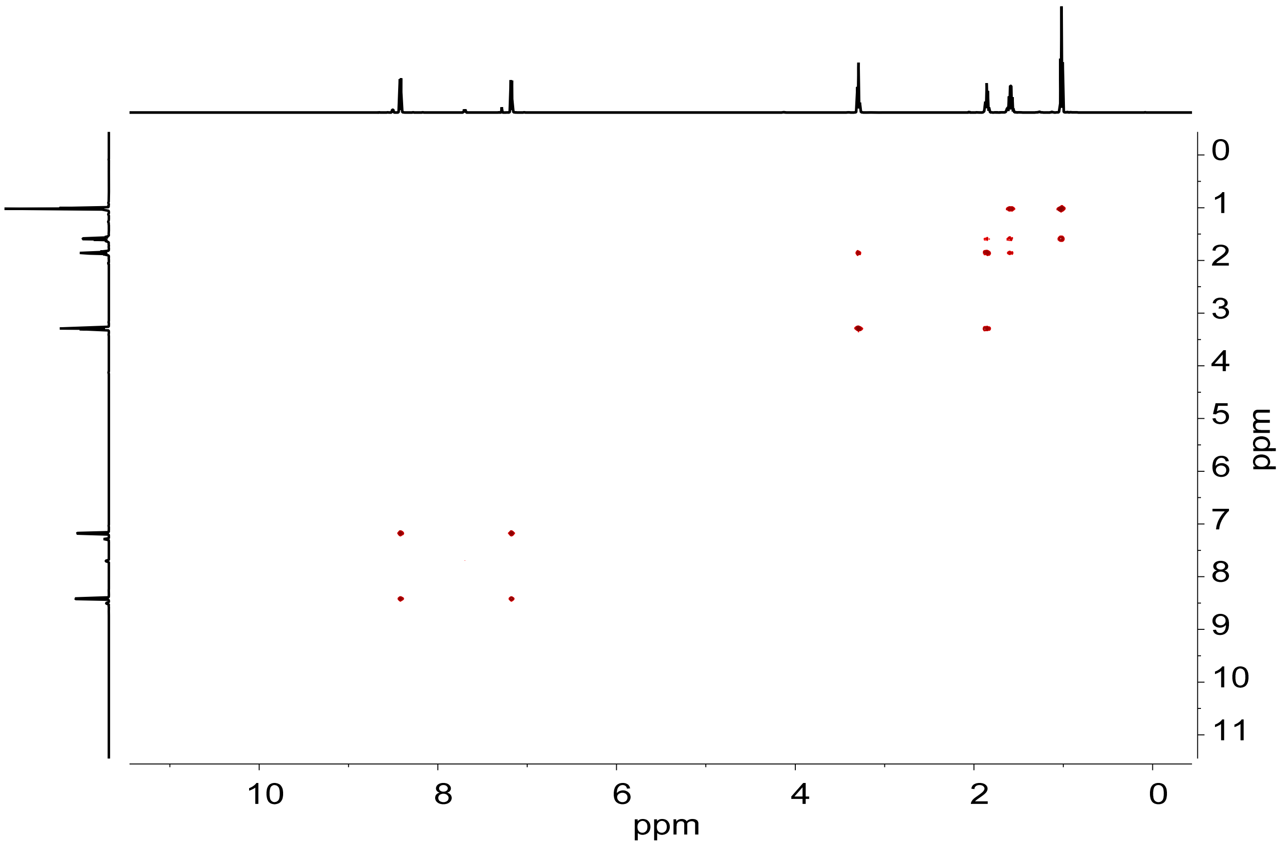

Figure S46. COSY of $\mathbf{C_{4H}S}$**-NBD** in $CDCl_3$

UV-Vis Spectra

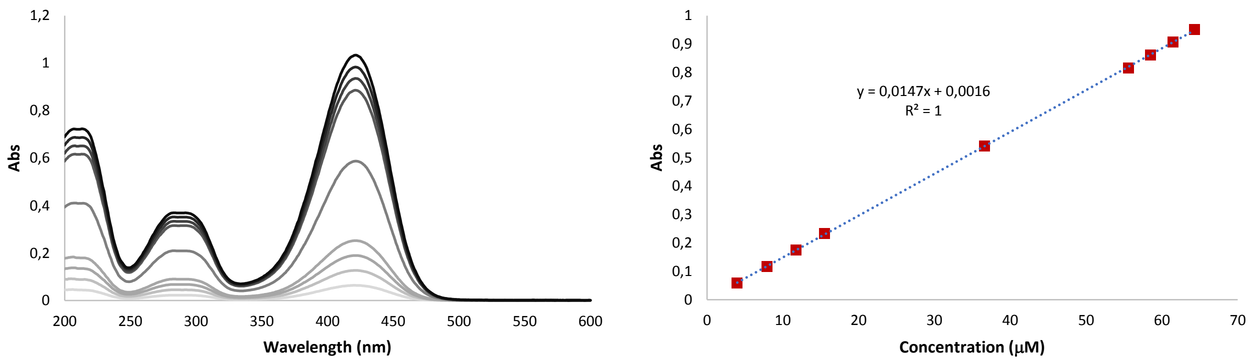

Figure S47. UV-Vis spectra of **C_{4H}S-NBD** in acetonitrile, at 4, 8, 12, 15, 37, 56, 59, 61, 64 μM and plot of the absorbance at 416 nm as a function of **C_{4H}S-NBD** concentration.

4.4 Synthesis of C_{6F}S-NBD

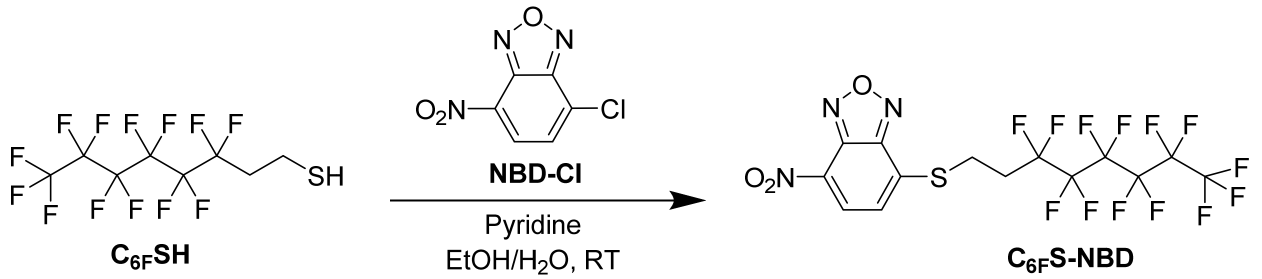

1H,1H,2H,2H-Perfluorodecanethiol (**C_{6F}SH**) (46 mg, 0.120 mmol, 1.2 equiv), 4-chloro-7-nitrobenzofurazan (**NBD-Cl**) (20 mg, 0.100 mmol, 1 equiv) and pyridine (24 μl, 0.300 mmol, 3 equiv) were added into 1mL of EtOH/H_2O (0.3/1) and the mixture was stirred at room temperature overnight. The reaction mixture was washed with brine and the crude product extracted with EtOAc. After evaporation under reduced pressure, the crude product was purified via flash column chromatography (20% EtOAc/PE) to obtain **C_{6F}S-NBD** as an orange solid (24 mg, yield 44%).

^{1}H NMR (600 MHz, $CDCl_3$) δ 8.47 (d, *J* = 7.8 Hz, 1H), 7.27 (d, *J* = 7.8 Hz, 1H), 3.70 – 3.49 (m, 2H), 2.71 – 2.61 (m, 2H).

^{13}C NMR (151 MHz, $CDCl_3$) δ 149.19, 142.56, 138.68, 133.68, 130.37, 121.59, 30.73(t), 22.82.

^{13}C NMR (^{19}F-decoupled, 151 MHz, $CDCl_3$) δ 149.19 (d, *J* = 9.6 Hz), 142.56 (d, *J* = 8.0 Hz), 138.68 (dt, *J* = 8.7, 4.3 Hz), 133.68 (dd, *J* = 8.2, 4.8 Hz), 130.37 (d, *J* = 168.9 Hz), 121.59 (d, *J* = 168.2 Hz), 118.03, 117.26, 116.26, 110.86 (d, *J* = 26.7 Hz) 110.19, 108.42 (d, *J* = 36.4 Hz), 30.72 (t, *J* = 133.1 Hz), 22.82 (t, *J* = 144.0 Hz).

^{19}F NMR (565 MHz, $CDCl_3$) δ -80.78, -114.11, -121.73, -122.77, -123.11, -126.06.

ESI-MS: (ESI+, CH_3CN). $[M+H]^+$: found: 544.0017; calcd: 544.0001

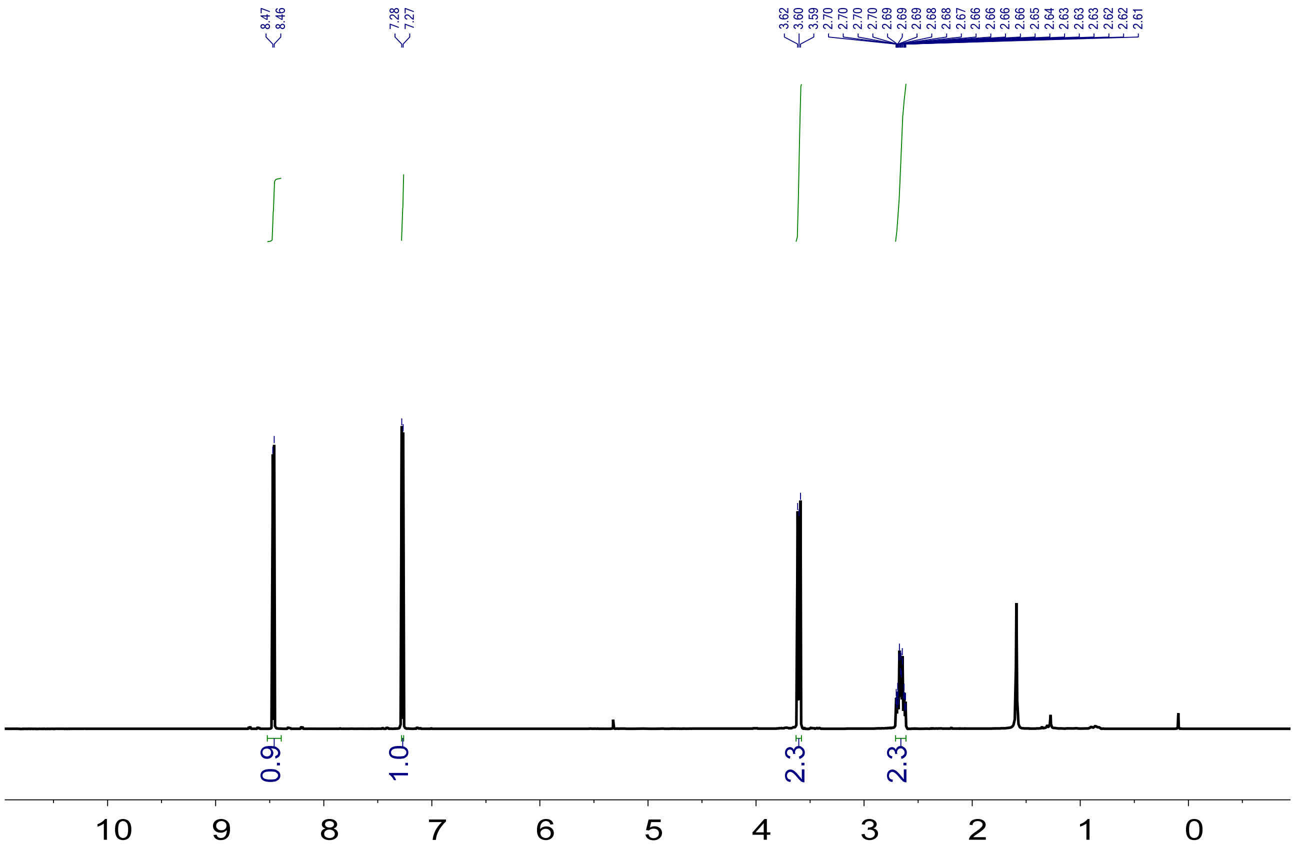

Figure S48. ^{1}H-NMR of **C_{6F}S-NBD** in $CDCl_3$

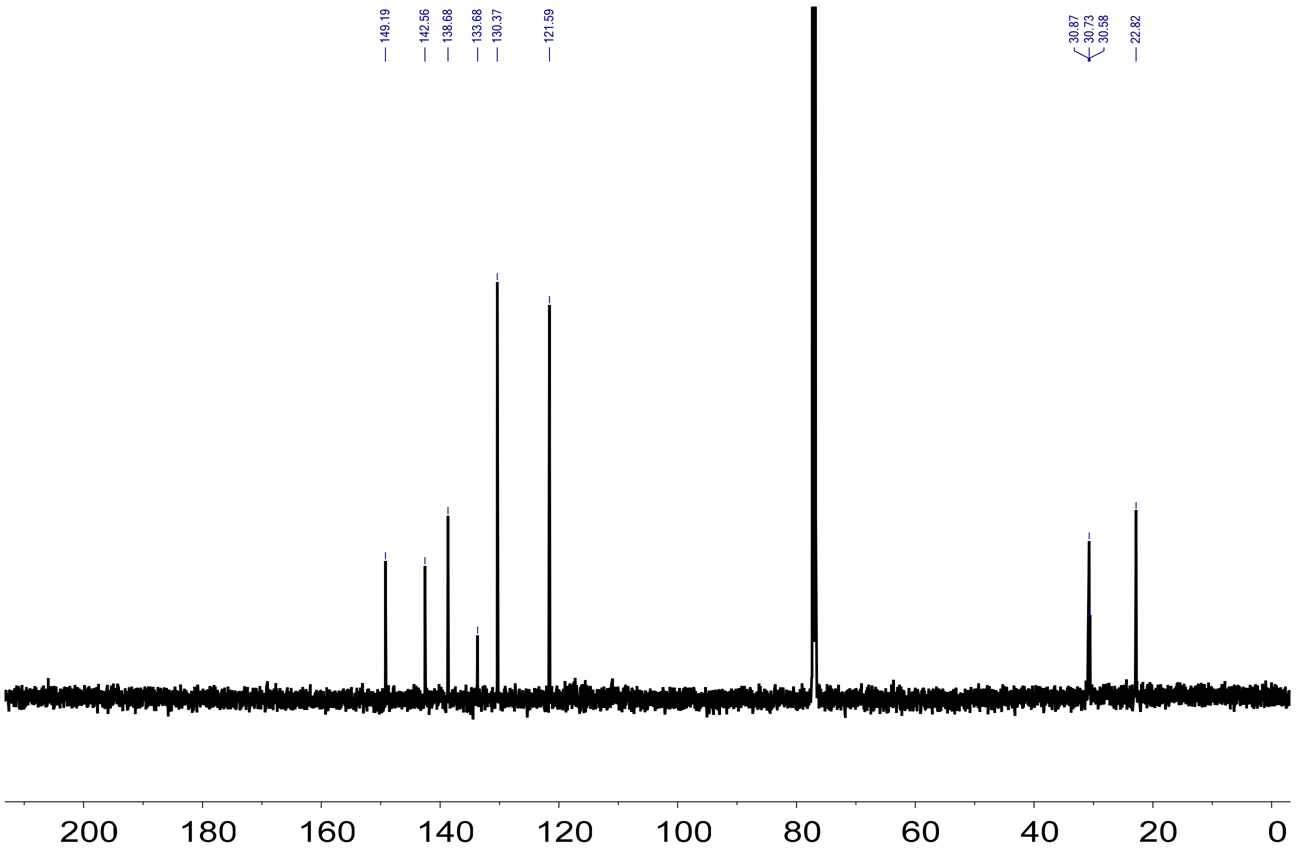

Figure S49. ^{13}C-NMR of **C_{6F}S-NBD** in $CDCl_3$

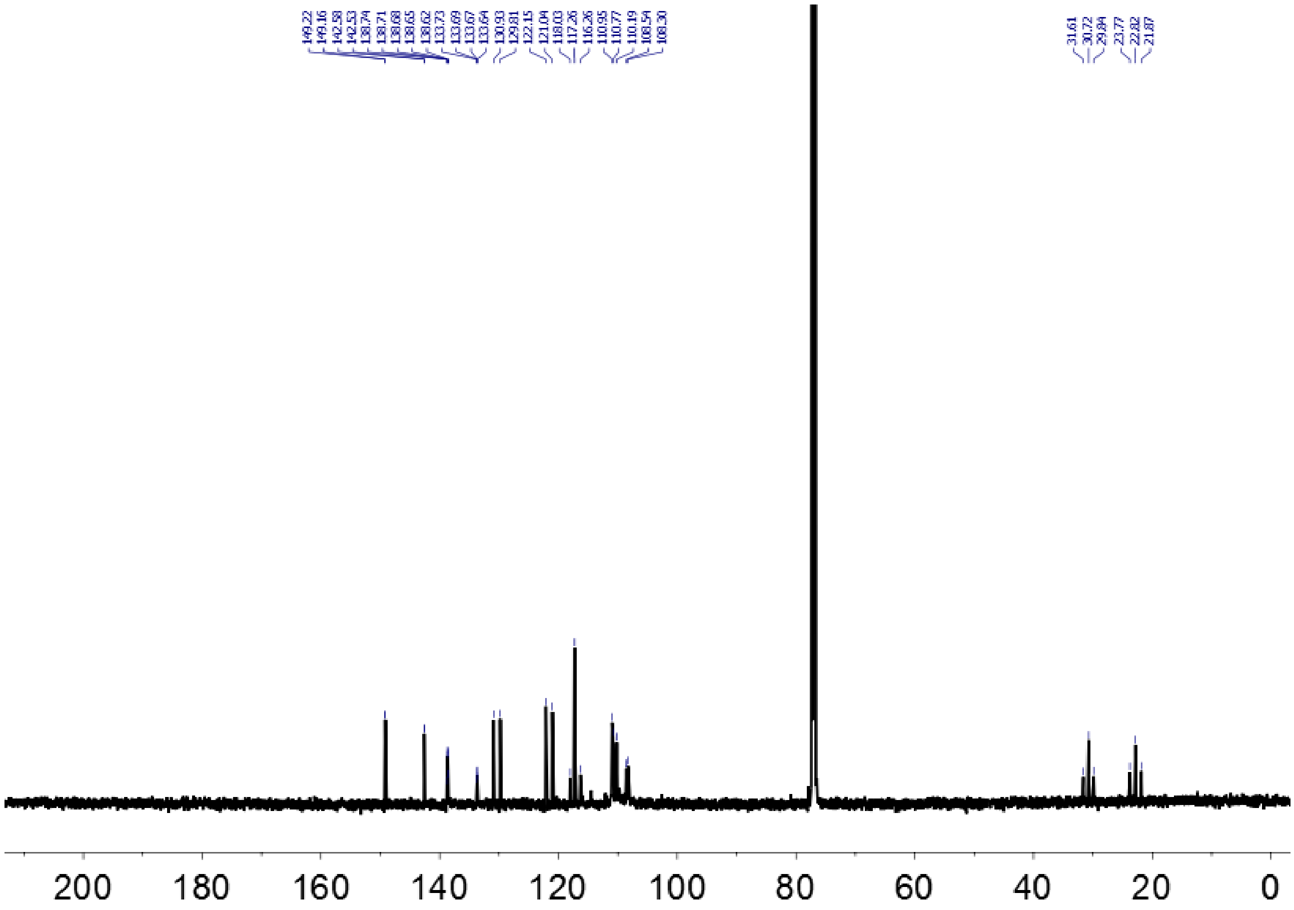

Figure S50. ^{13}C-NMR ^{19}F-decoupled of **C_{6F}S-NBD** in $CDCl_3$

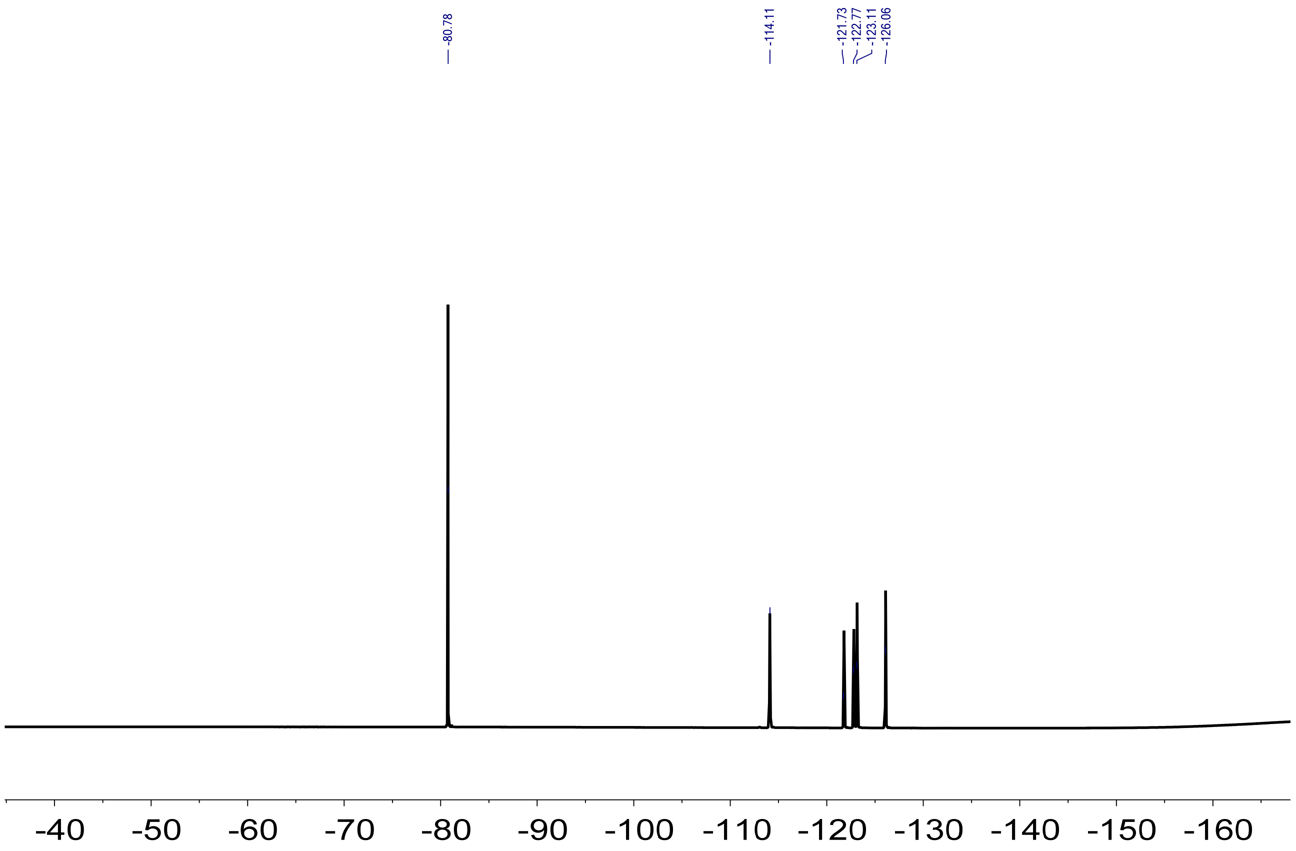

Figure S51. ^{19}F-NMR of **C_{6F}S-NBD** in $CDCl_3$

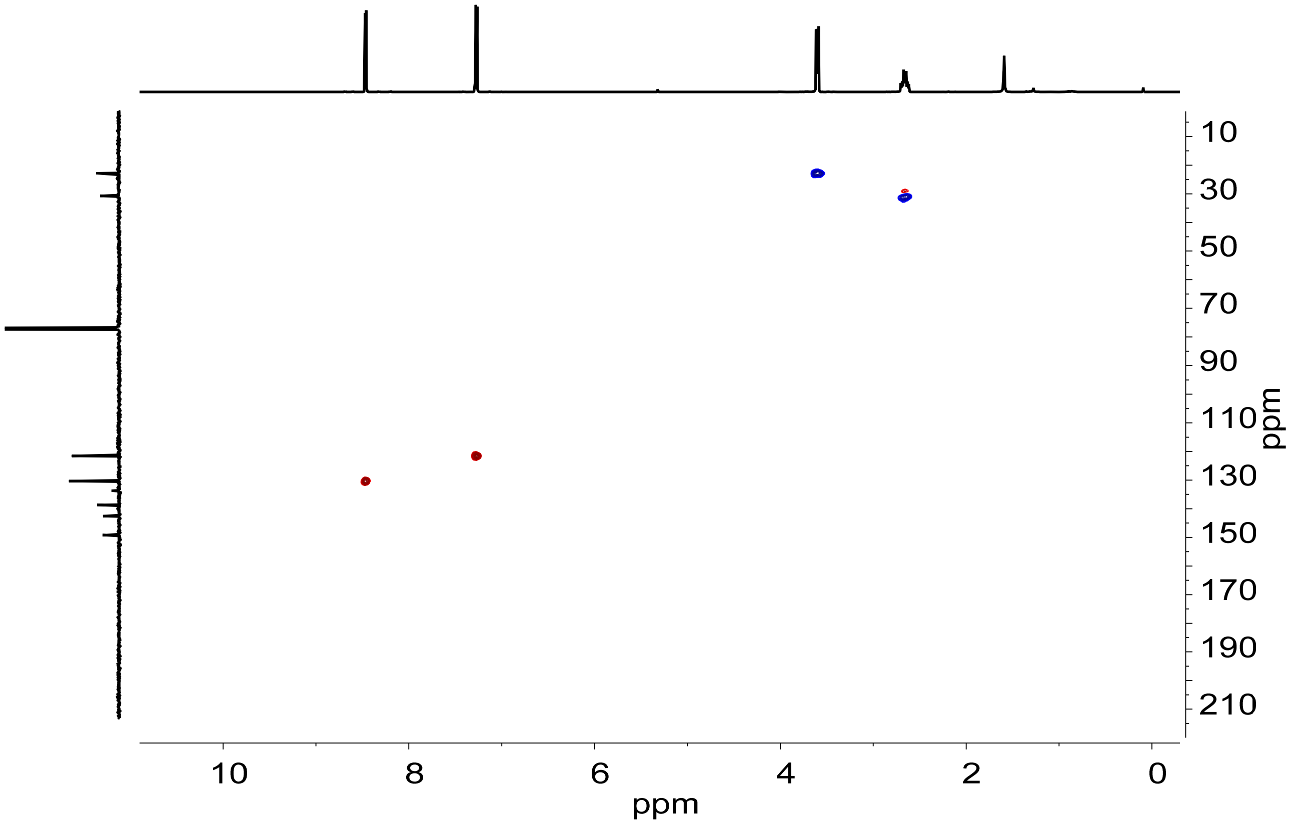

Figure S52: HSQC of **C_{6F}S-NBD** in $CDCl_3$

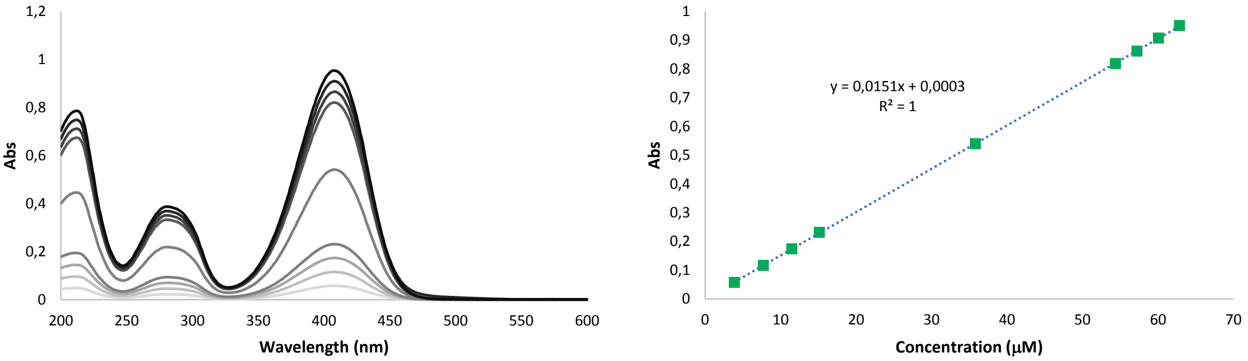

Figure S53. UV-Vis spectra of **C_{6F}S-NBD** in acetonitrile, at 4, 8, 11, 15, 36, 54, 57, 60, 63 µM and plot of the absorbance at 416 nm as a function of **C_{6F}S-NBD** concentration.

5. References

1. S. Chandrabhas, S. Maiti, I. Fortunati, C. Ferrante, L. Gabrielli, L. J. Prins, *Angew. Chem. Int. Ed.* **2020**, *59*, 22223

2. G. Pieters, A. Cazzolaro, R. Bonomi, L. J. Prins, *Chem. Commun*. **2012**, *48*, 1916
